# Comparison of Treatments for Frozen Shoulder: A Systematic Review and Meta-analysis

**DOI:** 10.1001/jamanetworkopen.2020.29581

**Published:** 2020-12-16

**Authors:** Dimitris Challoumas, Mairiosa Biddle, Michael McLean, Neal L. Millar

**Affiliations:** 1 Institute of Infection, Immunity and Inflammation, College of Medicine, Veterinary and Life Sciences, University of Glasgow, Scotland, United Kingdom

## Abstract

**Importance:**

There are a myriad of available treatment options for patients with frozen shoulder, which can be overwhelming to the treating health care professional.

**Objective:**

To assess and compare the effectiveness of available treatment options for frozen shoulder to guide musculoskeletal practitioners and inform guidelines.

**Data Sources:**

Medline, EMBASE, Scopus, and CINHAL were searched in February 2020.

**Study Selection:**

Studies with a randomized design of any type that compared treatment modalities for frozen shoulder with other modalities, placebo, or no treatment were included.

**Data Extraction and Synthesis:**

Data were independently extracted by 2 individuals. This study followed the Preferred Reporting Items for Systematic Reviews and Meta-analyses (PRISMA) reporting guideline. Random-effects models were used.

**Main Outcomes and Measures:**

Pain and function were the primary outcomes, and external rotation range of movement (ER ROM) was the secondary outcome. Results of pairwise meta-analyses were presented as mean differences (MDs) for pain and ER ROM and standardized mean differences (SMDs) for function. Length of follow-up was divided into short-term (≤12 weeks), mid-term (>12 weeks to ≤12 months), and long-term (>12 months) follow-up.

**Results:**

From a total of 65 eligible studies with 4097 participants that were included in the systematic review, 34 studies with 2402 participants were included in pairwise meta-analyses and 39 studies with 2736 participants in network meta-analyses. Despite several statistically significant results in pairwise meta-analyses, only the administration of intra-articular (IA) corticosteroid was associated with statistical and clinical superiority compared with other interventions in the short-term for pain (vs no treatment or placebo: MD, −1.0 visual analog scale [VAS] point; 95% CI, −1.5 to −0.5 VAS points; *P* < .001; vs physiotherapy: MD, −1.1 VAS points; 95% CI, −1.7 to −0.5 VAS points; *P* < .001) and function (vs no treatment or placebo: SMD, 0.6; 95% CI, 0.3 to 0.9; *P* < .001; vs physiotherapy: SMD 0.5; 95% CI, 0.2 to 0.7; *P* < .001). Subgroup analyses and the network meta-analysis demonstrated that the addition of a home exercise program with simple exercises and stretches and physiotherapy (electrotherapy and/or mobilizations) to IA corticosteroid may be associated with added benefits in the mid-term (eg, pain for IA coritocosteriod with home exercise vs no treatment or placebo: MD, −1.4 VAS points; 95% CI, −1.8 to −1.1 VAS points; *P* < .001).

**Conclusions and Relevance:**

The findings of this study suggest that the early use of IA corticosteroid in patients with frozen shoulder of less than 1-year duration is associated with better outcomes. This treatment should be accompanied by a home exercise program to maximize the chance of recovery.

## Key Points

**Question:**

Are any treatment modalities for frozen shoulder associated with better outcomes than other treatments?

**Findings:**

In this meta-analysis of 65 studies with 4097 participants, intra-articular corticosteroid was associated with increased short-term benefits compared with other nonsurgical treatments, and its superiority appeared to last for as long as 6 months. The addition of a home exercise program and/or electrotherapy or passive mobilizations may be associated with added benefits.

**Meaning:**

The results of this study suggest that intra-articular corticosteroid should be offered to patients with frozen shoulder at first contact.

## Teaser

This systematic review and meta-analysis assesses and compares the effectiveness of available treatment options for frozen shoulder to guide musculoskeletal practitioners and inform guidelines.

## Introduction

Adhesive capsulitis, also known as frozen shoulder, is a common shoulder concern manifesting in progressive loss of glenohumeral movements coupled with pain.^1^ It is a fibroproliferative tissue fibrosis, and although the immunobiological advances in other diseases have helped dissect the pathophysiology of this condition, overall, the molecular mechanisms underpinning it remain poorly understood.^2,3,4,5^

Frozen shoulder manifests clinically as shoulder pain with progressive restricted movement, both active and passive, along with normal radiographic scans of the glenohumeral joint.^6^ It classically progresses prognostically through 3 overlapping stages of pain (stage 1, lasting 2-9 months), stiffness (stage 2, lasting 4-12 months), and recovery (stage 3, lasting 5-24 months).^7^ However, this is an estimated time frame, and many patients can still experience symptoms at 6 years.^8^ A primary care–based observational study estimated its incidence as 2.4 per 100 000 individuals per year,^9^ with prevalence varying from less than 1% to 2% of the population.^10^

A true evidence-based model for its medical management has not been defined, with a wide spectrum of operative and nonoperative treatments available. From the international to departmental level, management strategies vary widely, reflecting the lack of good-quality evidence.^11^ The British Elbow and Shoulder Society/British Orthopaedic Association (BESS/BOA) has published recommendations in a patient care pathway for frozen shoulder, with a step-up approach in terms of invasiveness advised.^12^ The UK Frozen Shoulder Trial, a randomized parallel trial comparing the clinical and cost-effectiveness of early structured physiotherapy, manipulation under anesthetic (MUA), and arthroscopic capsular release (ACR) is currently under way.^13^ The aim of this systematic review is to present the available evidence relevant to treatment and outcomes for frozen shoulder with the ultimate objective of guiding clinical practice, both in primary and secondary care.

## Methods

The present systematic review has been conducted and authored according to the Preferred Reporting Items for Systematic Reviews and Meta-analyses (PRISMA) reporting guideline.^14^ Our patient, intervention, comparison, and outcome (PICO) was defined as follows: patients, patients with frozen shoulder of any etiology, duration, and severity; intervention, any treatment modality for frozen shoulder; comparison, any other treatment modality, placebo, or no treatment; and outcome, pain and function (primary outcomes) and external rotation range of movement (ER ROM) (secondary outcome) in the short term, midterm, or long term.

### Eligibility

Included studies had a randomized design of any type and compared treatment modalities for frozen shoulder with other treatment modalities, placebo, or no treatment. Additionally, at least 1 of our preset outcome measures needed to be included in the study. Studies that compared different types, regimens, dosages, or durations of the same intervention were excluded (eg, different doses of corticosteroid or different exercise types). Those assessing the effectiveness of the same modality applied in different anatomical sites (eg, subacromial vs intra-articular [IA] corticosteroid) were included. Participants had to be older than 18 years with a clinical diagnosis of adhesive capsulitis. No formal diagnostic criteria were used to define frozen shoulder; however, the use of inappropriate or inadequate diagnostic criteria was taken into account in risk-of-bias assessments. Duration of the condition was not a criterion nor were previous treatments and follow-up. Inclusion of patients with specific conditions (eg, diabetes) was not an exclusion criterion, and it was not taken into account in analyses, provided that their proportion in the treatment groups was comparable.

Nonrandomized comparative studies, observational studies, case reports, case series, literature reviews, published conference abstracts, and studies published in languages other than English were excluded. Studies including patients with the general diagnosis of shoulder pain were also excluded even if a proportion of them had frozen shoulder. Studies assessing the effectiveness of different types of physiotherapy-led interventions, exercise, or stretching regimens were also excluded.

### Search Strategy

A thorough literature search was conducted by 3 of us (D.C., M.B., and M.M.) via Medline, EMBASE, Scopus, and CINAHL in February 2020, with the following Boolean operators in all fields: (*adhesive capsulitis* OR *frozen shoulder* OR *shoulder periarthritis*) AND (*treatment* OR *management* OR *therapy*) AND randomi*). Relevant review articles were screened to identify eligible articles that may have been missed at the initial search. Additionally, reference list screening and citation tracking in Google Scholar were performed for each eligible article.

### Screening

**Figure 1.**
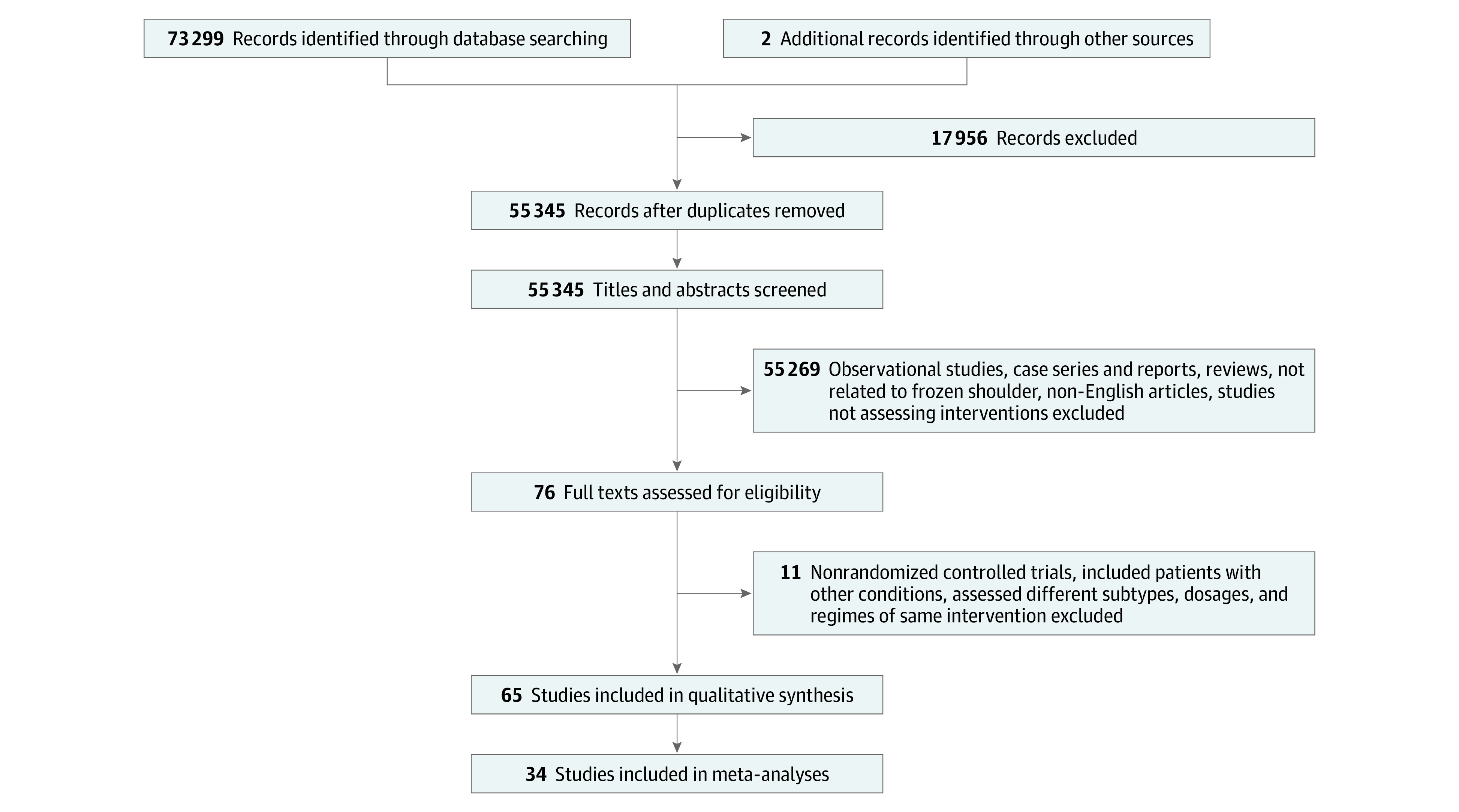
Flow Diagram Summarizing the Article Selection Process

From a total of 73 299 articles that were initially identified, after exclusion of duplicate and noneligible articles, title and abstract screening, and the addition of missed studies identified subsequently, 65 studies were found to fulfil the eligibility criteria. Figure 1 illustrates the article screening process.

### Risk-of-Bias Assessment and Grading the Certainty of Evidence

The internal validity (freedom from bias) of each included study was assessed with the Cochrane Collaboration’s tool for assessing risk of bias in randomized trials separately by 2 of us (D.C. and M.B.), and a third independent opinion (M.M.) was sought when disagreements existed.^15^ Studies were characterized as having low, high, or unclear overall risk of bias based on the following formula: low overall risk studies had high risk of bias in 2 or fewer domains; high overall risk studies had high risk of bias in more than 2 domains; unclear overall risk studies had unclear risk of bias in more than 2 domains, unless they also had high risk of bias in more than 2 domains, in which case they were labeled as high overall risk. Risk of bias was assessed separately for outcome measures that included patient reporting (pain, function) and those that did not (ROM); all studies with nonmasked participants were labeled as high risk in the masking of outcome measures domain for patient-reported outcomes given that the assessors were the participants themselves.

Certainty of evidence was graded with the Grading of Recommendations, Assessment, Development and Evaluations (GRADE) tool (eTable 1 in the Supplement).^16^ The scale starts with high, and depending on how many of the 5 possible limitations used in the GRADE tool were present in each comparison, the study could be downgraded to moderate, low, and very low. Grading of evidence was performed by 2 authors (D.C. and M.B.) independently and any disagreements were resolved by discussion and involvement of a third assessor (M.M.). Each outcome measure within each comparison had its own evidence grade. Our recommendations for clinical practice were based on results of either high or moderate quality evidence with both clinical and statistical significance.

### Data Extraction

Two of us (D.C. and M.B.) performed data extraction. The key characteristics of each eligible article were extracted and inserted in tables in Microsoft Word version 16.43 (Microsoft Corp) to facilitate analysis and presentation. For missing data, attempts were made to contact the original investigators for included studies published less than 10 years ago.

For the presentation of results, outcomes were divided into short-term (≤12 weeks), mid-term (>2 weeks to ≤12 months), and long-term (>12 months) follow-up. When sufficient data existed, short-term follow-up was subdivided into early short-term (2-6 weeks) and late short-term (8-12 weeks). All short-term follow-up points were converted to weeks, and all mid-term follow-up points to months for consistency and easier analysis.

Comparisons of interventions reported by fewer than 3 studies were included in the supplementary results table and were not analyzed or included in the article. When 3 or more studies contributed data for outcome measures at similar follow up times (ie, 2-6 weeks, 8-12 weeks, and 4-6 months), pairwise meta-analyses were conducted. Raw mean differences (MDs) with their accompanying 95% CIs were calculated and used in the tests for each comparison of pain and ER ROM because the tools used across studies were the same. Standardized mean differences (SMDs) were used for function because different functional scores were used.

When pain results were reported in different settings (eg, at rest, at night, with activity) in studies, only pain at rest was used in results. When both active and passive ROM were used as outcome measures, passive ROM was used in our results to increase homogeneity given that most studies used passive ROM. Results for the following outcome measures were recorded in tables and combined qualitatively only based on direction of effect to yield an overall effect for each comparison: abduction ROM, flexion ROM, and quality of life. However, these were not included in the results nor was the quality of the relevant evidence graded.

Additionally, comparisons that yielded both clinically and statistically significant results (ie, greater than or equal to the minimal clinically relevant difference and *P* < .05) underwent trial sequential analysis (TSA) to rule out a type I error and further reinforce our recommendations for clinical practice. TSA is a quantitative method applying sequential monitoring boundaries to cumulative meta-analyses in a similar fashion as the application of group sequential monitoring boundaries in single trials to decide whether they could be terminated early because of a sufficiently small *P* value. TSA is considered an interim meta-analysis; it helps control for type I and II errors and clarifies whether additional trials are needed by considering required information size.^17^ The TSA graph includes 2 horizontal lines, representing the conventional thresholds for statistical significance (*Z* = 1.96; *P* < .05); 1 vertical line, representing required information size; a curved red line, representing the TSA boundaries (ie, thresholds for statistical significance); and a blue line showing the cumulative amount of information as trials are added. A significant result is denoted by a crossing of the curved blue and red lines.

Finally, a network meta-analysis was conducted for treatments used by 3 or more studies for the primary outcome (pain) at late short-term (8-12 weeks) and mid-term (4-6 months) follow-up. Both direct and indirect comparisons were included in the model, and treatment rank probabilities were produced for the 2 follow-up time periods. The certainty of evidence deriving from network meta-analyses was not graded. Subgroup analyses for the effect of home exercise, different physiotherapy interventions, and chronicity of frozen shoulder were conducted when possible.

### Definitions

The term *physiotherapy* was used for any supervised, physiotherapist-led, noninvasive treatment (mobilizations, application of ice and heat, diathermy, electrotherapy modalities). These were grouped and analyzed together. Exercises and stretching that were performed by the participants at home (home exercise program) or under a physiotherapist’s supervision were not included in physiotherapy. Acupuncture and extracorporeal shock wave therapy (ESWT) were regarded as a separate intervention to physiotherapy. Interventions that had accompanying physiotherapy were grouped and analyzed separately from those that did not, regardless of intensity and frequency. For example, studies with a treatment group who received IA corticosteroid plus physiotherapy (eg, ice packs and diathermy) were included in the intervention category IA corticosteroid plus physiotherapy; those with a treatment group receiving only IA corticosteroid (with or without a home exercise program) were included in the IA corticosteroid category. Patients in the following groups were considered control groups and were analyzed together: no treatment, placebo, sham procedures, IA normal saline or lidocaine, simple analgesia, and home exercise alone.

The following tools and questionnaires that were found in included studies represented our function outcome measure: Shoulder Pain and Disability Index, American Shoulder and Elbow Surgeons shoulder score, Constant-Murley, and the Strengths and Difficulties Questionnaire. All patient-reported pain and function scales were uniformly converted to a scale from 0 to 10 and a scale from 0 to 100, respectively.

### Statistical Analysis

The Review Manager version 5 (RevMan) software was used for pairwise meta-analyses and their accompanying forest plots and heterogeneity tests (χ^2^ and *I*^2^). TSA software version 0.9β (Copenhagen Trial Unit) was used for TSAs; random-effect models with 5% type I error and 20% power and O’Brien-Fleming α-spending function were used for all TSA analyses. The required information size was estimated by the software based on the power (20%), mean difference, variance, and heterogeneity. Stata version 16.1 (StataCorp) with the mvmeta extension (multivariate random-effects meta-regression) was used for network meta-analyses (frequentist approach).^18^

When exact mean and SD values were not reported in the included articles, approximate values (to the nearest decimal place) were derived from the graphs. When only interquartile ranges (IQRs) were reported, the SD was calculated as IQR divided by 1.35. When only the median was reported, the mean was assumed to be the same. When CIs of means were reported, SDs were calculated by dividing the length of the CI by 3.92 and then multiplying by the square root of the sample size. When SEs of mean were given, these were converted to SDs by multiplying them by the square root of the sample size. In studies in which only means and the population were given, the SD was imputed using the SDs of other similar studies using the prognostic method (ie, calculating the mean of all SDs).^19^ Pooled means were calculated by adding all the means, multiplied by their sample size, and then dividing this by the sum of all sample sizes. Pooled SDs were calculated with the following formula: SD_pooled_ = √(SD_1_^2^[*n*_1_-1]) + (SD_2_^2^[*n*_2_-1]) + … + (SD*_k_*^2^[*n_k_*-1]) / (*n*_1_ + *n*_2_ + … + *n_k_* – *k*), where *n* indicates sample size and *k*, the number of samples. The following formula was used for the sample size calculation as part of GRADE’s assessment for imprecision^20^:
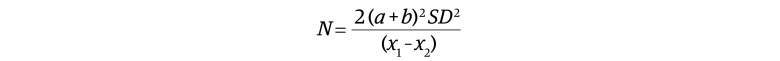
In which *N* indicates the sample size required in each of the groups; (*x_1_* – *x_2_*) indicates the minimal clinically relevant difference (MCRD), defined as 1 point for VAS pain, effect size of 0.45 for functional scores, and 10° for ER ROM; SD^2^ indicates the population variance, calculated using pooled SD from our treatment groups; *a* = 1.96, for 5% type I error; and *b* = 0.842, for 80% power.

The MCRD for function on functional scales would have been set at 10 points. However, because SMDs were used, which produce effect sizes, rather than MDs, the 10 points were divided by the population SD (ie, 22) that was used to calculate the optimal information size (effect sizes can be converted back to functional scores when multiplied by SD).

Potential publication bias was evaluated by Egger test for asymmetry of the funnel plot in comparisons including more than 10 studies. Expecting wide-range variability in studies’ settings, a random-effects metasynthesis was employed in all comparisons.

Subgroup analyses were conducted with independent samples *t* tests in Graphpad version 8 (Prism) comparing pooled means and SDs. All statistical significance levels were set at *P* < .05, tests were 2-tailed, and clinical significance was defined as a MD or SMD being equal or higher than our predefined MCRD.

## Results

Of the 65 eligible studies, a total of 34 studies^21,22,23,24,25,26,27,28,29,30,31,32,33,34,35,36,37,38,39,40,41,42,43,44,45,46,47,48,49,50,51,52,53,54^ were included in pairwise meta-analyses with a total of 2402 participants with frozen shoulder. Duration of symptoms ranged from 1 month to 7 years and length of follow-up from 1 week to 2 years, with most follow-up occurring at 6 weeks, 12 weeks, and 6 months.

Table 1 summarizes the main characteristics of the included studies.^21,22,23,24,25,26,27,28,29,30,31,32,33,34,35,36,37,38,39,40,41,42,43,44,45,46,47,48,49,50,51,52,53,54,55,56,57,58,59,60,61,62,63,64,65,66,67,68,69,70,71,72,73,74,75,76,77,78,79,80,81,82,83,84,85,86,87^ eTable 2 in the Supplement shows the results of the risk-of-bias assessment.

**Table 1.** Main Characteristics of Populations, Interventions, and Outcome Measures of Included Randomized Trials

Source	Participants, No. (participants who completed study, No.)	Mean age, y	Duration of symptoms	Participants per treatment group, No. (participants per treatment group who completed study, No.)	Treatment duration (follow-up)	Outcome measures
Arslan and Celiker,^21^ 2001^a^	20 (20)	56	Mean, 4.1 mo	IA corticosteroid; n = 10 (10) Physiotherapy (hot pack, ultrasound, exercises) with NSAID; n = 10 (10) Both groups received 12-wk home exercise program	Single IA corticosteroid injection; physiotherapy not stated for how long, likely 12 wk (0, 2, and 12 wk)	AROM (ABD, FL, ER, and IR) PROM (ABD, FL, ER, and IR) Pain (VAS, 0-10), unspecified
Bal et al,^22^ 2008^a^	80 individuals with 82 shoulders (64)	56.6	Range, 6 wk-6 mo	IA corticosteroid; n = 40 (40) Sham injection (normal saline); n = 40 (24) Both groups received 12-wk home exercise program	Single IA corticosteroid or normal saline injection (0, 2, and 12 wk)	PROM (ABD, FL, ER, and IR) Night pain (VAS, 0-100) Functional disability (SPADI) Treatment effectiveness (UCLA end-result score)
Binder et al,^55^ 1986	40 (unknown)	54.8	Mean (range), 5.5 mo (1-12 mo)	Oral corticosteroid; n = 20 (unknown) No treatment; n = 20 (unknown) Both groups received home exercise program, which entailed 2-3 min of movement every hour	Oral prednisolone for 6 wk (0, 2, 4, and 6 wk then monthly to 8 mo)	Pain (VAS, 0-10), at rest, at night, on movement PROM (ABD, FL, and ER)
Blockey et al,^56^ 1954	32 (30)	55	Mean, 5.6 mo	Oral corticosteroid; n = 16 (14) Oral placebo; n = 16 (16) Both groups received home exercises for 4 wk; patients who still had restricted ROM at 4 wk underwent MUA and further 4 wk treatment with oral corticosteroid or placebo, according to initial treatment allocation	Oral corticosteroid or placebo for 4 weeks (0, 1, 4, 5, 8, and 18 wk)	Pain (0-3) at rest and on movement” ROM (total ABD, scapulohumeral ABD, total rotation), unclear whether active or passive
Buchbinder et al,^57^ 2004	50 (46)	54.3	Mean, 23.3 wk	Oral corticosteroid; n = 24 (24) Oral placebo; n = 26 (22) Both groups received home exercise program of an unknown duration	Oral corticosteroid or placebo for 3 weeks (0, 3, 6, and 12 wk)	Pain (VAS, 0-10) at night and activity-related Functional disability (SPADI, Croft, DASH) Function (HAQ) QoL (SF-36) Patient-rated improvement AROM (ABD, FL, ER, and IR)
Buchbinder et al,^58^ 2004	46 (46)	57.3	Mean (range), 116 d (96-402 d)	Arthrographic distension with IA corticosteroid; n = 25 (25) Arthrography only (placebo); n = 21 (21) Both groups received home exercise program of an unknown duration	Single injection (0, 3, 6, and 12 wk)	Functional Disability (SPADI and PET) Pain (SPADI and VAS, 0-10), overall (unspecified) AROM (ABD, FL, ER, and IR)
Bulgen at al, ^23^ 1984^a^	45 (42)	55.8	Mean (range), 4.8 mo (1-12 mo)	IA corticosteroid; n = 11 Mobilizations; n = 11 Ice with PNF; n = 12 No treatment; n = 8 All groups received home exercise program of unknown duration	IA corticosteroid once weekly for 6 wk; mobilizations, ice. and PNF three times weekly for 6 wk (0, 1, 2, 3, 4, 5, 6, 7, 8, and 12 wk, 4, 5, and 6 mo)	Pain (VAS, 0-10) at rest, at night, on movement PROM (ABD, FL, and ER)
Calis et al,^24^ 2006^a^	95 shoulders (unknown)	56.9	>1 mo	IA sodium hyaluronate; n = 27 (unknown) IA corticosteroid; n = 26 (unknown) Physiotherapy (hot pack, US, TENS, stretching); n = 22 (unknown) No treatment; n = 20 (unknown) All groups received home exercise program of unknown duration	IA sodium hyaluronate injection once weekly for 2 wk; single IA corticosteroid injection; physiotherapy for 10 d (0, 15 d, 3 mo)	Pain (VAS, 0-10), unspecified PROM (ABD and ER) Function (CM)
Carette et al,^25^ 2003^a^	93 (77)	55.3	Mean, 21.1 wk, everyone <1 y	IA corticosteroid IA with physiotherapy; n = 22 (20) IA corticosteroid; n = 25 (16) IA placebo with physiotherapy; n = 27 (23) IA placebo n = 23 (22) All groups received 3-mo home exercise program; physiotherapy included TENS, mobilizations, exercises, and ice for patients with acute disease and US, mobilizations, exercise, and ice for those with chronic disease	Single injections of IA corticosteroid and placebo; supervised physiotherapy 3 sessions weekly for 4 wk (0, 6 wk, 3 mo, 6 mo, 1 y)	Functional disability (SPADI) Pain (SPADI, 0-100), unspecified QoL (SF-36) AROM and PROM (ABD, FL, and ER)
Cheing et al,^59^ 2008	74 (70)	33-90, unknown mean	Mean (range), 7.2 mo (1-24 mo)	EA; n = 25 (24) IFE; n = 24 (23) No treatment; n = 25 (23) EA and IFE groups received 6-mo home exercise program	10 sessions during 4 wk for EA and IFE (0, 1 mo, 3 mo, 6 mo)	Function (CM) Pain (VAS, 0-10), unspecified
Chen et al,^60^ 2014	40 (34)	53.4	>3 mo	Oral corticosteroid; n = 20 (17) ESWT; n = 20 (17) Both groups received home exercise program of unknown duration	Oral corticosteroid for 4 wk; 3 sessions of ESWT during 4 wk (0, 2, 4, 6, and 12 wk)	Function (CM and OSS) AROM, from CM (ABD, FL, ER, and IR) Pain (CM), unspecified
Cho et al,^26^ 2016^a^	126 (110)	56.6	Mean, 5 mo	IA corticosteroid; n = 42 (36) SA corticosteroid; n = 42 (37) IA with SA corticosteroid, n = 42 (37) All groups received home exercise program of unknown duration	Single injections (0, 3, 6, and 12 wk)	Function (ASES shoulder score) Pain (VAS. 0-10) with movement PROM (ABD, FL, ER, and IR)
Dacre et al,^27^ 1989^a^	66 (62)	54.9	>4 wk	IA corticosteroid; n = 22 (22) Physiotherapy (mobilizations); n = 22 (20) IA corticosteroid with physiotherapy; n = 22 (20) No home exercise program	Supervised physiotherapy for 4-6 wk (0, 6 wk, 6 mo)	Pain (VAS. 0-10) day, night, and with movement PROM (ABD, ER, and IR)
Dahan et al,^61^ 1999	34 (27)	52	Mean, 1 y	Suprascapular nerve block with bupivacaine; n = 17 (15) Placebo injection; n = 17 (12) Both groups received home exercise program of unknown duration	3 injections over 2 weeks	Pain (VAS, 0-10, MPQ short-form, Present Pain Index) Function (SST) AROM (ABD and FL) PROM (ABD, FL and ER)
De Carli et al,^62^ 2012	46 (44)	55.5	Mean, 3 mo	MUA with ACR; n = 25 (23) IA corticosteroid; n = 21 (21) IA corticosteroid group received both supervised physiotherapy and home exercise program; MUA with ACR group started active strengthening 5 wk postoperation	Single MUA with ACR; IA corticosteroid once weekly for 3 wk (0, 3, 6 , and 12 wk, 6 and 12 mo)	Function (CM, UCLA, ASES, and SST) PROM (ABD, FL, ER, and IR) Treatment satisfaction (VAS)
Dehghan et al,^28^ 2013^a^	75 (59); patients had diabetes	54	Not stated	NSAID (naproxen, 1g/d); n = 35 (28) IA corticosteroid; n = 40 (29) Both groups received home exercise program of unknown duration	Single injection of IA corticosteroid; NSAID of unknown duration (0, 2, 6, 12, and 24 wk)	Pain (VAS, 0-10), unspecified ROM (ABD, FL, ER, and IR), unknown if active or passive
Gallacher et al,^63^ 2018	50 (39)	53.9	>3 mo	Arthrographic distension with IA corticosteroid; n = 25 (20) ACR with IA corticosteroid; n = 25 (19) Both groups received home exercises and what authors described as standard physiotherapy regimen of unknown duration	Single treatment (0, 6 wk, 3 mo, 6 mo)	Function (OSS) QoL (EQ-5D) PROM (ABD, FL, and ER) Complications
Gam et al,^29^ 1998^a^	22 (20)	53	Median, 5 mo	IA corticosteroid; n = 9 (8) IA corticosteroid with arthrographic distension; n = 13 (12) No home exercise program	1 injection weekly for 6 wk or until no symptoms (0, 3, 6, and 12 wk)	Pain (VAS, 0-10), at rest and with movement PROM (FL, EXT, ABD, ELE, and ER) Use of analgesics
Hsieh et al,^64^ 2012	70 (63)	54.5	Mean, 4.5 mo	IA sodium hyaluronate with physiotherapy (heat, electrotherapy, exercises); n = 35 (32) Physiotherapy; n = 35 (31) No home exercise program	Injection weekly for 3 wk; physiotherapy for 3 mo (0, 6, and 12 wk)	AROM and PROM (FL, ABD, ER, and IR) Pain (VAS, 0-100), unspecified Functional disability (SPADI and SDQ) QoL (SF-36)
Jacobs et al,^65^ 2009	53 (51)	57	Median, 17.5 mo	MUA; n = 28 (26) IA corticosteroid with arthrographic distension; n = 25 (25) Both groups received home exercise program of unknown duration	Single MUA; 3 IA corticosteroid injections over 18 wk (0, 2, 6, and 12 wk, 6, 9 , 12, 18, and 24 mo)	Function (CM) Pain (VAS, 0-100), unspecified QoL (SF-36)
Jacobs et al,^66^ 1991	47 individuals with 50 shoulders (35)	53.4	Median (range), 6 mo (1-24 mo)	Arthrographic distension; n = 14 (unknown) IA corticosteroid; n = 15 (unknown) Arthrographic distension with IA corticosteroid; n = 18 (unknown) All groups received home exercise program of unknown duration	As many as 3 injections over 12 wk (0, 6, 12, 16)	AROM (ABD, FL, and ER) PROM (ABD, FL, and ER) Strength (dynamometry) Pain with daily activities (0-5) and with movement (0-3) Use of analgesics
Jones and Chattopadhyay,^67^ 1999	30 (30)	56.5	Not stated	Suprascapular nerve block; n = 15 (15) IA corticosteroid; n = 15 (15) Both groups received home exercise program of unknown duration	Single suprascapular nerve block; ≤3 IA corticosteroid injections (0, 1, 3, 7, and 12 wk)	Pain (VAS. 0-5), unspecified ROM (ABD, ER, and IR), unknown if active or passive
Khallaf et al,^30^ 2018^a^	40 (unknown)	47.3	Mean, 1.5 mo	IA corticosteroid; n = 20 (unknown) SA corticosteroid; n = 20 (unknown) Both groups received 12-wk home exercise program	Single injection (0 and 12 wk)	Pain (VAS, 0-10), unspecified Functional disability (SPADI) AROM (FF, ER, IR, and EXT) PROM (FF, ER, IR, and EXT)
Khan et al,^68^ 2005	36 (35)	Unknown	Not stated	Physiotherapy (exercises, TENS, and IRR); n = 18 (unknown) Physiotherapy with arthrographic distension and IA corticosteroid; n = 18 (unknown)	8 wk (0, 1, 2, 3, 4, 5, 6, 7, and 8 wk)	Pain (VAS, 0-100), unspecified PROM (ABD, ER, and IR)
Kim et al,^69^ 2017	40 (30)	55.2	Mean, 4 mo	IA sodium hyaluronate; n = 20 (16) IA sodium hyaluronate with IA tramadol; n = 20 (14) Both received home exercise program of unknown duration	IA sodium hyaluronate weekly injections for 5 wk; IA tramadol for 3 wk (0, 1, 2, 3, 4, and 6 wk)	Pain (VAS 0-10), unspecified Functional disability (SPADI) PROM (ABD, FL, ER, and IR)
Kivimäki and Pohjolainen,^70^ 2001	30 (24)	51	Mean (range), 7 mo (3-18 mo)	MUA with IA corticosteroid n = 15 (13) MUA; n = 15 (11) No home exercise program	Single treatment (0, 1 d, 4 mo)	PROM (ABD, FL, ER, and IR)
Kivimäki et al,^71^ 2007	125 (83)	53	Mean, 7.2 mo	MUA; n = 65 (38) No treatment; n = 60 (45) Both groups received home exercise program of unknown duration	Single MUA (0, 6 wk, 3, 6, and 12 mo)	PROM (ABD, FL, ER, and IR) Pain (VAS, 0-10), unspecified Functional disability (modified SDQ) Function (working ability, 0-10) Use of analgesics
Klç et al,^72^ 2015	41 (41)	58.4	>1 mo	Suprascaular nerve block with physiotherapy; n = 19 (19) Physiotherapy; n = 22 (22) Physiotherapy included hot packs, exercises, stretching, TENS, and US; both groups received home exercise program of unknown duration	Physiotherapy, 5 sessions a week for 3 weeks; single suprascapular nerve block (0, 3, and 7 wk)	Pain (BPI-SF) Function (CM)
Koh et al,^31^ 2013^a^	68 (unknown)	54.4	Mean, 6 mo	Bee venom acupuncture with physiotherapy; n = 22 (unknown) Higher dose bee venom acupuncture with physiotherapy; n = 23 (unknown) Sham injection (normal saline) with physiotherapy; n = 23 (unknown) Physiotherapy included TENS, TDP, and mobilizations; all groups received 2-mohome exercise program	16 sessions during 2 mo (0, 2, 4, 8, and 12 wk)	Disability (SPADI) Pain (VAS, 0-10), at rest, at night, and with movement AROM (FL, EXT, ABD, ADD, and ER) PROM (FL, EXT, ABD, ADD, and ER)
Kraal et al,^32^ 2018^a^	21 (15)	51.9	>3 mo	IA corticosteroid with physiotherapy (mobilizations, stretching, ice and hot packs, and massage); n = 10 (unknown) IA corticosteroid; n = 11 (unknown) No home exercise program	Single injection but second given if no improvement at 6 wk; physiotherapy twice weekly ≤3 mo (0, 6, 12, and 26 wk)	Functional disability (SPADI) Pain (NPRS, 0-10), mean and at night QoL (SF-36) PROM (ABD and ER) Patient satisfaction (0-5)
Lee et al,^33^ 1974^a^	65 (unknown)	57.3	Between 3 mo and 5 y	Physiotherapy (heat and exercises); n = 17 (unknown) Analgesics; n = 15 (unknown) IA corticosteroid with physiotherapy (heat and exercises) n = 15 (unknown) Bicep tendon corticosteroid with physiotherapy (heat and exercises) n = 18 (unknown)	Details regarding number of injections and duration of physiotherapy and analgesics not given (0, 1, 2, 3, 4, 5, and 6 wk)	AROM (ABD) PROM (ABD, ER, and IR)
Lee et al,^34^ 2017^a^	64 (64)	54.9	Mean, 8 mo	IA corticosteroid; n = 32 (32) IA corticosteroid with arthrographic distension; n = 32 (32) Both groups received 6-wk home exercise program	Single injection (0, 3, 6, and 12 wk)	Pain (VAS, 0-10), global Functional disability (SPADI) PROM (ABD, FL, EXT, ER, and IR)
Lee et al,^35^ 2017^a^	30 (unknown)	58.7	Not stated	ESWT with physiotherapy (hot packs, US, and electrotherapy); n = 15 (unknown) Physiotherapy (hot packs, US, and electrotherapy); n = 15 (unknown) No home exercise program	Both treatments 3 times weekly for 4 wk (0 and 4 wk)	Pain (VAS, 0-10), unspecified ROM (FL, ER), unknown if active or passive
Lim et al,^73^ 2014	68 (62)	53.8	Mean, 7.3 mo	IA corticosteroid; n = 34 (33) IA sodium hyaluronate; n = 34 (29) Both groups received home exercise program	Single injection of IA corticosteroid; 3 injections sodium hyaluronate (0, 2, and 12 wk)	Pain (VAS, 0-10), unspecified Function (ASES and CM) AROM (FL, ER, and IR)
Lo et al,^36^ 2020^a^	21 (unknown)	59.6	Everyone >3 mo	Electroacupuncture with physiotherapy; n = 11 (unknown) Sham electroacupuncture with physiotherapy; n = 10 (unknown) Physiotherapy included hot packs, exercises, and ice packs	18 sessions during 6-9 wk (0, 1, 3, and 6 mo)	Pain (VAS, 0-10), with movement AROM (FL, EXT, ABD, ADD, ER, and IR) PROM (FL, EXT, ABD, ADD, ER, and IR) Functional disability (SPADI)
Lorbach et al,^74^ 2010	40 (unknown)	51	Mean, 11 mo	IA corticosteroid; n = 20 (unknown) Oral corticosteroid; n = 20 (unknown) Both groups received supervised physiotherapy (unspecified) and 8-wk home exercise program	IA corticosteroid 3 injections during 8 wk; oral corticosteroid for 25 d (0, 4, 8, and 12, 6 and 12 mo)	Function (CM, SST, and VAS) Pain (VAS, 0-10, reversed), unspecified PROM (FL, ER, and IR) Patient satisfaction (VAS)
Ma et al,^37^ 2006^a^	75 (unknown)	54.8	Mean, 25.8 wk, everyone >3 mo	Physiotherapy; n = 30 (unknown) Acupuncture; n = 30 (unknown) Physiotherapy with acupuncture; n = 15 (unknown) Physiotherapy included hot pack, mobilizations, and exercises; no home exercise program	Acupuncture twice weekly for 4 wk; physiotherapy 5 times weekly for 4 wk (0, 2, and 4 wk)	Pain (VAS, 0-10), at rest and with movement AROM (ABD, FL, EXT, ER, and IR) PROM (ABD, FL, EXT, ER, and IR) QoL (SF-36)
Maryam et al,^38^ 2012^a^	87 (69)	53.6	Mean, 5.8 mo, everyone <1 y	Physiotherapy; n = 27 (8) IA corticosteroid with physiotherapy; n = 29 (14) IA corticosteroid; n = 31 (14) Physiotherapy included TENS, exercises, and ice; all groups received home exercise program of unknown duration	Single injection of IA corticosteroid single injection; 10 sessions of physiotherapy (0 and 6 wk)	Functional disability (SPADI) Pain (SPADI, 0-100), unspecified AROM (ABD, FL, and ER) PROM (ABD, FL, and ER)
Mukherjee et al,^75^ 2017	60 (56)	50.4	Mean, 6.3 mo	ACR; n = 30 (28) IA corticosteroid; n = 30 (28) Both groups received home exercise program of unknown duration	Single treatment (0, 4, 8, 12, 16, and 20 wk)	Pain (VAS, 0-10), unspecified PROM (ABD, EXT, ER, and IR) Function (CM)
Mun and Baek,^76^ 2016	136 (121)	53	Mean, 6.5 mo, everyone >3 mo	Arthrographic distension with IA corticosteroid and MUA; n = 67 (60) IA corticosteroid; n = 69 (61) Both groups received supervised exercises for 1 mo followed by home exercise program of unknown duration	Single injection (0, 2, 6, 12, 24, and 48 wk)	Pain (VAS, 0-10), unspecified Function (CM) Satisfaction (VAS) PROM (FL, ER, and IR)
Oh et al,^39^ 2011^a^	71 (58)	57	Mean, 6.6 mo	IA corticosteroid; n = 37 (31) SA corticosteroid; n = 34 (27) Both groups received home exercise program of unknown duration	Single injection (0, 3, 6, and 12 wk)	Pain (VAS, 0-10), unspecified Function (CM) PROM (ABD, ER, and IR)
Park and Hwnag,^40^ 2000^a^	55	56.5	Not stated	IA corticosteroid with arthrographic distension; n = 28 (unknown) IA corticosteroid; n = 27 (unknown) Unclear whether home exercise program was used	Single injection (0, 1 wk, 1 mo)	Pain (VAS, 0-10) AROM (ABD, FL, ER, and IR) Cyriax stages of arthritis
Park et al,^77^ 2013	90 (90)	55.8	Mean (range), 5.3 mo (3-9 mo)	Arthrographic distension with sodium hyaluronate; n = 45 (45) IA corticosteroid n = 45 (45) Both groups received home exercise program of unknown duration	3 injections during 4 wk (0, 2, and 6 wk)	Functional Disability (SPADI) Pain (VAS and SPADI) PROM (ABD, FL, and ER) Complications
Park et al,^78^ 2014	53 (unknown)	56	Range, 3-9 mo	Arthrographic distension with IA corticosteroid, intensive mobilization, and general physiotherapy; n = 16 (unknown) Arthrographic distension with IA corticosteroid and general physiotherapy; n = 12 (unknown) Intensive mobilization with general physiotherapy; n = 14 (unknown) General physiotherapy; n = 11 (unknown) General physiotherapy included hot packs, TENS, and US; all groups received home exercise program of unknown duration	All treatments twice weekly for 4 wk (0 and 4 wk)	Pain (VAS, 0-10), unspecified Functional disability (SPADI) Function (CM) AROM (ABD, FL, ER, and IR)
Park et al,^41^ 2015	30 (unknown)	53.5	Not stated	ESWT with physiotherapy; n = 15 (unknown) Physiotherapy; n = 15 (unknown) Physiotherapy included hot packs, US, and electrotherapy; no home exercise program	Twice weekly for 6 wk (0 and 4 wk)	Pain (VAS, 0-10), unspecified Function (patient-specific functional scales)
Prestgaard et al,^42^ 2015^a^	122 (114)	54.5	Mean (range), 15 wk (1-6 mo)	IA corticosteroid; n = 42 (39) IA with rotator interval corticosteroid; n = 40 (39) Sham injection (IA with rotator interval local anesthetic); n = 40 (36) No home exercise program	Single injection (0, 3, 6, 12, and 26 wk)	Pain (VAS, 0-10), general and at night Shoulder disability (SPADI) AROM (ABD, FL, and ER) Use of analgesics QoL (EQ-5D)
Pushpasekaran et al,^79^ 2017	85 (80)	56.3	Mean (range), 15.2 mo (2.5-49 mo)	IA corticosteroid; n = 43 (40) 3-site injection (IA, SA, and subcoracoid); n = 42 (40) Both groups received NSAIDs, physiotherapy (US), and 4-wk home exercise program for prior to intervention	2 treatments during 3 wk (0, 3, and 6 wk, 6 mo)	Function (CM)
Quaraishi et al,^80^ 2007	36 individuals with 38 shoulders (33)	55.2	Mean (range), 33.7 wk (12-76 wk)	Arthrographic distension; n = 19 (18) MUA with IA corticosteroid n = 17 (15) Both groups received home exercise program of unknown duration	Single treatment (0, 2 mo, 6 mo)	Pain (VAS, 0-10), unspecified Function (CM) PROM (ABD, FL, ER, and IR) Satisfaction
Ranalletta et al,^43^ 2015^a^	74 (69)	63.4	Mean, 12 wk, everyone >1 mo	IA corticosteroid; n = 36 (34) NSAID; n = 38 (35) Both groups received supervised exercises and 12-wk home exercise program	Single IA corticosteroid injection; NSAID twice a day for unknown duration (0, 2, 4, 8, and 12)	Pain (VAS, 0-10), overall Function (ASES and CM) Functional disability (qDASH) PROM (ABD, FL, EXT, ER, and IR)
Reza et al,^44^ 2013^a^	100 (100)	59.5	Mean, 115 d, everyone >3 mo	IA corticosteroid; n = 50 (50) Arthrographic distention with IA corticosteroid; n = 50 (50) Both groups received home exercise program of unknown duration	Single injection (0, 2 d, 12 wk)	Pain (VAS, 0-10), unspecified ROM (ABD, FL, EXT, ER, and IR) unknown whether active or passive
Rizk et al,^45^ 1991^a^	48 (44)	55	Mean (range), 13.2 wk (8-18 wk)	IA corticosteroid; n = 16 (15) Intrabursal (SA) corticosteroid; n = 16 (14) IA LA; n = 8 (8) Intrabursal (SA) LA; n = 8 (7) All groups received home exercise program of unknown duration	3 injections during 2 wk (weekly 0-11 wk, 15 wk, and 6 mo)	PROM (total ROM) Pain (VAS, 0-5), unspecified
Roh et al,^46^ 2011^a^	50 (45); patients with diabetes	54.9	Mean (range), 6.4 wk (4 wk-6 mo)	IA corticosteroid; n = 25 (23) No treatment; n = 25 (22) Both groups received home exercise program of unknown duration	Single injection (0, 4, 12, and 24 wk)	PROM (FL, ER, and IR) Pain (VAS, 0-10), unspecified Function (ASES)
Rouhani et al,^81^ 2016	72 (64)	52.8	Not stated	Calcitonin nasal spray with physiotherapy (details not stated); n = 36 (32) Placebo spray with physiotherapy (details not stated); n = 36 (32) Both groups received oral NSAIDs	Calcitonin and placebo spray for 6 weeks (0 and 6 wk)	Pain (VAS, 0-10), overall and at night Functional disability (DASH and SPADI) QoL (HAQ) PROM (ABD, FL, and ER)
Ryans et al,^47^ 2005^a^	80 (78)	54.1	Mean, 10.4 wk, everyone >4 wk	IA corticosteroid with physiotherapy; n = 20 (20) IA corticosteroid; n = 20 (19) IA placebo (normal saline) with physiotherapy; n = 20 (20) IA placebo; n = 20 (19) Physiotherapy included PNF, mobilizations, electrotherapy, and exercises; all groups received home exercise program of unknown duration	Single injection; physiotherapy of unknown duration (0, 6, and 16 wk)	AROM (ABD, FL, ER, and IR) PROM (ABD, FL, ER, and IR) Pain (VAS, 0-100), at rest Function (VAS and HAQ) Functional disability (SDQ) QoL (SF-36)
Schröder et al,^82^ 2017	60 (60)	53.5	Mean, 15.6 mo	Acupuncture; n = 30 (30) Sham acupuncture; n = 30 (30) No home exercise program	Single session (baseline and postsession)	Function (CM) Pain (CM, 0-15)
Schydlowsky et al,^83^ 2012	18 (14)	51	Everyone >3 wk	IA adalimumab; n = 10 (6) IA corticosteroid; n = 8 (8) No home exercise program	1 injection every 2 wk to 3 injections (0, 2, 4, 8, 12, and 24 wk)	Function (CM) Functional disability (SPADI) AROM (ABD, FL, and ER) PROM (ABD, FL, and ER) Pain (SRQ)
Sharma et al,^48^ 2016^a^	106 (87)	53	Median (range), 6.8 mo (2-37 mo)	IA corticosteroid; n = 36 (34) IA corticosteroid with arthrographic distension; n = 34 (32) Treatment as usual (physiotherapy, analgesia, or no treatment); n = 36 (21) No home exercise program	4 injections during 1 mo (0, 4 and 8 wk, 12 mo)	Functional disability (SPADI) Pain (NRS, 0-10), mean PROM (ABD, and ER, IR)
Shin and Lee,^49^ 2013^a^	191 (158)	55.7	>3 mo, mean 7.2 mo	SA corticosteroid; n = 49 (41) IA corticosteroid; n = 48 (42) SA with IA corticosteroid; n = 47 (39) NSAID; n = 49 (36) All groups received home exercise program of >3-mo duration	Single SA and IA injection; oral NSAID for 6 wk (0, 2, 4, 8, 16, and 24 wk)	Function (ASES) Pain (VAS, 0-10), unspecified Treatment satisfaction (VAS) AROM (FL, ER, and IR)
Sun et al,^84^ 2001	35 (30)	56.3	Mean, 6.5 mo	No treatment; n = 22 (18) Acupuncture; n = 13 (12) Both groups received supervised exercises for 6 wk and home exercise program of unknown duration	Acupuncture twice weekly for 6 wk (0, 6, 20 wk)	Function (CM)
Sun et al,^50^ 2018^a^	97 (77)	53.9	Mean, 15.2 wk, everyone <9 mo	IA corticosteroid; n = 30 (24) SA corticosteroid; n = 34 (26) Rotator interval corticosteroid; n = 33 (27) All groups received home exercise program of unknown duration	Single injection (0, 4, 8, and 12 wk)	Pain (VAS, 0-10), unspecified Function (CM) Functional disability (DASH) PROM (ABD, FL, ER, and IR)
Tveitå et al,^51^ 2008^a^	76 (69)	51.5	Mean, 7 mo, everyone 3 mo-2 y	Arthrographic distension with IA corticosteroid; n = 39 (36) IA corticosteroid; n = 37 (33) No home exercise program	3 injection during 4 wk (0 and 10 wk)	Functional disability (SPADI) AROM (ABD, FL, ER, and IR) PROM (ABD, FL, ER, and IR)
Vahdatpour et al,^52^ 2014^a^	40 (36)	58.2	Not stated	ESWT; n = 20 (19) Sham ESWT; n = 20 (17) All patients had a single IA corticosteroid injection at time of inclusion in the study and received home exercise program	Once weekly for 4 wk (0, 4 and 12 wk, 6 mo)	Pain (SPADI 0-100), unspecified Functional disability (SPADI) PROM (ABD, FL, EXT, ER, and IR)
van der Windt,^53^ et al 1998^a^	109 (103)	58.5	82 with <6 mo; 27 with >6 mo	Physiotherapy (mobilizations and exercises) n = 56 (54) IA corticosteroid; n = 53 (49) Physiotherapy group received ice and hot packs and electrotherapy at the physiotherapist’s discretion; no home exercise program	Physiotherapy for 6 wk; IA corticosteroid as many as 3 injections during 6 wk (0, 3, 7, 13,26, and 52 wk)	Satisfaction (0-5) Pain (VAS, 0-100), during day and at night Functional disability (SDQ) PROM (ABD and ER)
Widiastuti-Samekto and Sianturi,^85^ 2004	28 (27)	40-69	Range, 1-6 mo	IA corticosteroid with physiotherapy; n = 13 (13) Oral corticosteroid with physiotherapy; n = 15 (14) Physiotherapy was supervised and included 20 sessions of mobilizations and ice and hot packs; no home exercise program	Single IA corticosteroid injection; oral corticosteroid for 3 wk (0, 1, 2, and 3 wk)	Treatment success (90% improvement in ABD and ER PROM) Pain (VAS, 0-10), unspecified
Yoon et al,^54^ 2016^a^	90 (86)	55	Mean, 9 mo	IA corticosteroid; n = 30 (29) SA corticosteroid; n = 30 (29) Arthrographic distension with IA corticosteroid; n = 30 (28) All groups received home exercise program of unknown duration	Single injection (0, 1, 3, and 6 mo)	Pain (VAS, 0-10), unspecified Function (SST and CM) PROM (FL, ER, and IR)

Abbreviations: ABD, abduction; ACR, arthroscopic capsular release; AROM, active range of movement; ASES, American Shoulder and Elbow Surgeons questionnaire; BPI-SF, Brief Pain Inventory–Short Form; CM, Constant-Murley score; DASH, Disabilities of the Arm, Shoulder, and Hand questionnaire; EA, electroacupuncture; ELE, elevation; EQ-5D, Euro-Qol–5 Dimensions questionnaire; ER, external rotation; ESWT, extracorporeal shock wave therapy; EXT, extension; FL, flexion; HAQ, Health Assessment Questionnaire; IA, intra-articular; IFE, interferential electrotherapy; IR, internal rotation; IRR, infrared radiotherapy; LA, local anesthetic; MPQ, McGill Pain Questionnaire; MUA, manipulation under anesthesia; NPRS, numerical pain rating scale; NSAID, nonsteroidal anti-inflammatory drug; OSS, Oxford Shoulder Score; PET, problem elicitation technique; PNF, proprioceptive neuromuscular facilitations; PROM, passive range of movement; qDASH, quick DASH; QoL, quality of life; SA, subacromial; SDQ, Shoulder Disabilities Questionnaire; SF-36, 36-item short-form survey; SPADI, Shoulder Pain and Disability Index; SRQ, self-reporting questionnaire; SST, Simple Shoulder Test; TDP, transcutaneous infrared thermotherapy; TENS, transcutaneous electrical nerve stimulation; UCLA, University of California Los Angeles questionnaire; US, ultrasound; VAS, visual analog scale.

^a^ Studies included in meta-analyses.

Table 2 summarizes the findings of the present review. Where feasible (ie, results at similar follow-up times in at least 3 studies), pairwise meta-analyses were conducted. The results of abduction ROM, flexion ROM, and quality of life were pooled only based on direction of effect, and their certainty of evidence was not graded. eTable 3 in the Supplement summarizes the results of comparisons reported by 1 or 2 studies only. eTable 4 in the Supplement demonstrates how the strength of evidence for each outcome measure within each comparison was derived for all follow-up time categories, per GRADE. eTable 5 in the Supplement shows the heterogeneity for each comparison (*I*^2^ statistic) and where studies were removed to reduce heterogeneity based on sensitivity analyses.

**Table 2.** Results of Pairwise Comparisons of Interventions of the Included Studies

Source	Pain	Function	ROM ER	ROM ABD	ROM FL	Satisfaction or QoL
**Arthrographic distension with IA corticosteroid vs IA corticosteroid only**						
Jacobs et al,^41^ 1991	NA	NA	No change at 4 mo	No change at 4 mo	No change at 4 mo	NA
Gam et al,^29^ 1998	No change at 3, 6, or 12 wk	NA	No change at 3 and 6 wk; increase at 12 wk	No change at 3, 6, or 12 wk	Increase at 3, 6, and 12 wk	NA
Tveitå et al,^51^ 2008	NA	No change at 10 wk	No change at 10 wk	No change at 10 wk	No change at 10 wk	NA
Reza et al,^44^ 2013	Decrease at 12 wk	NA	Increase at 12 wk	Increase at 12 wk	Increase at 12 wk	NA
Sharma et al,^48^ 2016	No change at 4 or 8 wk	No change at 4, 8, or 12 mo	No change at 4 or 8 wk	No change at 4 or 8 wk	NA	NA
Park and Hwnag,^40^ 2000	No change at 1 or 4 wk	NA	No change at 1 or 4 wk	Increase at 1 wk; no change at 4 wk	Increase at 1 and 4 wk	NA
Yoon et al,^54^ 2016	Increase at 4 wk; no change at 12 wk or 6 mo	Increase at 4 wk and 12 wk; no change at 6 mo	Increase at 4 wk; no change at 12 wk or 6 mo	NA	Increase at 4 wk; no change at 12 wk or 6 mo	NA
Lee et al,^34^ 2017	No change at 3, 6, or 12 wk	No change at 3, 6, or 12 wk	No change at 3, 6, or 12 wk	No change at 3, 6, or 12 wk	No change at 3, 6, or 12 wk	NA
Quality of evidence	Decrease at early short-term (high)^a^; decrease at late short-term (high)^a^	No change at early short-term (moderate)^a^; no change at late short-term (high)^a^	No change at early short-term (high)^a^; no change at late short-term (high)^a^	No change at early short-term; no change at late short-term	Increase at early short-term; no change at late short-term	NA
**Physiotherapy vs no treatment or placebo**						
Calis et al,^24^ 2006	No change at 2 or 12 wk	Decrease at 2 and 12 wk	Increase at 2 and 12 wk	Increase at 2 and 12 wk	NA	NA
Carette et al,^25^ 2003	No change at 6 wk, 12 wk, 6 mo, or 12 mo	No change at 6 wk, 12 wk, 6 mo, or 12 mo	No change at 6 wk, 12 wk, 6 mo, or 12 mo	No change at 6 wk, 12 wk, 6 mo, or 12 mo	No change at 6 wk, 6 mo, or 12 mo; increase at 12 wk	No change at 6 wk, 12 wk, 6 mo, or 12 mo
Bulgen et al,^23^ 1986	No change at 6 wk or 6 mo	NA	No change at 6 wk or 6 mo	No change at 6 wk or 6 mo	No change at 6 wk or 6 mo	NA
Lee et al,^33^ 1974	NA	NA	Increase at 1-6 wk	Increase at 1-6 wk	NA	NA
Quality of evidence	No change at early short-term	NA	Increase at early short-term (moderate)^a^^,^^b^	No change at early short-term	NA	NA
**IA corticosteroid vs IA no treatment or placebo**						
Bal et al,^22^ 2008	No change at 2 wk or 12 wk	Increase at 2 wk; no change at 12 wk	No change at 2 wk or 12 wk	Increase at 2 wk; no change at 12 wk	No change at 2 wk or 12 wk	NA
Calis et al,^24^ 2006	No change at 2 wk; decrease at 12 wk	No change at 2 wk; increase at 12 wk	No change at 2 or 12 wk	No change at 2 wk; increase at 12 wk	NA	NA
Carette et al,^25^ 2003	Decrease at 6 and 12 wk; no change at 6 or 12 mo	Increase at 6 and 12 wk; no change at 6 or 12 mo	Increase at 6 and 12 wk; no change at 6 or 12 mo	No change at 6 wk, 6 mo, or 12 mo; increase at 12 wk	No change at 6 wk, 6 mo, or 12 mo; increase at 12 wk	No change at 6 wk, 12 wk, 6 mo, or 12 mo
Bulgen et al,^23^ 1986	No change at 6 wk or 6 mo	NA	Increase at 6 wk; no change at 6 mo	Increase at 6 wk; no change at 6 mo	Increase at 6 wk; no change at 6 mo	NA
Dehghan et al,^28^ 2013	No change at 2, 6, 12, or 24 wk	NA	No change at 2, 6, 12, or 24 wk	No change at 2, 6, 12, or 24 wk	No change at 2, 6, 12, or 24 wk	NA
Ranalletta et al,^43^ 2015	Decrease at 2, 4, and 8 wk; no change at 12 wk	Increase at 2, 4, 8, and 12 wk	Increase at 2 wk; no change at 4, 8, or 12 wk	Increase at 2, 4, 8, and 12 wk	Increase at 2, 4, 8, and 12 wk	NA
Roh et al,^46^ 2011	Decrease at 4 wk; no change at 12 wk or 6 mo	Increase at 12 wk; no change at 4 wk or 6 mo	No change at 4 wk, 12 wk, or 6 mo	NA	No change at 4 wk or 6 mo; increase at 12 wk	NA
Sharma et al,^48^ 2016	Decrease at 4 and 8 wk	Increase at 4 and 8 wk; no change at 12 mo	Increase at 4 and 8 wk	Increase at 4 and 8 wk	NA	NA
Shin and Lee,^49^ 2013	Decrease at 2, 4, and 8 wk and 4 mo; no change at 6 mo	Increase at 2, 4, and 8 wk and 4 mo; no change at 6 mo	Increase at 2, 4, and 8 wk and 4 mo; no change at 6 mo	NA	Increase at 2, 4, and 8 wk and 4 mo; no change at 6 mo	NA
Rizk et al,^45^ 1991	No change at 1-11 wk and 4 and 6 mo	NA	No change at 11 wk or 6 mo	No change at 11 wk or 6 mo	No change at 11 wk or 6 mo	NA
Ryans et al,^47^ 2005	No change at 6 wk or 4 mo	No change at 6 wk or 4 mo	No change at 6 wk or 4 mo	No change at 6 wk or 4 mo	NA	NA
Prestgaard et al,^42^ 2015	Decrease at 6 and 12 wk; no change at 3 wk or 6 mo	Increase at 3, 6, and 12 wk; no change at 6 mo	Increase at 6 and 12 wk; no change at 3 wk or 6 mo	No change at 3, 6, or 12 wk or 6 mo	Increase at 6 and 12 wk; no change at 3 wk or 6 mo	Increase at 6 and 12 wk; no change at 3 wk or 6 mo
Quality of evidence	Decrease at early short-term (high)^a^^,^^b^; decrease at late short-term (moderate)^a^^,^^b^; no change at mid-term (moderate)^a^	Increase at early short-term (moderate)^a^^,^^b^; increase at late short-term (moderate)^a^^,^^b^; increase at mid-term (moderate)^a^	Increase at early short-term (high)^a^; increase at late short-term (high)^a^; no change at mid-term (moderate)^a^	No change at early short-term; increase at late short-term; no change at mid-term	Increase at early short-term; increase at late short-term; no change at mid-term	No change at early short-term; no change at mid-term
**IA corticosteroid with physiotherapy vs no treatment or placebo**						
Ryans et al,^47^ 2005	No change at 6 wk or 4 mo	Increase at 6 wk; no change at 4 mo	No change at 6 wk or 4 mo	NA	NA	NA
Carette et al,^25^ 2003	Decrease at 6 and 12 wk; no change at 6 or 12 mo	Increase at 6 and 12 wk; no change at 6 or 12 mo	Increase at 6 wk, 12 wk, and 6 mo; no change at 12 mo	Increase at 6 wk, 12 wk, and 6 mo; no change at 12 mo	Increase at 6 wk, 12 wk, and 6 mo; no change at 12 mo	No change at 6 wk, 12 wk, 6 mo, or 12 mo
Lee et al,^33^ 1974	NA	NA	Increase at 1-6 wk	Increase at 1-6 wk	NA	NA
Quality of evidence	NA	NA	Increase at early short-term (high)^a^^,^^b^	NA	NA	NA
**IA corticosteroid vs physiotherapy**						
Arslan and Celiker,^21^ 2001	No change at 2 or 12 wk	NA	No change at 2 or 12 wk	No change at 2 or 12 wk	No change at 2 or 12 wk	NA
Bulgen at al,^23^ 1984	No change at 6 wk or 6 mo	NA	Increase at 6 wk; no change at 6 mo	Increase at 6 wk; no change at 6 mo	Increase at 6 wk; no change at 6 mo	NA
Carette et al,^25^ 2003	Decrease at 6 wk; no change at 12 wk, 6 mo, or 12 mo	Increase at 6 wk; No change at 12 wk, 6 mo, or 12 mo	No change at 6 wk, 12 wk, 6 mo, or 12 mo	No change at 6 wk, 12 wk, 6 mo, or 12 mo	No change at 6 wk, 12 wk, 6 mo, or 12 mo	No change at 6 wk, 12 wk, 6 mo, or 12 mo
Calis et al,^24^ 2006	No change at 2 or 12 wk	No change at 2 or 12 wk	Decrease at 2 or 12 wk	No change at 2 or 12 wk	NA	NA
van der Windt et al,^53^ 1998	Decrease at 3, 7, and 13 wk and 6 and 12 mo	Increase at 3, 7, and 13 wk and 6 and 12 mo	Increase at 3 wk, 7 wk, and 6 mo	No change at 3 wk, 7 wk, and 6 mo	NA	NA
Dacre et al,^27^ 1989	No change at 6 wk or 6 mo	NA	No change at 6 wk or 6 mo	No change at 6 wk or 6 mo	NA	NA
Maryam et al,^38^ 2012	No change at 6 wk or 6 mo	No change at 6 wk or 6 mo	No change at 6 wk or 6 mo	No change at 6 wk or 6 mo	No change at 6 wk or 6 mo	NA
Ryans et al,^47^ 2005; with home exercise	No change at 6 wk or 4 mo	No change at 6 wk or 4 mo	No change at 6 wk or 4 mo	NA	NA	NA
Quality of evidence	No change at early short-term (moderate)^a^; decrease at late short-term (high)^a^^,^^b^; no change at mid-term (low)	Increase at early short-term (moderate)^a^^,^^b^; no change at late short-term (moderate)^a^; increase at mid-term (moderate)^a^	No change at early short-term (moderate)^a^; no change at late short-term (high)^a^; Increase at mid-term (moderate)^a^	No change at early short-term; no change at late short-term; no change at mid-term	No change at early short-term; no change at late short-term; no change at mid-term	NA
**IA corticosteroid with physiotherapy vs IA corticosteroid only**						
Kraal et al,^32^ 2018	No change at 6 wk, 12 wk, or 6 mo	Increase at 6 wk; no change at 12 wk or 6 mo	Increase at 6 wk and 12 wk; no change at 6 mo	Increase at 6 wk and 12 wk; no change at 6 mo	Increase at 6 wk; no change at 12 wk or 6 mo	No change at 6 wk, 12 wk, or 6 mo
Dacre et al,^27^ 1989	No change at 6 wk or 6 mo	NA	No change at 6 wk or 6 mo	No change at 6 wk or 6 mo	NA	NA
Maryam et al,^38^ 2012	No change at 6 wk or 6 mo	No change at 6 wk or 6 mo	No change at 6 wk or 6 mo	No change at 6 wk or 6 mo	No change at 6 wk or 6 mo	NA
Carette et al,^25^ 2003	No change at 6 wk, 12 wk, 6 mo, or 12 mo	No change at 6 wk, 12 wk, 6 mo, or 12 mo	No change at 6 wk, 12 wk, 6 mo, or 12 mo	No change at 6 wk, 12 wk, 6 mo, or 12 mo	Increase at 6 wk, 12 wk, and 6 mo; no change at 12 mo	No change at 6 wk, 12 wk, 6 mo, or 12 mo
Ryans et al,^47^ 2005	No change at 6 wk or 4 mo	No change at 6 wk or 4 mo	No change at 6 wk or 4 mo	NA	NA	NAs
Quality of evidence	No change at early short-term (moderate)^a^; no change at mid-term (moderate)^a^	No change at early short-term (low)^a^; no change at mid-term (high)^a^	Increase at early short-term (moderate)^a^^,^^b^; no change at mid-term (high)^a^	No change at early short-term; no change at mid-term	Increase at early short-term; no change at mid-term	No change at early short-term; no change at late short-term; no change at mid-term
**IA corticosteroid with physiotherapy vs physiotherapy only**						
Carette et al,^25^ 2003	Decrease at 6 wk; no change at 12 wk, 6 mo, or 12 mo	Increase at 6 wk; no change at 12 wk, 6 mo, or 12 mo	Increase at 6 wk, 12 wk, and 6 mo; no change at 12 mo	Increase at 6 and 12 wk; no change at 6 or 12 mo	Increase at 6 and 12 wk; no change at 6 mo or 12 mo	No change at 6 wk, 12 wk, 6 mo, or 12 mo
Dacre et al,^27^ 1989	No change at 6 wk or 6 mo	NA	No change at 6 wk or 6 mo	No change at 6 wk or 6 mo	NA	NA
Maryam et al,^38^ 2012	Decrease at 6 wk; no change at 6 mo	Increase at 6 wk; no change at 6 mo	No change at 6 wk or 6 mo	No change at 6 wk or 6 mo	No change at 6 wk or 6 mo	NA
Ryans et al,^47^ 2005	No change at 6 wk or 4 mo	Increase at 6 wk; no change at 4 mo	No change at 6 wk or 4 mo	NA	NA	NA
Lee et al,^33^ 1974	NA	NA	No change at 1, 3, 4, 5, or 6 wk; increase at 2 wk	No change at 1, 3, 4, 5, or 6 wk; increase at 2 wk	NA	NA
Quality of evidence	No change at early short-term (moderate)^a^; No change at mid-term (moderate)^a^	Increase at early short-term (low)^a^^,^^b^; no change at mid-term (low)^a^	No change at early short-term (moderate)^a^; no change at mid-term (high)^a^	No change at early short-term; no change at mid-term	No change at mid-term	NA
**IA corticosteroid vs SA corticosteroid**						
Sun et al,^50^ 2018	Decrease at 4, 8, and 12 wk	Increase at 4, 8, and 12 wk	Increase at 4, 8, and 12 wk	Increase at 4, 8, and 12 wk	Increase at 4, 8, and 12 wk	NA
Khallaf et al,^30^ 2018	No change at 12 wk	No change at 12 wk	No change at 12 wk	No change at 12 wk	No change at 12 wk	NA
Yoon et al,^54^ 2016	No change at 4 wk, 12 wk, or 6 mo	No change at 4 wk, 12 wk, or 6 mo	No change at 4 wk, 12 wk, or 6 mo	NA	No change at 4 wk, 12 wk, or 6 mo	NA
Oh et al,^39^ 2011	Decrease at 3 wk; no change at 6 wk or 12 wk	No change at 3 wk, 6 wk, or 12 wk	No change at 3 wk, 6 wk, or 12 wk	No change at 3 wk, 6 wk, or 12 wk	NA	NA
Shin and Lee,^49^ 2013	No change at 2 wk, 4 wk, 8 wk, 4 mo, or 6 mo	No change at 2 wk, 4 wk, 8 wk, 4 mo, or 6 mo	No change at 2 wk, 4 wk, 8 wk, 4 mo, or 6 mo	NA	No change at 2 wk, 4 wk, 8 wk, 4 mo, or 6 mo	No change at 2 wk, 4 wk, 8 wk, 4 mo, or 6 mo
Cho et al,^26^ 2016	Decrease at 12 wk	Increase at 12 wk	No change at 12 wk	No change at 12 wk	No change at 12 wk	NA
Rizk et al,^45^ 1991	No change at 1-11 wk, 4 mo, or 6 mo	NA	No change at 11 wk or 6 mo	No change at 11 wk or 6 mo	No change at 11 wk or 6 mo	NA
Quality of evidence	Decrease at early short-term (moderate)^a^; no change at late short-term (moderate)^a^; no change at mid-term (moderate)^a^	No change at early short-term (high)^a^; increase at late short-term (high)^a^	No change at early short-term (high)^a^; no change at late short-term (high)^a^; no change at mid-term (high)^a^	Inconclusive at late short-term; no change at late short-term	No change at early short-term; no change at late short-term; No change at mid-term	NA
**Acupuncture with physiotherapy vs physiotherapy only, with or without placebo acupuncture**						
Lo et al,^36^ 2020	No change at 4 wk, 12 wk, or 6 mo	No change at 4 wk, 12 wk, or 6 mo	No change at 4 wk, 12 wk, or 6 mo	No change at 4 wk, 12 wk, or 6 mo	No change at 4 wk, 12 wk, or 6 mo	NA
Koh et al,^31^ 2013	No change at 2, 4, or 12 wk; increase at8 wk,	No change at 2 or 4 wk; increase at 8 and 12 wk	No change at 2, 4, 8, or 12 wk	No change at 2, 4, 8, or 12 wk	No change at 2, 4, 8, or 12 wk	NA
Ma et al,^37^ 2006	Decrease at 4 wk	NA	No change at 4 wk	No change at 4 wk	No change at 4 wk	NA
Quality of evidence	No change at early short-term (low)^a^	NA	No change at early short-term (high)^a^	No change at early short-term; no change at late short-term	No change at early short-term; no change at late short-term	NA
**ESWT with physiotherapy vs physiotherapy only with or without sham ESWT**						
Vahdatpour et al,^52^ 2014	Decrease at 4 wk, 12 wk, and 6 mo	Increase at 4 wk, 12 wk, and 6 mo	Increase at 4 wk, 12 wk, and 6 mo	Increase at 4 wk, 12 wk, and 6 mo	Increase at 4 wk, 12 wk, and 6 mo	NA
Lee et al,^35^ 2017	Decrease at 4 wk	NA	Increase at 4 wk	NA	Increase at 4 wk	NA
Park et al,^77^ 2015	Decrease at 4 wk	Increase at 4 wk	NA	NA	NA	NA
Quality of evidence	Decrease at early short-term (very low)^c^	NA	NA	NA	Increase at early short-term	NA

Abbreviations: ABD, abduction; ER, external rotation; ESWT, extracorporeal shock wave therapy; FL, flexion; IA, intra-articular; NA, not applicable; QoL, quality of life; ROM, range of movement; SA, subacromial.

^a^ Meta-analysis undertaken.

^b^ Results of meta-analysis clinically and statistically significant.

^c^ Meta-analysis abandoned because of very high statistical inconsistency (*I*^2^ > 75%).

### Pairwise Meta-analysis

We conducted pairwise meta-analysis comparing the effectiveness of each intervention with other interventions (or placebo/no treatment) in the short-term (early, 2-6 weeks; late, 8-12 weeks) and mid-term (4-6 months). Data for long-term follow-up (>12 months) were inadequate for analyses. Numerical data are only presented for the statistically significant comparisons; those for nonsignificant comparisons appear in the forest plots (eFigure 1, eFigure 2, and eFigure 3 in the Supplement).

#### IA Corticosteroid vs No Treatment or Placebo

##### Short-term

IA corticosteroid appeared to be associated with superior outcomes compared with control for early short-term pain (moderate certainty; MD, −1.4 visual analog scale [VAS] points; 95% CI, −1.8 to −0.9 VAS points; *P* < .001), ER ROM (high certainty; MD, 4.7°; 95% CI, 2.7° to 6.6°; *P* < .001), and function (high certainty; SMD, 0.6; 95% CI, 0.3 to 0.9; *P* < .001) and late short-term pain (moderate certainty; MD, −1.0 VAS points; −1.5 to −0.5 VAS points; *P* < .001), ER ROM (high certainty; MD, 6.8°; 95% CI, 3.4° to 10.2°; *P* < .001), and function (moderate certainty; SMD, 0.6; 95% CI, 0.3 to 0.8; *P* < .001).

##### Mid-term

IA corticosteroid was associated with better outcomes than control only for function (moderate certainty; SMD, 0.3; 95% CI, 0.1 to 0.5; *P* = .01). However, effects for pain and ER ROM were similar (moderate certainty for both).

#### Physiotherapy vs No Treatment or Placebo

Physiotherapy was found to be associated with improved outcomes compared with control in the early short-term for ER ROM (moderate certainty; MD, 11.3°; 95% CI, 8.6°-14.0°; *P* < .001). Data for other follow-up time periods were insufficient for quantitative analysis.

#### IA Corticosteroid Plus Physiotherapy vs No Treatment or Placebo

Combined treatment with IA corticosteroid plus physiotherapy was associated with superior outcomes vs control for early short-term ER ROM (high certainty; MD, 17.9°; 95% CI, 12.1°-23.7°; *P* < .001). Data for other follow-up periods were insufficient for quantitative analysis.

#### IA Corticosteroid vs Physiotherapy

##### Short-term

IA corticosteroid was associated with significant benefits compared with physiotherapy for early short-term function (moderate certainty; MD, 0.5; 95% CI, 0.2 to 0.7; *P* < .001) and late short-term pain (high certainty; MD, −1.1 VAS points; 95% CI, −1.7 to −0.5 VAS points; *P* < .001) only. Differences for early short-term pain (moderate certainty), late short-term function (moderate certainty), and early and late short-term ER ROM (moderate and high certainty, respectively) were insignificant.

##### Mid-term

IA corticosteroid was associated with better outcomes than physiotherapy for ER ROM (moderate certainty; MD, 4.6°; 95% CI, 0.7°-8.6°; *P* = .02). However, no significant differences in pain (low certainty) or function (moderate certainty) were observed.

#### IA Corticosteroid Plus Physiotherapy vs IA Corticosteroid Only

##### Short-term

Compared with IA corticosteroid alone, combined treatment with IA corticosteroid plus physiotherapy was only associated with superior outcomes for early short-term ER ROM (moderate certainty; MD, 11.6°; 95% CI, 3.7°-19.4°; *P* = .004). Pain and function in the early short-term (moderate and low certainty, respectively) and late short-term function (high certainty) were similar between groups.

##### Mid-term

No significant differences were found between the groups in pain, function, or ER ROM. These results had high, moderate, and high certainty, respectively.

#### IA Corticosteroid Plus Physiotherapy vs Physiotherapy Only

##### Short-term

Combined therapy with IA corticosteroid plus physiotherapy was associated with significant benefits compared with physiotherapy alone only for early short-term function (low certainty; SMD, 0.7; 95% CI, 0.3-1.0; *P* < .001). Differences for early short-term pain and ER ROM and late short-term function were not significant (moderate certainty for all).

##### Mid-term

No significant differences were found between the groups for pain, function, or ER ROM. These comparisons had moderate, low, and high certainty, respectively.

#### IA Corticosteroid vs Subacromial Corticosteroid

##### Short-term

Compared with subacromial administration, administering corticosteroid intra-articularly was only associated with superior outcomes for early short-term pain (moderate certainty; MD, −0.6 VAS points; 95% CI, −1.1 to −0.1 VAS points; *P* = .02) and late short-term function (moderate certainty; SMD, 0.3; 95% CI, 0 to 0.6; *P* = .03). Improvements in late short-term pain (moderate certainty) and ER ROM (high certainty) and early short-term function (high certainty) were similar with the 2 interventions.

##### Mid-term

No significant differences were found between the groups for pain or ER ROM. These comparisons had moderate and high certainty, respectively.

#### Arthrographic Distension Plus IA Corticosteroid vs IA Corticosteroid Only

Adding arthrographic distension to IA corticosteroid appeared to be associated with greater improvements in early and late short-term pain (early: high certainty; MD, −0.9 VAS points; −1.3 to −0.4 VAS points; *P* < .001; late: high certainty; MD, −0.8 VAS points; 95% CI, −1.1 to −0.5 VAS points; *P* < .001). Early and late short-term function (moderate and high certainty, respectively) and early and late short-term ER ROM (high certainty for both) were similar with or without distension.

#### Acupuncture Plus Physiotherapy vs Physiotherapy Only

No differences were found with the addition of acupuncture to physiotherapy for early short-term pain and ER ROM. These comparisons had low and high certainty, respectively.

### Clinically Significant Results and Trial Sequential Analysis

Despite several statistically significant differences in pairwise comparisons, most did not reach the threshold for MCRD. Only IA corticosteroid vs no treatment or placebo for early and late short-term pain and function, physiotherapy with and without IA corticosteroid vs no treatment or placebo for early short-term ER ROM, IA corticosteroid vs physiotherapy for early short-term function and late short-term pain, and combination therapy with IA corticosteroid plus physiotherapy compared with IA corticosteroid for early short-term ER ROM and with physiotherapy for early short-term function reached MCRD.

For the primary outcome measure, the clinically and statistically significant results underwent TSA, which confirmed the results ruling out a type I error in 2 comparisons (IA corticosteroid vs no treatment or placebo for early and late short-term pain) but not in the comparison of IA corticosteroid vs physiotherapy for late short-term pain. This suggests that more studies may be needed to confirm the benefit of IA corticosteroid compared with physiotherapy with more confidence.

eFigures 1 to 3 in the Supplement illustrate the results of the pairwise meta-analyses and associated forest plots for early short-term, late short-term, and mid-term follow up for pain and ER ROM. eFigure 4 in the Supplement illustrates the forest plots for function, and eFigure 5 and eFigure 6 in the Supplement illustrate the TSA graphs.

### Network Meta-analysis

**Figure 2.**
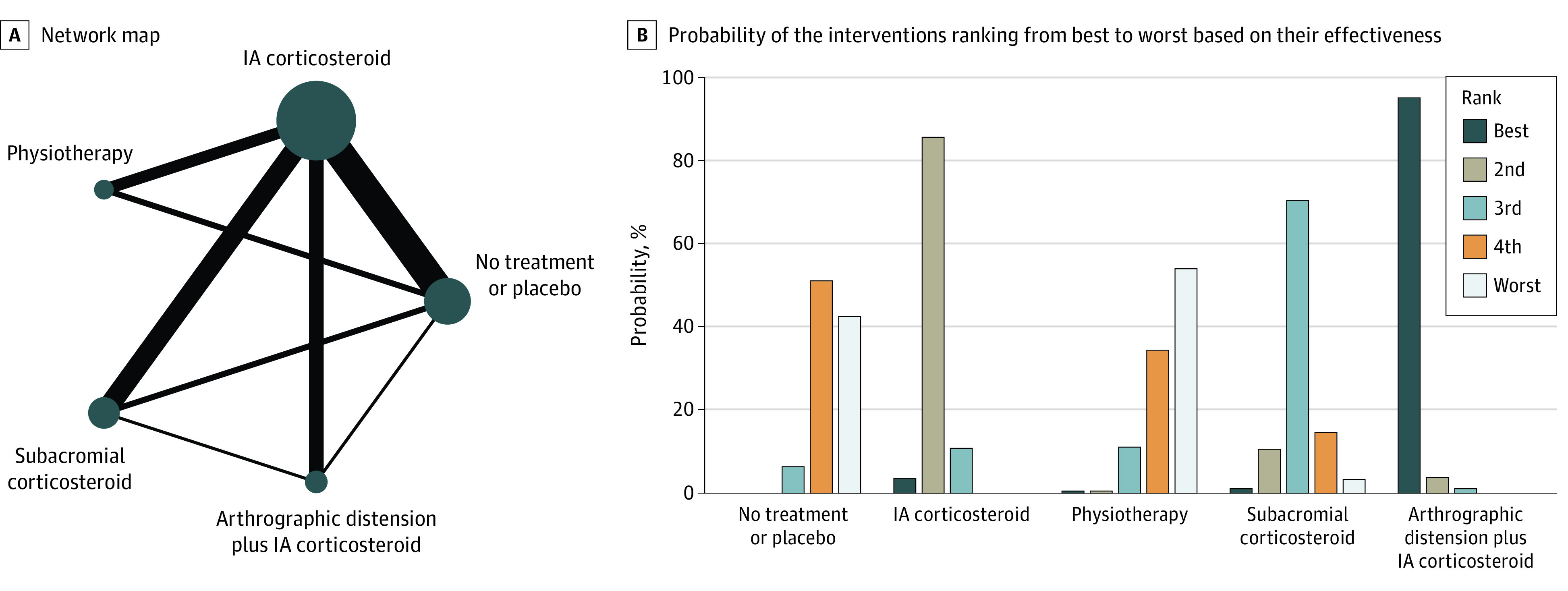
Results of Network Analysis for Pain at Late Short-term (8-12 weeks) Follow-up A, The size of the circles denotes the contribution of participants in each intervention and the thickness of the lines between circles represents the contribution of studies comparing the two interventions. B, The bar graph shows the probability of the 6 interventions ranking from best to worst based on their effectiveness. IA indicates intra-articular.

**Figure 3.**
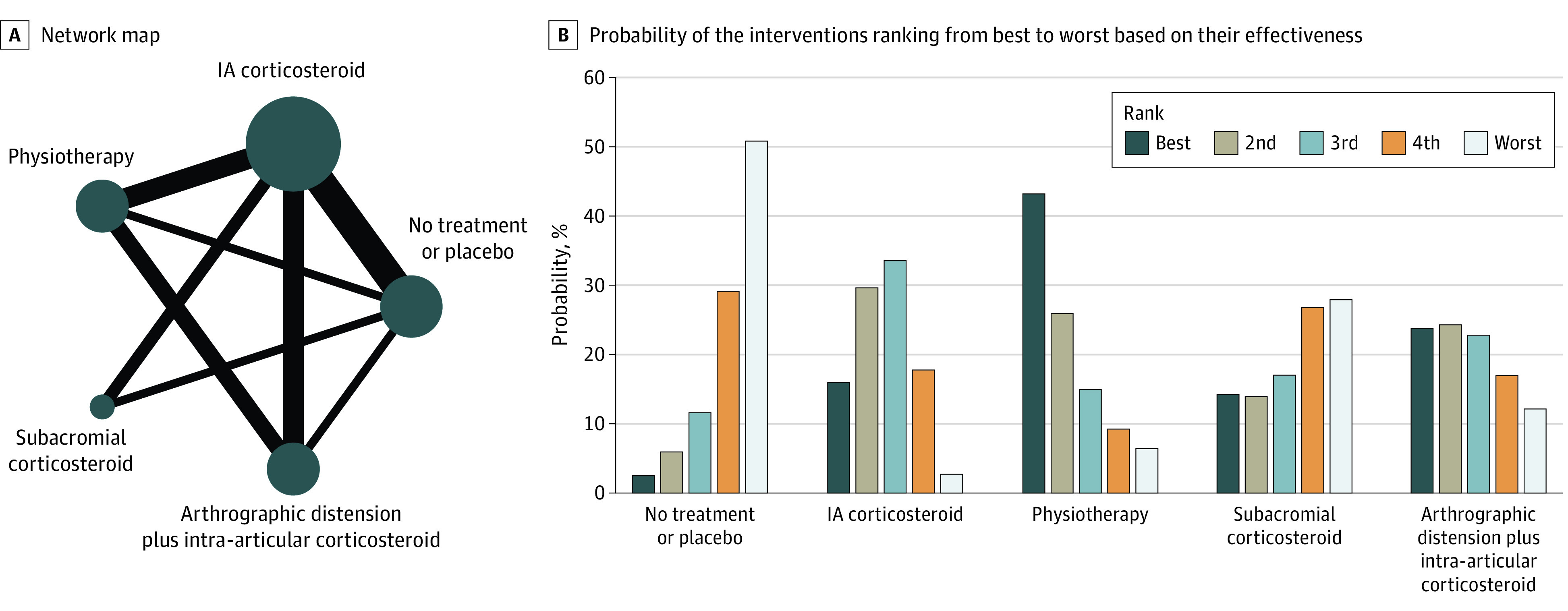
Results of Network Analysis for Pain at Mid-term (4-6 months) Follow-up A, The size of the circles denotes the contribution of participants in each intervention and the thickness of the lines between circles represents the contribution of studies comparing the two interventions. B, The bar graph shows the probability of the 6 interventions ranking from best to worst based on their effectiveness. IA indicates intra-articular.

Figure 2 and Figure 3 show the network maps and treatment rank probabilities for the primary outcome measure (pain) for late short-term (8-12 weeks) and mid-term (4-6 months) follow-up, respectively. eFigure 7 and eFigure 8 in the Supplement illustrates the network forests with their consistency tests.

In the late short-term, arthrographic distension plus IA corticosteroid had the highest probability (96%) of being the most effective treatment. IA corticosteroid had the highest probability (85%) of being the second most effective. Physiotherapy was the least effective treatment, followed by no treatment or placebo. No data existed in the late short-term for combined treatment with IA corticosteroid plus physiotherapy (Figure 2B).

In the mid-term, combined treatment with IA corticosteroid plus physiotherapy had the highest probability (43%) of being the best treatment with physiotherapy. IA corticosteroid had the highest probability (34%) of being the second best treatment. No treatment or placebo followed by subacromial corticosteroid had the highest probability of being the worst interventions (Figure 3B).

### Subgroup Analysis

The potential benefit of home exercise was assessed by comparing the mean improvement in pain in patients who received (1) IA corticosteroid plus a home exercise program vs IA corticosteroid without home exercise, and (2) no treatment or placebo plus home exercise vs no treatment/placebo without home exercise. For the first comparison, a statistically significant (but clinically small) mean benefit of home exercise on pain improvement was identified at 8 to 12 weeks (MD, −0.5 VAS points; 95% CI, −0.9 to −0.1 VAS points; *P* = .01). The benefit of home exercise was much more substantial (clinically and statistically) in those receiving no treatment or placebo (MD, −1.4 VAS points; 95% CI, −1.8 to −1.1 VAS points; *P* < .001). Both results are based on 10 studies^22,24,25,28,42,43,45,46,48,49^ with low overall risk of bias.

Similarly, we assessed for an effect of IA placebo by comparing samples who received IA placebo and no treatment from the IA corticosteroid vs no treatment or placebo comparison. Both subgroups received a home exercise program. Based on 9 studies^22,24,25,28,42,43,45,46,49^ with high overall risk of bias, IA placebo was associated with statistically and clinically significant effects on pain compared with no treatment (MD, −1.6 VAS points; 95% CI, −2.1 to −1.1 VAS points; *P* < .001).

There was insufficient data for a similar subgroup analysis at mid-term follow-up. Subgroup analyses for the effect of chronicity on the effectiveness of treatment modalities could not be evaluated because studies including patients with mixed stages and chronicity of frozen shoulder did not include subgroup data. Finally, subgroup analyses according to physiotherapeutic interventions were not possible because of high clinical heterogeneity (various combinations of modalities and treatment durations used). Most studies used electrotherapy modalities (transcutaneous electrical nerve stimulation, therapeutic ultrasound, diathermy) combined with mobilizations, stretching, or exercises with or without heat and ice packs.

## Discussion

To our knowledge, this is the first systematic review and network meta-analysis to comprehensively analyze all nonsurgical randomized clinical trials pertaining to the treatment of frozen shoulder as well as the largest systematic review ever published in the field. Based on the available evidence, it appears that the use of an IA corticosteroid for patients with frozen shoulder of duration less than 1 year is associated with greater benefits compared with all other interventions, and its benefits may last as long as 6 months. This has important treatment ramifications for the general and specialist musculoskeletal practitioner, providing them with an accessible, cost-effective,^88^ and evidence-based treatment to supplement exercise regimes, which we anticipate will inform national guidelines on frozen shoulder treatments moving forward.

In the short-term, IA corticosteroid appeared to be associated with better outcomes compared with no treatment in all outcome measures. Adding arthrographic distension to IA corticosteroid may be associated with positive effects that last at least as long as 12 weeks compared with IA corticosteroid alone; however, these benefits are probably not clinically significant. Compared with physiotherapy, IA corticosteroid seemed to be associated with better outcomes, with clinically significant differences. Combination therapy with IA corticosteroid plus physiotherapy may be associated with significant benefits compared with IA corticosteroid alone or physiotherapy alone for ER ROM and function, respectively, at 6 weeks. Compared with control, combined IA corticosteroid plus physiotherapy appeared to be associated with an early benefit in ER ROM (as long as 6 weeks), with clinical significance. Subacromial administration of corticosteroid appeared to be as efficacious as IA administration. The addition of acupuncture to physiotherapy did not seem to be associated with any added benefits. Based on the network meta-analysis, arthrographic distension with IA corticosteroid was probably the most effective intervention for pain at 12 weeks follow-up. IA corticosteroid alone ranked second, and as demonstrated by the pairwise meta-analysis, the benefit of adding distension appeared clinically nonsignificant.

Most compared interventions appeared to be associated with similar outcomes at 6-month follow up, without significant differences. The only intervention that was associated with mid-term statistically significant benefits compared with control and physiotherapy (without reaching clinical significance) was IA corticosteroid for function and ER ROM. No mid-term data exist assessing the effectiveness of adding arthrographic distension to IA corticosteroid and acupuncture to physiotherapy or comparing physiotherapy (with or without IA corticosteroid) with no treatment. Our network meta-analysis found that combined therapy with IA corticosteroid and physiotherapy, physiotherapy alone, and IA corticosteroid alone were the most effective interventions for pain at 6 months follow-up. However, according to our pairwise meta-analyses, their clinical benefit compared with other treatments (or even no treatment) appeared very small.

A home exercise program with simple ROM exercises and stretches administered with or without IA corticosteroid appeared to be associated with short-term pain benefits. This was statistically significant but clinically nonsignificant compared with no treatment when accompanied by IA corticosteroid. It was both clinically and statistically significant on its own.

Several systematic reviews have been published assessing the effectiveness of therapeutic interventions for frozen shoulder. Sun et al^89^ looked at the effectiveness if IA corticosteroid by comparing it with no treatment, and their findings were similar to ours, reporting that IA corticosteroid may be associated with benefits on pain, function, and ROM that are most pronounced in the short-term and can last as long as 6 months. The systematic review of both randomized and observational studies by Song et al^90^ is also in agreement with our results, showing a possible early benefit of IA corticosteroid, which likely diminishes in the mid-term. An earlier systematic review by Maund et al,^88^ which was only based on limited evidence (meta-analyses of 2 and 3 studies), was largely inconclusive, demonstrating possible benefits of IA corticosteroid (with and without physiotherapy) in conjunction with a home exercise program. A Cochrane review on arthrographic distension^91^ was also in agreement with our results, showing that arthrographic distension with IA corticosteroid may be associated with short-term benefits in pain, ROM, and function. Their comparison of combined treatment vs IA corticosteroid alone yielded no significant differences; however, it was only based on 2 studies. A 2018 systematic review by Saltychev et al^92^ also supports our findings, having demonstrated a small but clinically insignificant benefit of the addition of arthrographic distension to IA corticosteroid. In their systematic review, Catapano et al^93^ reported that the addition of arthrographic distension to IA corticosteroid may be associated with a clinically significant benefit at 3 months; however, no quantitative analyses were conducted. Finally, a Cochrane review investigating the effects of manual therapy and exercise^94^ concluded that they are probably associated with worse outcomes compared with IA corticosteroid in the short-term, which is in accordance with the findings of the present review, and another study^95^ investigating the effectiveness of electrotherapy modalities was inconclusive because of lack of sufficient evidence.

In this review we aimed to assess the comparative effectiveness of all interventions for frozen shoulder, both surgical and nonsurgical; however, conclusions on the former could not be reached given that included studies did not assess the same interventions, which precluded pooling their results. The existing literature is conflicting regarding the superiority of arthroscopic capsular release (ACR) over nonoperative modalities; De Carli et al^62^ reported no short-term or long-term benefits of ACR plus MUA compared with IA corticosteroid plus physiotherapy in function or ROM. Conversely, Mukherjee et al^75^ found that ACR was associated with significant improvements in pain, function, and ROM compared with IA corticosteroid in the short-term and mid-term. Gallacher et al^63^ demonstrated mixed results, concluding that compared with IA corticosteroid plus arthrographic distension, combined treatment with ACR and IA corticosteroid may be associated with improved function, external rotation, and flexion ROM but not quality of life and abduction ROM in the short-term and mid-term. The risk of complications, where reported, was not higher in the surgical groups.^63^ The existing evidence on MUA, which is not a surgical procedure per se although it is administered under general anesthesia, is more consistent, suggesting its lack of long-term superiority compared with other commonly used nonsurgical treatments or even no treatment.^65,71,76^

Because of the paucity of robust evidence, no firm recommendations exist for clinical practice. The National Institute of Health and Care Excellence (NICE) guidelines,^96^ influenced in turn by the BESS/BOA recommendations, recommend a stepped approach, starting with physiotherapy and only considering IA corticosteroid if there is no, or slow, progress.^96^ With our review, we provide convincing evidence that IA corticosteroid is associated with better short-term outcomes than other treatments, with possible benefits extending in the mid-term; therefore, we recommend its early use with an accompanying home exercise program. This can be supplemented with physiotherapy to further increase the chances of resolution of symptoms by 6 months.

Most patients in the included studies had duration of symptoms of less than 1 year; therefore, our management recommendations are strongest for this subgroup, which includes patients most commonly encountered in clinical practice. Based on the underlying pathophysiology of the condition, usual practice is to only administer IA corticosteroid in the painful and not freezing phase (also advised by NICE guidance^95^); however, this is not backed up by evidence. In our review, studies that included patients with symptoms for more than 1 year reported equally substantial improvements in outcome measures including ROM and function; therefore, the benefits of corticosteroids may also apply to the freezing phase of frozen shoulder.^48,79^

### Limitations

Despite the comprehensiveness and rigor of our methods, which include thorough risk of bias assessments and grading of evidence, we do recognize its limitations. Frozen shoulder of all chronicity was analyzed together; therefore; conclusions about specific stages and their most effective management could not be drawn. Most studies included a home exercise program, but its frequency, intensity, and duration were not taken into account in comparisons nor were separate analyses made adjusting for it. Finally, physiotherapy interventions, regardless of nature and duration, were grouped and analyzed together to minimize imprecision; in reality, some might be more effective than others. However, we only present findings that derived from thorough quantitative analyses, which were in turn substantially reinforced by the TSA, minimizing the risk for type I errors; most previous similar meta-analyses did not use TSA. Additionally, we present the first network meta-analysis including all conservative treatments for frozen shoulder. Furthermore, we based our recommendations on both statistically and clinically significant results.

## Conclusions

Based on the findings of the present review, we recommend the use of IA corticosteroid for patients with frozen shoulder of duration less than 1 year because it appeared to have earlier benefits than other interventions; these benefits could last as long as 6 months. We also recommend an accompanying home exercise program with simple ROM exercises and stretches. The addition of physiotherapy in the form of an electrotherapy modality and supervised mobilizations should also be considered because it may add mid-term benefits and can be used on its own, especially when IA corticosteroid is contra-indicated. Implicated health care professionals should always emphasize to patients that frozen shoulder is a self-limiting condition that usually lasts for a few months but can sometimes take more than 1 year to resolve and its resolution may be expedited by IA corticosteroid. This should be offered at first contact, and an informed decision should be made by the patient after the risks and alternative therapies are presented to them. In the future, other interventions that have shown promising results and currently have inadequate evidence for definitive conclusions (eg, MUA, ACR, specific types of electrotherapy and mobilizations) should be assessed with large, well-designed randomized studies. Finally, future studies should include subgroup analyses assessing the effectiveness of specific interventions on frozen shoulder of different chronicity and stage.

## Notes

Supplement. **eTable 1.** Explanation of the Components of the GRADE Tool and How They Were Assessed **eTable 2. **Risk of Bias Assessments **eTable 3. **Results of Comparisons of Interventions Assessed by Fewer Than 3 Studies and Were Not Pooled Qualitatively or Quantitatively **eTable 4. **Results of Grading of the Certainty of Evidence According to the GRADE Tool for Each Comparison of Interventions **eTable 5. **Results of Statistical Inconsistency Assessment for Each Pairwise Meta-analysis **eFigure 1. **Results of Pairwise Meta-analyses with Respective Mean Differences for Early Short-term Outcomes **eFigure 2. **Results of Pairwise Meta-analyses With Respective Mean Differences for Late Short-term Outcomes **eFigure 3. **Results of Pairwise Meta-analyses With Respective Mean Differences for Mid-term Outcomes **eFigure 4. **Results of Pairwise Meta-analyses With Respective Mean Differences for Function **eFigure 5. **TSA Results for IA Corticosteroid vs No Treatment or Placebo for Early Short-term Pain **eFigure 6. **TSA Results for IA Corticosteroid vs No Treatment or Placebo for Late Short-term Pain **eFigure 7. **Network Forest Plots With Consistency Test for Late Short-term Pain **eFigure 8. **Network Forest Plots With Consistency Test for Mid-term Pain
