# Supplementary material for: Comparison of Treatments for Frozen Shoulder: A Systematic Review and Meta-analysis
Source: JAMA Netw Open. 2020 Dec 16;3(12):e2029581. doi: 10.1001/jamanetworkopen.2020.29581 (PMC7745103; doi:10.1001/jamanetworkopen.2020.29581)
Supplement: Supplement. — eTable 1. Explanation of the Components of the GRADE Tool and How They Were Assessed eTable 2. Risk of Bias Assessments eTable 3. Results of Comparisons of Interventions Assessed by Fewer Than 3 Studies and Were Not Pooled Qualitatively or Quantitatively eTable 4. Results of Grading of the Certainty of Evidence According to the GRADE Tool for Each Comparison of Interventions eTable 5. Results of Statistical Inconsistency Assessment for Each Pairwise Meta-analysis eFigure 1. Results of Pairwise Meta-analyses with Respective Mean Differences for Early Short-term Outcomes eFigure 2. Results of Pairwise Meta-analyses With Respective Mean Differences for Late Short-term Outcomes eFigure 3. Results of Pairwise Meta-analyses With Respective Mean Differences for Mid-term Outcomes eFigure 4. Results of Pairwise Meta-analyses With Respective Mean Differences for Function eFigure 5. TSA Results for IA Corticosteroid vs No Treatment or Placebo for Early Short-term Pain eFigure 6. TSA Results for IA Corticosteroid vs No Treatment or Placebo for Late Short-term Pain eFigure 7. Network Forest Plots With Consistency Test for Late Short-term Pain eFigure 8. Network Forest Plots With Consistency Test for Mid-term Pain [file jamanetwopen-e2029581-s001.pdf]

Supplemental Online Content

Challoumas D, Biddle M, McLean M, Millar NL. Comparison of treatments for frozen shoulder: a systematic review and meta-analysis. *JAMA Netw Open*. 2020;3(12):e2029581. doi:10.1001/jamanetworkopen.2020.29581

eTable 1. Explanation of the Components of the GRADE Tool and How They Were Assessed

eTable 2. Risk of Bias Assessments

eTable 3. Results of Comparisons of Interventions Assessed by Fewer Than 3 Studies and Were Not Pooled Qualitatively or Quantitatively

eTable 4. Results of Grading of the Certainty of Evidence According to the GRADE Tool for Each Comparison of Interventions

eTable 5. Results of Statistical Inconsistency Assessment for Each Pairwise Meta-analysis

eFigure 1. Results of Pairwise Meta-analyses with Respective Mean Differences for Early Short-term Outcomes

eFigure 2. Results of Pairwise Meta-analyses With Respective Mean Differences for Late Short-term Outcomes

eFigure 3. Results of Pairwise Meta-analyses With Respective Mean Differences for Mid-term Outcomes

eFigure 4. Results of Pairwise Meta-analyses With Respective Mean Differences for Function

eFigure 5. TSA Results for IA Corticosteroid vs No Treatment or Placebo for Early Short-term Pain

eFigure 6. TSA Results for IA Corticosteroid vs No Treatment or Placebo for Late Short-term Pain

eFigure 7. Network Forest Plots With Consistency Test for Late Short-term Pain

eFigure 8. Network Forest Plots With Consistency Test for Mid-term Pain

This supplemental material has been provided by the authors to give readers additional information about their work.

eTable 1. Explanation of the Components of the GRADE Tool and How They Were Assessed.

Grading of the certainty of evidence was performed separately for each outcome measure and for each follow up time period for each comparison of interventions. *ER ROM, external rotation range of movement; VAS, visual analogue scale*

GRADE sub-component	Method of assessment
Overall risk of bias	Certainty of evidence was downgraded if the "high overall risk" studies contributed to more than 50% of the weight in the pairwise meta-analysis.
Imprecision of results	Assessed with the optimal information size. This was tested by performing a conventional sample size calculation; if the total number of patients in the included comparisons was lower than that generated by the sample size calculation, the evidence was downgraded. A minimum of 59, 45 and 81 participants were required in each treatment group to detect a minimal clinically relevant difference (MRCD) of 1 point in VAS pain, 10 points in functional scales and 10 degrees in ER ROM respectively at a confidence of 95% (type I error) and power of 80% (type II error)
Inconsistency of evidence	Inconsistency was assessed with tests for heterogeneity (Tau^2, Chi^2 and I^2 tests). Where the inconsistency index defined the heterogeneity as greater than 50% (substantial), sensitivity analyses were performed to identify and remove the studies that were responsible for the inconsistency where possible and the data were re-analysed. No more than one study from each comparison could be removed. Where not possible or in comparisons with 3 or less studies, the evidence was downgraded by one step. Where the I^2 statistic was greater than 75% the meta-analysis was abandoned.
Indirectness of evidence	Assessed by the compared interventions, included populations and outcome measures. Where those were considered to be non-clinically relevant and where there was thought to be significant diversity in the included populations of the compared groups with regard to a) inclusion of patients with specific conditions (e.g. diabetics), b) duration of symptoms and c) home exercise the evidence was downgraded.
Other	Publication bias assessed by the construction of funnel plots where 10 or more studies were included in the same pairwise meta-analysis.

© 2020 Challoumas D et al. *JAMA Network Open.*

eTable 2a. Risk of Bias Assessment for Patient-Reported Outcomes (pain, function).
?, unclear risk

First Author (year)	Internal Validity (Cochrane's Collaboration Tool for Assessing Risk of Bias)							
	Selection bias		*Performance bias*	*Detection bias*	*Attrition bias*	*Reporting bias*	*Other*	*Overall Risk*
	Random sequence generation	*Allocation concealment*	*Blinding of patients and staff*	*Blinding of outcome measures*	*Completeness of outcome data*	*Selective reporting*		
Arslan et al. (2001)	?	?	High	High	Low	High	High	High
Bal et al. (2008)	Low	?	High	High	High	Low	Low	**High**
Binder et al. (1986)	?	?	High	High	Low	High	Low	**High**
Blockey et al. (1954)	?	?	?	?	High	High	High	**High**
Buchbinder et al. (2004a)	Low	Low	Low	Low	Low	Low	High	**Low**
Buchbinder et al. (2004b)	Low	Low	Low	Low	Low	Low	Low	**Low**
Bulgen at al. (1984)	?	?	High	High	Low	High	Low	**High**
Calis et al. (2006)	?	?	High	High	Low	Low	Low	**Low**
Carette et al. (2003)	Low	Low	Low	Low	Low	Low	Low	**Low**
Cheing et al. (2008)	?	High	High	High	Low	Low	High	**High**
	**Internal Validity**							

© 2020 Challoumas D et al. *JAMA Network Open.*

First Author (year)	(Cochrane's Collaboration Tool for Assessing Risk of Bias)							
	Selection bias		***Performance bias***	***Detection bias***	***Attrition bias***	***Reporting bias***	***Other***	***Overall Risk***
	Random sequence generation	***Allocation concealment***	***Blinding of patients and staff***	***Blinding of outcome measures***	***Completeness of outcome data***	***Selective reporting***		
Chen et al. (2014)	Low	Low	High	High	Low	?	Low	**Low**
Cho et al. (2016)	Low	?	High	High	High	low	Low	**High**
Dacre et al. (1989)	?	?	?	?	High	High	Low	**Unclear**
Dahan et al. (1999)	Low	Low	Low	Low	Low	Low	High	**Low**
De Carli et al. (2012)	?	?	High	High	Low	High	?	**High**
Dehghan et al. (2013)	Low	?	High	High	High	High	Low	**High**
Gallacher et al. (2018)	Low	High	High	High	Low	High	Low	**High**
Gam et al. (1998)	Low	High	High	High	Low	Low	Low	**High**
Hsieh et al. (2012)	Low	?	High	High	Low	Low	High	**High**
Jacobs et al. (1991)	?	?	High	High	High	Low	High	**High**
Jacobs et al. (2009)	?	Low	High	High	Low	High	Low	**High**

© 2020 Challoumas D et al. *JAMA Network Open.*

First Author (year)	**Internal Validity (Cochrane's Collaboration Tool for Assessing Risk of Bias)**							
	Selection bias		***Performance bias***	***Detection bias***	***Attrition bias***	***Reporting bias***	***Other***	***Overall Risk***
	Random sequence generation	***Allocation concealment***	***Blinding of patients and staff***	***Blinding of outcome measures***	***Completeness of outcome data***	***Selective reporting***		
Jones & Chattopadhyay (1999)	?	Low	High	High	Low	High	High	**High**
Khallaf et al. (2018)	?	?	High	High	?	High	High	**High**
Khan et al. (2005)	Low	High	High	High	High	?	High	**High**
Kim et al. (2017)	Low	?	?	?	High	Low	Low	**Unclear**
Kivimäki & Pohjolainen (2001)	?	?	High	?	High	High	High	**High**
Kivimäki et al. (2007)	Low	Low	High	High	High	High	?	**High**
Klc et al. (2015)	Low	Low	High	High	Low	Low	High	**High**
Koh et al. (2013)	Low	?	High	High	Low	?	Low	**Low**
Kraal et al. (2018)	Low	Low	?	?	High	Low	Low	**Low**
Lee et al. (1974)	?	?	High	?	?	High	High	**High**

© 2020 Challoumas D et al. *JAMA Network Open.*

First Author (year)	Internal Validity (Cochrane's Collaboration Tool for Assessing Risk of Bias)							
	Selection bias		***Performance bias***	***Detection bias***	***Attrition bias***	***Reporting bias***	***Other***	***Overall Risk***
	Random sequence generation	***Allocation concealment***	***Blinding of patients and staff***	***Blinding of outcome measures***	***Completeness of outcome data***	***Selective reporting***		
Lee et al. (2017a)	Low	?	?	?	Low	Low	Low	**Unclear**
Lee et al. (2017b)	?	?	High	High	?	High	High	**High**
Lim et al. (2014)	Low	Low	?	?	?	High	Low	**Unclear**
Lo et al. (2020)	?	High	?	High	?	?	High	**High**
Lorbach et al. (2010)	?	?	High	High	Low	Low	Low	**Low**
Ma et al. (2006)	?	?	High	High	Low	?	High	**High**
Maryam et al. (2012)	?	?	High	High	High	High	High	**High**
Mukherjee et al. (2017)	Low	?	High	High	Low	Low	?	**Low**
Mun & Baek (2016)	Low	Low	High	High	Low	Low	Low	**Low**
Oh et al. (2011)	Low	Low	?	?	Low	High	Low	**Low**
Park & Hwnag (2000)	High	High	High	High	Low	Low	Low	**High**

© 2020 Challoumas D et al. *JAMA Network Open.*

First Author (year)	Internal Validity (Cochrane's Collaboration Tool for Assessing Risk of Bias)							
	Selection bias		***Performance bias***	***Detection bias***	***Attrition bias***	***Reporting bias***	***Other***	***Overall Risk***
	Random sequence generation	***Allocation concealment***	***Blinding of patients and staff***	***Blinding of outcome measures***	***Completeness of outcome data***	***Selective reporting***		
Park et al. (2013)	Low	?	Low	Low	Low	Low	Low	**Low**
Park et al. (2014)	?	High	High	High	?	Low	High	**High**
Park et al. (2015)	?	?	High	High	?	High	High	**High**
Prestgaard et al. (2015)	Low	Low	High	High	Low	Low	Low	**Low**
Pushpasekaran et al. (2017)	?	?	High	High	?	High	Low	**High**
Quraishi et al. (2007)	Low	?	High	High	Low	High	High	**High**
Ranalletta et al (2015)	Low	?	High	High	low	low	Low	**Low**
Reza et al (2013)	?	Low	Low	Low	Low	Low	Low	**Low**
Rizk et al (1991)	?	?	High	High	High	High	Low	**High**
Roh et al (2011)	Low	Low	High	High	High	Low	Low	**High**

© 2020 Challoumas D et al. *JAMA Network Open.*

First Author (year)	**Internal Validity (Cochrane's Collaboration Tool for Assessing Risk of Bias)**							
	Selection bias		***Performance bias***	***Detection bias***	***Attrition bias***	***Reporting bias***	***Other***	***Overall Risk***
	Random sequence generation	***Allocation concealment***	***Blinding of patients and staff***	***Blinding of outcome measures***	***Completeness of outcome data***	***Selective reporting***		
Rouhani et al. (2016)	Low	?	Low	Low	Low	Low	Low	**Low**
Ryans et al. (2005)	Low	Low	High	High	Low	High	Low	**High**
Schroder et al. (2017)	Low	Low	Low	Low	Low	Low	Low	**Low**
Schydlowsky et al (2012)	?	Low	High	High	High	High	High	**High**
Sharma et al. (2016)	Low	Low	Low	Low	Low	Low	Low	**Low**
Shin & Lee (2013)	Low	Low	High	High	High	Low	?	**High**
Sun et al. (2001)	?	?	High	High	Low	Low	Low	**Low**
Sun et al. (2018)	?	Low	?	Low	Low	High	Low	**Low**
Tveita et al. (2008)	Low	Low	High	High	Low	Low	?	**Low**
Vahdatpour et al. (2014)	Low	?	Low	?	Low	High	High	**Low**
van der Windt et al. (1997)	Low	?	High	High	Low	High	High	**High**

© 2020 Challoumas D et al. *JAMA Network Open.*

First Author (year)	Internal Validity (Cochrane's Collaboration Tool for Assessing Risk of Bias)							
	Selection bias		***Performance bias***	***Detection bias***	***Attrition bias***	***Reporting bias***	***Other***	***Overall Risk***
	Random sequence generation	***Allocation concealment***	***Blinding of patients and staff***	***Blinding of outcome measures***	***Completeness of outcome data***	***Selective reporting***		
Widiastuti-Samekto & Sianturi (2004)	Low	?	?	?	Low	Low	High	**Unclear**
Yoon et al. (2016)	Low	Low	Low	Low	Low	Low	Low	**Low**

© 2020 Challoumas D et al. *JAMA Network Open.*

eTable 2b. Risk of Bias Assessment for Non–Patient-Reported Outcomes (External Rotation Range of Movement)

First Author (year)	Internal Validity (Cochrane's Collaboration Tool for Assessing Risk of Bias)							
	Selection bias		***Performance bias***	***Detection bias***	***Attrition bias***	***Reporting bias***	***Other***	***Overall Risk***
	Random sequence generation	***Allocation concealment***	***Blinding of patients and staff***	***Blinding of outcome measures***	***Completeness of outcome data***	***Selective reporting***		
Arslan et al. (2001)	?	?	High	?	Low	High	High	**High**
Bal et al. (2008)	Low	?	High	Low	High	Low	Low	**Low**
Binder et al. (1986)	?	?	High	Low	Low	High	Low	**Low**
Blockey et al. (1954)	?	?	?	Low	High	High	High	**High**
Buchbinder et al. (2004a)	Low	Low	Low	Low	Low	Low	High	**Low**
Buchbinder et al. (2004b)	Low	Low	Low	Low	Low	Low	Low	**Low**
Bulgen at al. (1984)	?	?	High	?	Low	High	Low	**Unclear**
Calis et al. (2006)	?	?	High	High	Low	Low	Low	**Low**
Carette et al. (2003)	Low	Low	Low	Low	Low	Low	Low	**Low**
Cheing et al. (2008)	?	High	High	High	Low	Low	High	**High**

© 2020 Challoumas D et al. *JAMA Network Open.*

First Author (year)	Internal Validity (Cochrane's Collaboration Tool for Assessing Risk of Bias)							
	Selection bias		***Performance bias***	***Detection bias***	***Attrition bias***	***Reporting bias***	***Other***	***Overall Risk***
	Random sequence generation	***Allocation concealment***	***Blinding of patients and staff***	***Blinding of outcome measures***	***Completeness of outcome data***	***Selective reporting***		
Chen et al. (2014)	Low	Low	High	Low	Low	?	Low	**Low**
Cho et al. (2016)	Low	?	High	low	High	low	Low	**Low**
Dacre et al. (1989)	?	?	?	Low	High	High	Low	**Unclear**
Dahan et al. (1999)	Low	Low	Low	Low	Low	Low	High	**Low**
De Carli et al. (2012)	?	?	High	?	Low	High	?	**Unclear**
Dehghan et al. (2013)	Low	?	High	?	High	High	Low	**High**
Gallacher et al. (2018)	Low	High	High	High	Low	High	Low	**High**
Gam et al. (1998)	Low	High	High	High	Low	Low	Low	**High**
Hsieh et al. (2012)	Low	?	High	Low	Low	Low	High	**Low**
Jacobs et al. (1991)	?	?	High	Low	High	Low	High	**High**
Jacobs et al. (2009)	?	Low	High	?	Low	High	Low	**Low**

© 2020 Challoumas D et al. *JAMA Network Open.*

First Author (year)	Internal Validity (Cochrane's Collaboration Tool for Assessing Risk of Bias)							
	Selection bias		*Performance bias*	*Detection bias*	*Attrition bias*	*Reporting bias*	*Other*	*Overall Risk*
	Random sequence generation	***Allocation concealment***	***Blinding of patients and staff***	***Blinding of outcome measures***	***Completeness of outcome data***	***Selective reporting***		
Jones & Chattopadhyay (1999)	?	Low	High	High	Low	High	High	**High**
Khallaf et al. (2018)	?	?	High	High	?	High	High	**High**
Khan et al. (2005)	Low	High	High	High	High	?	High	**High**
Kim et al. (2017)	Low	?	?	?	High	Low	Low	**Unclear**
Kivimäki & Pohjolainen (2001)	?	?	High	?	High	High	High	**High**
Kivimäki et al. (2007)	Low	Low	High	low	High	High	?	**High**
Klc et al. (2015)	Low	Low	High	Low	Low	Low	High	**Low**
Koh et al. (2013)	Low	?	High	Low	Low	?	Low	**Low**
Kraal et al. (2018)	Low	Low	?	?	High	Low	Low	**Low**
Lee et al. (1974)	?	?	High	?	?	High	High	**High**

© 2020 Challoumas D et al. *JAMA Network Open.*

First Author (year)	Internal Validity (Cochrane's Collaboration Tool for Assessing Risk of Bias)							
	Selection bias		***Performance bias***	***Detection bias***	***Attrition bias***	***Reporting bias***	***Other***	***Overall Risk***
	Random sequence generation	***Allocation concealment***	***Blinding of patients and staff***	***Blinding of outcome measures***	***Completeness of outcome data***	***Selective reporting***		
Lee et al. (2017a)	Low	?	?	Low	Low	Low	Low	**Low**
Lee et al. (2017b)	?	?	High	?	?	High	High	**High**
Lim et al. (2014)	Low	Low	?	Low	?	High	Low	**Low**
Lo et al. (2020)	?	High	?	High	?	?	High	**High**
Lorbach et al. (2010)	?	?	High	?	Low	Low	Low	**Unclear**
Ma et al. (2006)	?	?	High	?	Low	?	High	**Unclear**
Maryam et al. (2012)	?	?	High	?	High	High	High	**High**
Mukherjee et al. (2017)	Low	?	High	?	Low	Low	?	**Unclear**
Mun & Baek (2016)	Low	Low	High	Low	Low	Low	Low	**Low**
Oh et al. (2011)	Low	Low	?	?	Low	High	Low	**Low**
Park & Hwnag (2000)	High	High	High	High	Low	Low	Low	**High**

© 2020 Challoumas D et al. *JAMA Network Open.*

First Author (year)	Internal Validity (Cochrane's Collaboration Tool for Assessing Risk of Bias)							
	Selection bias		***Performance bias***	***Detection bias***	***Attrition bias***	***Reporting bias***	***Other***	***Overall Risk***
	Random sequence generation	***Allocation concealment***	***Blinding of patients and staff***	***Blinding of outcome measures***	***Completeness of outcome data***	***Selective reporting***		
Park et al. (2013)	Low	?	Low	Low	Low	Low	Low	**Low**
Park et al. (2014)	?	High	High	High	?	Low	High	**High**
Park et al. (2015)	?	?	High	?	?	High	High	**High**
Prestgaard et al. (2015)	Low	Low	High	Low	Low	Low	Low	**Low**
Pushpasekaran et al. (2017)	?	?	High	High	?	High	Low	**High**
Quraishi et al. (2007)	Low	?	High	Low	Low	High	High	**High**
Ranalletta et al (2015)	Low	?	High	low	low	low	Low	**Low**
Reza et al (2013)	?	Low	Low	Low	Low	Low	Low	**Low**
Rizk et al (1991)	?	?	High	Low	High	High	Low	**High**
Roh et al (2011)	Low	Low	High	High	High	Low	Low	**High**

© 2020 Challoumas D et al. *JAMA Network Open.*

First Author (year)	Internal Validity (Cochrane's Collaboration Tool for Assessing Risk of Bias)							
	Selection bias		*Performance bias*	*Detection bias*	*Attrition bias*	*Reporting bias*	*Other*	*Overall Risk*
	Random sequence generation	*Allocation concealment*	*Blinding of patients and staff*	*Blinding of outcome measures*	*Completeness of outcome data*	*Selective reporting*		
Rouhani et al. (2016)	Low	?	Low	Low	Low	Low	Low	**Low**
Ryans et al. (2005)	Low	Low	High	Low	Low	High	Low	**Low**
Schroder et al. (2017)	Low	Low	Low	Low	Low	Low	Low	**Low**
Schydlowsky et al (2012)	?	Low	High	High	High	High	High	**High**
Sharma et al. (2016)	Low	Low	Low	Low	Low	Low	Low	**Low**
Shin & Lee (2013)	Low	Low	High	Low	High	Low	?	**Low**
Sun et al. (2001)	?	?	High	Low	Low	Low	Low	**Low**
Sun et al. (2018)	?	Low	?	Low	Low	High	Low	**Low**
Tveita et al. (2008)	Low	Low	High	High	Low	Low	?	**Low**
Vahdatpour et al. (2014)	Low	?	Low	?	Low	High	High	**Low**
van der Windt et al. (1998)	Low	?	High	Low	Low	High	High	**High**

© 2020 Challoumas D et al. *JAMA Network Open.*

First Author (year)	Internal Validity (Cochrane's Collaboration Tool for Assessing Risk of Bias)							
	Selection bias		***Performance bias***	***Detection bias***	***Attrition bias***	***Reporting bias***	***Other***	***Overall Risk***
	Random sequence generation	***Allocation concealment***	***Blinding of patients and staff***	***Blinding of outcome measures***	***Completeness of outcome data***	***Selective reporting***		
Widiastuti-Samekto & Sianturi (2004)	Low	?	?	Low	Low	Low	High	**Low**
Yoon et al. (2016)	Low	Low	Low	Low	Low	Low	Low	**Low**

© 2020 Challoumas D et al. *JAMA Network Open.*

eTable 3. Results of Comparisons of Interventions Assessed by Fewer Than 3 Studies and Were Not Pooled Qualitatively or Quantitatively

Treatment modes	First author (year)	Pain	Functional Disability (SPADI/DASH)	Function (Constant/HAQ/SST)	ROM ER	ROM ABD	ROM FL	Satisfaction
Arthrographic distension + IA Corticosteroid vs placebo/no treatment	**Buchbinder et al. (2004b)**	↓ 3w<br>↔ 6w, 12w	↓ 3w<br>↔ 6w, 12w	-	↔ 3w, 6w, 12w	↑3w<br>↔ 6w, 12w	↔ 3w, 6w, 12w	-
	**Sharma et al. (2016)**	↓ 4w, 8w	↑ 4w, 8w<br>↔ 12m	-	↑ 4w, 8w	↑ 4w, 8w	-	-
Arthrographic distension + IA Corticosteroid vs Arthrographic distension	**Jacobs et al. (1991)**	-	-	-	↔ 4m	↑4m	↑4m	-
Arthrographic distension vs IA Corticosteroid	**Jacobs et al. (1991)**	-	-	-	↔ 4m	↓ 4m	↓ 4m	-
Arthrographic distension + IA Sodium Hyaluronate vs IA Corticosteroid	**Park et al. (2013)**	↔ 2w, 6w	↔ 2w, 6w	-	↑ 2w, 6w	↔ 2w, 6w	↔ 2w, 6w	-
Arthrographic distension + IA Corticosteroid + Physiotherapy vs Physiotherapy	**Khan et al. (2005)**	↔ 8w	-	-	↑ 8w	↑8w	-	-
	**Park et al. (2014)**	↔ 4w	↔ 4w	↔ 4w	↔ 4w	↔ 4w	↔ 4w	-
Arthrographic distension + IA Corticosteroid vs SA Corticosteroid	**Yoon et al. (2016)**	↑ 4w<br>↔ 12w, 6m	-	↑ 4w, 12w<br>↔ 6m	↑ 4w<br>↔ 12w, 6m	-	↑ 4w<br>↔ 12w, 6m	-
Arthrographic distension + MUA vs IA Corticosteroid	**Mun & Baek (2016)**	↓ 2w, 6w, 12w<br>↔ 6m, 12m	-	↑ 2w, 6w, 12w<br>↔ 6m, 12m	↑ 2w, 6w<br>↔ 12w, 6m, 12m	-	↑ 2w, 6w<br>↔ 12w, 6m, 12m	↑ 2w, 6w, 12w<br>↔ 6m, 12m

© 2020 Challoumas D et al. *JAMA Network Open.*

Arthrographic distension vs MUA + IA Corticosteroid	**Quraishi et al. (2007)**	↓ 8w, 6m	-	↑ 8w, 6m	↔ 8w, 6m	↔ 8w, 6m	↔ 8w, 6m	-
Treatment modes	**First author (year)**	**Pain**	**Functional Disability (SPADI/DASH)**	**Function (Constant/HAQ/SST)**	**ROM ER**	**ROM ABD**	**ROM FL**	**Satisfaction**
Arthrographic distension + IA Corticosteroid vs ACR + IA Corticosteroid	**Gallacher et al. (2018)**	-	-	↔ 6w<br>↓ 12w, 6m	↓ 6w, 12w, 6m	↔ 6m	↓ 6m	↔ 6w, 12w, 6m
Arthrographic distension + IA Corticosteroid + Physiotherapy vs Arthrographic distension + IA Corticosteroid	**Park et al. (2014)**	↓ 4w	↓ 4w	↑ 4w	↑ 4w	↑ 4w	↑ 4w	-
Arthrographic distension + IA Corticosteroid vs Physiotherapy	**Park et al. (2014)**	↑ 4w	↔4w	↓ 4w	↓ 4w	↓ 4w	↓ 4w	-
PO Corticosteroid vs ESWT	**Chen et al. (2014)**	↔ 2w, 4w, 6w, 12w	-	↔ 2w<br>↓ 4w, 6w, 12w	↔ 2w, 4w<br>↓ 6w, 12w	↔ 2w, 4w<br>↓ 6w, 12w	↔2w<br>↓ 4w, 6w, 12w	-
Rotator interval Corticosteroid vs SA Corticosteroid	**Sun et al. (2018)**	↓ 4w, 8w, 12w	↓ 4w, 8w, 12w	↑ 4w, 8w, 12w	↑ 4w, 8w, 12w	↑ 4w, 8w, 12w	↑ 4w, 8w, 12w	-
Rotator interval Corticosteroid vs IA Corticosteroid	**Sun et al. (2018)**	↓ 4w, 8w, 12w	↓ 4w, 8w, 12w	↑ 4w, 8w, 12w	↑ 4w, 8w, 12w	↑ 4w, 8w, 12w	↑ 4w, 8w, 12w	-
IA Corticosteroid + Physiotherapy vs Long Head of Biceps Corticosteroid + Physiotherapy	**Lee et al (1974)**	-	-	-	↔ 1w, 3w, 4w, 5w, 6w<br>↑2w	↔ 1w, 3w, 4w, 5w, 6w<br>↑2w	-	-

© 2020 Challoumas D et al. *JAMA Network Open.*

IA + Rotator Interval Corticosteroid vs IA Corticosteroid	**Prestgaard et al (2015)**	↔ 3w, 6w, 12w , 6m	↔ 3w, 6w, 12w, 6m	-	↔ 3w, 6w, 12w, 6m	↔ 3w, 6w, 12w, 6m	↔ 3w, 6w, 12w, 6m	↔ 3w, 6w, 12w, 6m
Treatment modes	**First author (year)**	**Pain**	**Functional Disability (SPADI/DASH)**	**Function (Constant/HAQ/SST)**	**ROM ER**	**ROM ABD**	**ROM FL**	**Satisfaction**
IA + Rotator Interval Corticosteroid vs no treatment	**Prestgaard et al (2015)**	↓ 6w, 12w<br><br>↔ 3w, 6m	↓ 3w, 6w, 12w<br><br>↔ 6m	-	↔ 3w, 6w, 12w, 6m	↑ 12w<br><br>↔ 3w, 6w, 6m	↑ 3w, 6w<br><br>↔ 12w, 6m	↑ 12w<br><br>↔ 3w, 6w, 6m
Long Head of Biceps Corticosteroid + Physiotherapy vs Analgesia	**Lee et al (1974)**	-	-	-	↑1w, 2w, 3w, 4w, 5w, 6w	↑1w, 2w, 3w, 4w, 5w, 6w	-	-
IA + SA Corticosteroid vs no treatment	**Shin & Lee (2013)**	↓ 2w, 4w, 8w, 4m<br><br>↔ 6m	-	↑ 2w, 4w, 8w, 4m	↑ 2w, 4w, 8w, 4m	-	↑ 2w, 4w, 8w, 4m<br><br>↔ 6m	↑ 2w, 4w, 8w, 4m<br><br>↔ 6m
IA Corticosteroid vs IA + SA Corticosteroid	**Shin & Lee (2013)**	↔ 2w, 4w, 8w, 4m, 6m	-	↔ 2w, 4w, 8w, 4m, 6m	↔ 2w, 4w, 8w, 4m, 6m	-	↔ 2w, 4w, 8w, 4m, 6m	↔ 2w, 4w, 8w, 4m, 6m
	**Cho et al (2016)**	↔ 12w	-	↔ 12w	↔12w	↔12w	↔12w	-
SA Corticosteroid vs IA + SA Corticosteroid	**Shin & Lee (2013)**	↔ 2w, 4w, 8w, 4m, 6m	-	↔ 2w, 4w, 8w, 4m, 6m	↔ 2w, 4w, 8w, 4m, 6m	-	↔ 2w, 4w, 8w, 4m, 6m	↔ 2w, 4w, 8w, 4m, 6m
	**Cho et al (2016)**	↓ 12w	-	↑ 12w	↔ 12w	↔ 12w	↔ 12w	-
SA Corticosteroid vs no treatment	**Shin & Lee (2013)**	↓ 2w, 4w, 8w, 4m<br><br>↔ 6m	-	↑ 2w, 4w, 8w, 4m<br><br>↔ 6m	↑ 2w, 4w, 8w, 4m<br><br>↔ 6m	-	↑ 2w, 4w, 8w, 4m<br><br>↔ 6m	↑ 2w, 4w, 8w, 4m<br><br>↔ 6m
	**Rizk et al. (1991)**	↔ 1-11w, 4m, 6m	-	-	-	-	-	-

© 2020 Challoumas D et al. *JAMA Network Open.*

IA Corticosteroid vs three-site Corticosteroid	**Pushpasekaran et al. (2017)**	↑ 3w, 6w<br>↔ 6m	-	↓ 3w, 6w, 6m	-	-	-	-
Treatment modes	**First author (year)**	**Pain**	**Functional Disability (SPADI/DASH)**	**Function (Constant/HAQ/SST)**	**ROM ER**	**ROM ABD**	**ROM FL**	**Satisfaction**
IA Corticosteroid vs PO Corticosteroid	**Lorbach et al (2010)**	↔ 4w, 8w, 12w, 6m, 12m	-	↑ 4w, 8w, 12w, 6m, 12m	↑ 4w, 8w<br>↔ 12w, 6m, 12m	↑ 8w, 6m, 12m<br>↔ 4w, 12w	↑ 4w, 8w, 12w<br>↔ 6m, 12m	-
IA Corticosteroid + Physiotherapy vs PO Corticosteroid + Physiotherapy	**Widiastuti-Samekto & Sianturi (2003)**	↓ 1w<br>↔ 2w, 3w	-	-	-	-	-	-
PO Corticosteroid vs Placebo/no treatment	**Blockey et al (1954)**	↔ 4m	-	-	-	NS	-	-
	**Buchbinder et al (2004a)**	↓ 3w<br>↔ 6w<br>↑ 12w	↓ 3w<br>↔ 6w<br>↑ 12w	↑ 3w<br>↔ 6w<br>↓ 12w	↔ 3w, 6w, 12w	↑ 3w<br>↔ 6w<br>↓ 12w	↑ 3w<br>↔ 6w<br>↓ 12w	↔ 3w, 6w, 12w
	**Binder et al. (1986)**	↔ 2w, 4w, 6w, 12w, 5m, 6m, 7m, 8m	-	-	↔ 2w, 4w, 6w, 12w, 5m, 6m, 7m, 8m	↔ 2w, 4w, 6w, 12w, 5m, 6m, 7m, 8m	↔ 2w, 4w, 6w, 12w, 5m, 6m, 7m, 8m	-
IA Sodium hyaluronate vs Physiotherapy	**Calis et al (2006)**	↔ 2w, 12w	-	↓ 2w<br>↔12w	↓ 2w, 12w	↓ 2w<br>↔12w	-	-
IA Sodium hyaluronate vs IA Corticosteroid	**Calis et al (2006)**	↔ 2w, 12w	-	↔ 2w, 12w	↔ 2w, 12w	↔ 2w, 12w	-	-
IA Sodium hyaluronate vs no treatment	**Calis et al (2006)**	↔ 2w, 12w	-	↔ 2w<br>↑ 12w	↔ 2w, 12w	↔ 2w, 12w	-	-

© 2020 Challoumas D et al. *JAMA Network Open.*

IA Sodium hyaluronate + IA Tramadol vs IA Sodium Hyaluronate	**Kim et al. (2017)**	↓ 1w, 2w<br>↔ 3w, 4w, 6w	↔ 1w, 2w, 3w, 4w, 6w	↔ 1w, 2w, 3w, 4w, 6w	↔ 1w, 2w, 3w, 4w, 6w	↔ 1w, 2w, 3w, 4w, 6w	-	-
Treatment modes	**First author (year)**	**Pain**	**Functional Disability (SPADI/DASH)**	**Function (Constant/HAQ/SST)**	**ROM ER**	**ROM ABD**	**ROM FL**	**Satisfaction**
IA Sodium Hyaluronate + Physiotherapy vs Physiotherapy	**Hsieh et al. (2012)**	↔ 6w, 12w	↔ 6w, 12w	-	↔ 6w, 12w	↔ 6w, 12w	↔ 6w, 12w	↔ 6w, 12w
IA Corticosteroid vs IA Sodium Hyaluronate	**Lim et al (2014)**	↔ 2w, 12w	-	↔ 2w, 12w	↔ 12w	-	↔ 12w	-
IA Corticosteroid vs Adilubimab	**Schydlowsky et al (2012)**	-	↔ 2w, 4w, 8w, 12w, 6m	↔ 2w, 4w, 8w, 12w, 6m	↔ 2w, 4w, 8w, 12w, 6m	↔ 2w, 4w, 8w, 12w, 6m	↔ 2w, 4w, 8w, 12w, 6m	-
Suprascapular nerve block vs Placebo	**Dahan et al. (1999)**	↔ 4w	↔ 4w		↔ 4w	↔ 4w	↔ 4w	-
Suprascapular nerve block vs IA Corticosteroid	**Jones & Chattopadhyay (1999)**	↓ 12w	-	-	↑ 12w	↑ 12w	-	-
Suprascapular nerve block + Physiotherapy vs Physiotherapy (+/- placebo)	**Dahan et al. (2000)**	↓ 4w	-	↔ 4w	↔ 4w	↔ 4w	↔ 4w	-
	**Klc et al. (2015)**	↓ 3w, 7w	-	↑ 3w<br>↔ 7w	-	-	-	-
Intranasal calcitonin + Physiotherapy vs intransal Placebo + Physiotherapy	**Rouhani et al. (2016)**	↓ 6w	↑ 6w		↑ 6w	↑ 6w	↑ 6w	↑ 6w
MUA + ACR vs IA Corticosteroid + Physiotherapy	**De Carli et al. (2012)**	-	-	↔ 6w, 12m	↔ 6w, 12m	↔ 6w, 12m	↔ 6w, 12m	-

© 2020 Challoumas D et al. *JAMA Network Open.*

MUA vs IA Corticosteroid + Arthrographic distension	**Jacobs et al. (2009)**	↔ 2w, 6w, 12w, 4m	-	↔ 2w, 6w, 12w, 4m	-	-	-	↔ 2w, 6w, 12w, 4m
Treatment modes	**First author (year)**	**Pain**	**Functional Disability (SPADI/DASH)**	**Function (Constant/HAQ/SST)**	**ROM ER**	**ROM ABD**	**ROM FL**	**Satisfaction**
MUA + IA Corticosteroid vs MUA	**Kivimäki & Pohjolainen (2001)**	-	-	-	↔ 4m	↔ 4m	↔ 4m	-
MUA vs no treatment	**Kivimäki et al. (2007)**	↔ 6w, 12w, 6m, 12m	↔ 6w, 12w, 6m, 12m	↔ 6w, 12w, 6m, 12m	↔ 6w, 12w, 6m, 12m	↔ 6w, 12w, 6m, 12m	↔ 6w 6m, 12m<br>↑ 12w	-
ACR vs IA Corticosteroid	**Mukherjee et al. (2017)**	↔ 4w<br>↓ 8w, 12w, 4m, 5m	-	↑ 4w, 8w, 12w, 4m, 5m	↑ 4w, 8w, 12w, 4m, 5m	↑ 4w, 8w, 12w, 4m, 5m	↑ 4w, 8w, 12w, 4m, 5m	-
Acupuncture + Physiotherapy vs Acupuncture	**Ma et al. (2006)**	↓ 4w	-	-	↔ 4w	↔ 4w	↔ 4w	-
Acupuncture vs physiotherapy	**Cheing et al. (2008)**	↔ 4w, 12w, 6m	-	↔ 4w, 12w, 6m	-	-	-	-
	**Ma et al. (2006)**	↓ 4w	-	-	↔ 4w	↔ 4w	↔ 4w	-
Acupuncture vs sham acupuncture/no treatment	**Cheing et al. (2008)**	↓ 4w	-	↑ 4w	-	-	-	-
	**Schroder et al. (2017)**	↓(post session)	-	↔ (post session)	-	-	-	-
	**Sun et al. (2001)**	-	-	↑ 6w, 5m	-	-	-	-

ACR, arthroscopic capsular release; ESWT, extracorporeal shock wave therapy; IA, intra-articular; m, months; MUA, manipulation under anaesthesia; PO, per oral; SA, subacromial; w; weeks

© 2020 Challoumas D et al. *JAMA Network Open.*

eTable 4. Results of Grading of the Certainty of Evidence According to the GRADE Tool for Each Comparison of Interventions

Comparison	Outcome measure	Number of studies	Overall risk of bias	Inconsistency	Indirectness	Imprecision	Other	Strength of Evidence
**Arthrographic distension + IA Corticosteroid vs IA Corticosteroid only**	**Pain**	4 EST<br>5 LST	Low	Low	Low	Low	Low	**High EST**<br>**High LST**
	**Functional Disability**	3 EST<br>4 LST	Low	High EST<br>Low LST	Low	Low	Low	**Mod EST**<br>**High LST**
	**ROM ER**	3 EST<br>5 LST	Low	Low	Low	Low	Low	**High EST**<br>**High LST**
**Physiotherapy vs no treatment/placebo**	**ROM ER**	4 EST	Low	Low	Low	High	Low	**Mod EST**
**IA Corticosteroid vs IA Placebo/No treatment**	**Pain**	11 EST<br>10 LST<br>7 MT	Low EST<br>Low LST<br>High MT	Low EST<br>High LST<br>Low MT	Low	Low	Low (Funnel plots for EST and LST)	**High EST**<br>**Mod LST**<br>**Mod MT**
	**Functional Disability**	9 EST<br>8 LST<br>5 MT	Low EST<br>Low LST<br>High MT	High EST<br>High LST<br>Low MT	Low	Low		**Mod EST**<br>**Mod LST**<br>**Mod MT**

© 2020 Challoumas D et al. *JAMA Network Open.*

	**ROM ER**	11 EST	Low EST	Low	Low	Low	Low	**High EST**
**Comparison**	**Outcome measure**	**Number of studies**	**Overall risk of bias**	**Inconsistency**	**Indirectness**	**Imprecision**	**Other**	**Strength of Evidence**
		11 LST<br>7 MT	Low LST<br>High MT				(Funnel plots for EST and LST)	**High LST**<br>**Mod MT**
**IA Corticosteroid + Physiotherapy vs IA Placebo/no treatment**	**ER ROM**	3 EST	Low	Low	Low	Low	Low	**High EST**
**IA Corticosteroid vs Physiotherapy**	**Pain**	7 EST<br>4 LST<br>5 MT	High EST<br>Low LST<br>High MT	Low EST<br>Low LST<br>High MT	Low	Low	Low	**MOD EST**<br>**High LST**<br>**LOW MT**
	**Functional Disability**	5 EST<br>3 LST<br>4 MT	High EST<br>Low LST<br>High MT	Low EST<br>High LST<br>Low MT	Low	Low	Low	**MOD EST**<br>**MOD LST**<br>**MOD MT**
	**ROM ER**	6 EST<br>4 LST<br>4 MT	Low EST<br>Low LST<br>High MT	High EST<br>Low LST<br>Low MT	Low	Low	Low	**MOD EST**<br>**HIGH LST**<br>**MOD MT**

© 2020 Challoumas D et al. *JAMA Network Open.*

**Comparison**	**Outcome measure**	**Number of studies**	**Overall risk of bias**	**Inconsistency**	**Indirectness**	**Imprecision**	**Other**	**Strength of Evidence**
**IA Corticosteroid + Physiotherapy vs IA Corticosteroid only**	**Pain**	4 EST<br>5 MT	High	Low	Low	Low	Low	**MOD EST**<br>**MOD MT**
	**Functional Disability**	4 EST<br>4 MT	High EST<br>Low MT	Low	Low	High EST<br>Low MT	Low	**LOW EST**<br>**HIGH MT**
	**ROM ER**	4 EST<br>4 MT	Low	High EST<br>Low MT	Low	Low	Low	**MOD EST**<br>**HIGH MT**
**IA Corticosteroid + Physiotherapy vs Physiotherapy only**	**Pain**	4 EST<br>4 MT	High	Low	Low		Low	**MOD EST**<br>**MOD MT**
	**Functional Disability**	3 EST<br>3 MT	High	Low	Low	High	Low	**LOW EST**<br>**LOW MT**
	**ROM ER**	4 EST<br>3 MT	High EST<br>Low MT	Low	Low	Low	Low	**MOD EST**<br>**HIGH MT**
**IA Corticosteroid vs SA Corticosteroid**	**Pain**	6 EST<br>7 LST	Low EST<br>High LST	High EST<br>Low LST	Low	Low	Low	**MOD EST**<br>**MOD LST**

© 2020 Challoumas D et al. *JAMA Network Open.*

		3 MT	Low MT	High MT				**MOD MT**
**Comparison**	**Outcome measure**	**Number of studies**	**Overall risk of bias**	**Inconsistency**	**Indirectness**	**Imprecision**	**Other**	**Strength of Evidence**
**IA Corticosteroid vs SA Corticosteroid**	**Function**	5 EST<br>6 LST	Low	Low	Low	Low	Low	**HIGH EST**<br>**HIGH LST**
	**ROM ER**	5 EST<br>6 LST<br>3 MT	Low	Low	Low	Low	Low	**HIGH EST**<br>**HIGH LST**<br>**HIGH MT**
**Acupuncture + Physiotherapy vs Physiotherapy only (+/- placebo)**	**Pain**	3 EST	High	Low	Low	High	Low	**LOW EST**
	**ROM ER**	3 EST	Low	Low	Low	Low	Low	**HIGH EST**
**ESWT + Physiotherapy vs Physiotherapy only (+/- sham ESWT)**	**Pain**	3 EST	High	Too high	Low	High	Low	**Meta-analysis abandoned**

Each outcome measure and each follow up time period graded separately

ER ROM, external rotation range of movement; EST, early short-term (2-6 weeks); ESWT, extracorporeal shock wave therapy; IA, intra-articular; LST, late short-term (8-12 weeks); MT, mid-term (4-6 months); SA, subacromial.

© 2020 Challoumas D et al. *JAMA Network Open.*

eTable 5. Results of Statistical Inconsistency Assessment for Each Pairwise Meta-analysis

Comparison	Outcome Measure	Follow up time period	I^2	Study removed after sensitivity analysis	I^2
IA Corticosteroid vs No Treatment/Placebo	Pain	Early short-term	46%	-	-
		Late short-term	80%	Rizk et al. (1991)	72%
		Mid-term	72%	Prestgaard et al. (2015)	48%
	Function	Early short-term	83%	Ranalletta et al (2015)	64%
		Late short-term	81%	Ranalletta et al (2015)	52%
		Mid-term	0%	-	-
	ER ROM	Early short-term	20%	-	-
		Late short-term	53%	Ranalletta et al (2015)	48%
		Mid-term	31%	-	-
Physiotherapy vs No Treatment/Placebo	ER ROM	Early short-term	95%	Carette et al. (2003)	47%
IA Corticosteroid + Physiotherapy vs No Treatment/Placebo	ER ROM	Early short-term	0%	-	-
IA Corticosteroid vs Physiotherapy	Pain	Early short-term	56%	Van der Windt et al. (1998)	23%
		Late short-term	22%	-	-
		Mid-term	66%	-	-

© 2020 Challoumas D et al. *JAMA Network Open.*

	**Function**	**Early short-term**	66%	Calis et al. (2006)	0%
**Comparison**	**Outcome Measure**	**Follow up time period**	**I^2**	**Study removed after sensitivity analysis**	**I^2**
**IA Corticosteroid vs Physiotherapy**	**Function**	**Late short-term**	65%	-	-
		**Mid-term**	17%	-	-
	**ER ROM**	**Early short-term**	73%	-	-
		**Late short-term**	61%	Bulgen et al. (1984)	0%
		**Mid-term**	0%	-	-
**IA Corticosteroid + Physiotherapy vs IA Corticosteroid**	**Pain**	**Early short-term**	77%	Kraal et al. (2018)	0%
		**Mid-term**	1%	-	-
	**Function**	**Early short-term**	77%	Kraal et al. (2018)	0%
		**Mid-term**	0%	-	-
	**ER ROM**	**Early short-term**	87%	Maryam et al. (2012)	52%
		**Mid-term**	45%	-	-
**IA Corticosteroid + Physiotherapy vs Physiotherapy**	**Pain**	**Early short-term**	76%	Carette et al. (2003)	0%
		**Mid-term**	23%	-	-
	**Function**	**Early short-term**	7%	-	-

© 2020 Challoumas D et al. *JAMA Network Open.*

		**Mid-term**	2%	-	-
**Comparison**	**Outcome Measure**	**Follow up time period**	**I²**	**Study removed after sensitivity analysis**	**I²**
**IA Corticosteroid + Physiotherapy vs Physiotherapy**	**ER ROM**	**Early short-term**	53%	Carette et al. (2003)	0%
		**Mid-term**	92%	-	-
**IA Corticosteroid vs SA Corticosteroid**	**Pain**	**Early short-term**	95%	Cho et al. (2016)	60%
		**Late short-term**	52%	Cho et al. (2016)	22%
		**Mid-term**	78%	-	-
	**Function**	**Early short-term**	70%	Cho et al. (2016)	0%
		**Late short-term**	57%	Cho et al. (2016)	43%
	**ER ROM**	**Early short-term**	42%	-	-
		**Late short-term**	67%	Sun et al. (2018)	43%
		**Mid-term**	28%	-	-
**Arthrographic Distension + IA Corticosteroid vs IA Corticosteroid**	**Pain**	**Early short-term**	0%	-	-
		**Late short-term**	51%	Gam et al. (1998)	0%
	**Function**	**Early short-term**	61%	-	-
		**Late short-term**	0%	-	-

© 2020 Challoumas D et al. *JAMA Network Open.*

	**ER ROM**	**Early short-term**	18%	-	-
**Comparison**	**Outcome Measure**	**Follow up time period**	**I^2**	**Study removed after sensitivity analysis**	**I^2**
**Arthrographic Distension + IA Corticosteroid vs IA Corticosteroid**	**ER ROM**	**Late short-term**	85%	Reza et al. (2015)	0%
**Acupuncture + Physiotherapy vs Physiotherapy**	**Pain**	**Early short-term**	0%	-	-
	**ER ROM**	**Early short-term**	0%	-	-
**ESWT + Physiotherapy vs Physiotherapy only (+/- sham ESWT)**	**Pain**	**Early short-term**	93%*	-	-

Where the I^2 statistic was greater than 50% and there were at least four studies in the meta-analysis, sensitivity analyses were conducted to identify and remove a single study which was responsible for the high heterogeneity and the test was re-performed. *ER ROM, external rotation range of movement*

*Sensitivity analysis not performed as only three studies in meta-analysi

© 2020 Challoumas D et al. *JAMA Network Open.*

eFigure 1. Results of Pairwise Meta-analyses with Respective Mean Differences for Early Short-term Outcomes

Early short-term results (2-6 weeks)

a) IA Corticosteroid vs No Treatment/Placebo

Pain

Study or Subgroup	IA Corticosteroid Mean [VAS]	IA Corticosteroid SD [VAS]	IA Corticosteroid Total	No treatment/Placebo Mean [VAS]	No treatment/Placebo SD [VAS]	No treatment/Placebo Total	Weight	Mean Difference IV, Random, 95% CI [VAS]
Bal et al. (2008)	−4.8	3.5	42	−2	3.9	40	6.0%	−2.80 [−4.41, −1.19]
Calis et al. (2006)	−1.2	1.9	25	−0.8	2.2	20	8.9%	−0.40 [−1.62, 0.82]
Carette et al. (2003)	−3.9	2.8	25	−1.7	2.7	23	6.3%	−2.20 [−3.76, −0.64]
Dehghan et al. (2013)	−3.3	2.2	29	−2.5	2.3	28	9.4%	−0.80 [−1.97, 0.37]
Prestgaard et al. (2015)	−2.9	1.8	42	−1.5	1.8	40	14.5%	−1.40 [−2.18, −0.62]
Ranalletta et al. (2015)	−5.3	0.6	37	−3.8	0.9	37	22.2%	−1.50 [−1.85, −1.15]
Rizk et al. (1991)	0	1.9	16	−0.2	2.2	16	7.2%	0.20 [−1.22, 1.62]
Roh et al. (2012)	−1.2	2.7	25	−0.3	2.7	25	6.7%	−0.90 [−2.40, 0.60]
Ryans et al. (2005)	−1	1.9	20	−0.5	2.8	20	6.8%	−0.50 [−1.98, 0.98]
Sharma et al. (2016)	−3.1	1.8	36	−1	2.3	36	11.9%	−2.10 [−3.05, −1.15]
Shin & Lee (2013)	−5.5	1.9	48	−2.3	1.2	49	0.0%	−3.20 [−3.83, −2.57]
Total (95% CI)			297			285	100.0%	−1.28 [−1.74, −0.83]

Heterogeneity: $Tau^2 = 0.21$; $Chi^2 = 16.61$, df = 9 ($P = 0.06$); $I^2 = 46\%$
Test for overall effect: $Z = 5.58$ ($P < 0.00001$)

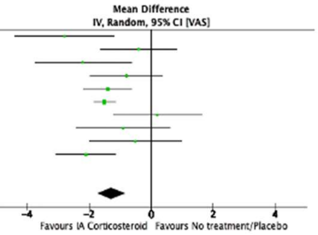

ER ROM

Study or Subgroup	IA Corticosteroid Mean [degrees]	IA Corticosteroid SD [degrees]	IA Corticosteroid Total	No Treatment/Placebo Mean [degrees]	No Treatment/Placebo SD [degrees]	No Treatment/Placebo Total	Weight	Mean Difference IV, Random, 95% CI [degrees]
Bal et al. (2008)	−25	38.1	42	−10	8.1	24	2.7%	−15.00 [−26.97, −3.03]
Calis et al. (2006)	−7.5	11.5	25	−7	9.2	20	9.0%	−0.50 [−6.55, 5.55]
Carette et al. (2003)	−18.3	16.3	25	−7.1	16.3	23	4.3%	−11.20 [−20.43, −1.97]
Dehghan et al. (2013)	−11.4	11.7	29	−9.4	8.9	28	10.8%	−2.00 [−7.39, 3.39]
Prestgaard et al. (2015)	−13.7	11.8	42	−7	11.7	40	11.8%	−6.70 [−11.79, −1.61]
Ranalletta et al. (2015)	−16.8	7.5	36	−13.7	7.5	38	19.9%	−3.10 [−6.52, 0.32]
Rizk et al. (1991)	−0.5	12.6	16	−0.8	12	16	5.0%	0.30 [−8.23, 8.83]
Roh et al. (2012)	−4	9	25	−1	9	25	12.1%	−3.00 [−7.99, 1.99]
Ryans et al. (2005)	−14.3	15.2	20	−6.6	13.2	20	4.7%	−7.70 [−16.52, 1.12]
Sharma et al. (2016)	−10.5	15.5	36	−1.5	14.1	36	7.3%	−9.00 [−15.84, −2.16]
Shin & Lee (2013)	−14	12.6	48	−10	12	49	12.4%	−4.00 [−8.90, 0.90]
Total (95% CI)			344			319	100.0%	−4.42 [−6.44, −2.40]

Heterogeneity: $Tau^2 = 2.28$; $Chi^2 = 12.53$, df = 10 ($P = 0.25$); $I^2 = 20\%$
Test for overall effect: $Z = 4.29$ ($P < 0.0001$)

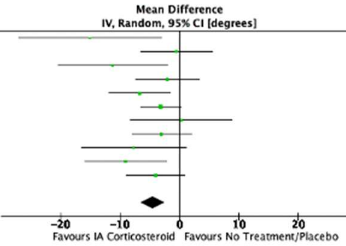

b) Physiotherapy vs No Treatment/Placebo

ER ROM

Study or Subgroup	Physiotherapy Mean [degrees]	Physiotherapy SD [degrees]	Physiotherapy Total	No Treatment/Placebo Mean [degrees]	No Treatment/Placebo SD [degrees]	No Treatment/Placebo Total	Weight	Mean Difference IV, Random, 95% CI [degrees]
Bulgen et al. (1984)	−15	3.2	11	−4	3.4	8	37.9%	−11.00 [−14.02, −7.98]
Calis et al. (2006)	−13.8	11.2	21	−7	9.2	20	14.8%	−6.80 [−13.06, −0.54]
Carette et al. (2003)	−9.6	3.2	27	−7.1	3.4	23	0.0%	−2.50 [−4.34, −0.66]
Lee et al. (1974)	−14	3.2	17	−1	3.4	15	47.4%	−13.00 [−15.30, −10.70]
Total (95% CI)			49			43	100.0%	−11.33 [−14.03, −8.63]

Heterogeneity: $Tau^2 = 2.63$; $Chi^2 = 3.77$, df = 2 ($P = 0.15$); $I^2 = 47\%$
Test for overall effect: $Z = 8.23$ ($P < 0.00001$)

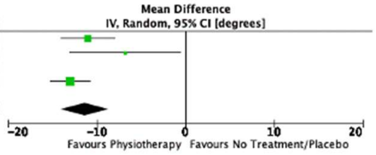

c) IA Corticosteroid + Physiotherapy vs No Treatment/Placebo

ER ROM

Study or Subgroup	IA Corticosteroid + Physiotherapy Mean [degrees]	IA Corticosteroid + Physiotherapy SD [degrees]	IA Corticosteroid + Physiotherapy Total	No treatment/Placebo Mean [degrees]	No treatment/Placebo SD [degrees]	No treatment/Placebo Total	Weight	Mean Difference IV, Random, 95% CI [degrees]
Carette et al. (2003)	−26.5	16.9	22	−7.1	17.7	27	35.3%	−19.40 [−29.12, −9.68]
Lee et al. (1974)	−22	16.7	15	−1	15.5	17	26.5%	−21.00 [−32.21, −9.79]
Ryans et al. (2005)	−21	16.5	20	−6.6	13.2	19	38.1%	−14.40 [−23.76, −5.04]
Total (95% CI)			57			63	100.0%	−17.92 [−23.69, −12.14]

Heterogeneity: $Tau^2 = 0.00$; $Chi^2 = 0.92$, df = 2 ($P = 0.63$); $I^2 = 0\%$
Test for overall effect: $Z = 6.08$ ($P < 0.00001$)

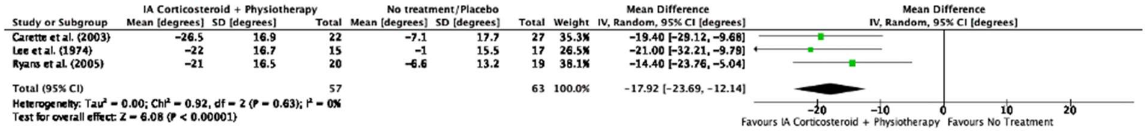

d) IA Corticosteroid vs Physiotherapy

Pain

Study or Subgroup	IA Corticosteroid Mean [VAS]	IA Corticosteroid SD [VAS]	IA Corticosteroid Total	Physiotherapy Mean [VAS]	Physiotherapy SD [VAS]	Physiotherapy Total	Weight	Mean Difference IV, Random, 95% CI [VAS]
Arslan et al. (2001)	−3.6	1.3	10	−3.8	1	10	32.6%	0.20 [−0.82, 1.22]
Calis et al. (2006)	−1.2	3.3	25	−1.4	3.7	21	12.0%	0.20 [−1.84, 2.24]
Carette et al. (2003)	−3.9	2.8	25	−2.2	2.8	27	19.2%	−1.70 [−3.22, −0.18]
Dacre et al. (1989)	−4.5	7	22	−4.5	7	22	3.4%	0.00 [−4.14, 4.14]
Maryam et al. (2012)	−1.3	4	29	−2	1.2	29	19.3%	0.70 [−0.82, 2.22]
Ryans et al. (2005)	−1	1.9	20	−1.8	3.9	20	13.6%	0.80 [−1.10, 2.70]
van der Windt et al. (1998)	−3.2	2.6	52	−1.7	2.1	56	0.0%	−1.50 [−2.40, −0.60]
Total (95% CI)			131			129	100.0%	0.01 [−0.77, 0.78]

Heterogeneity: $Tau^2 = 0.21$; $Chi^2 = 6.46$, df = 5 ($P = 0.26$); $I^2 = 23\%$
Test for overall effect: $Z = 0.02$ ($P = 0.99$)

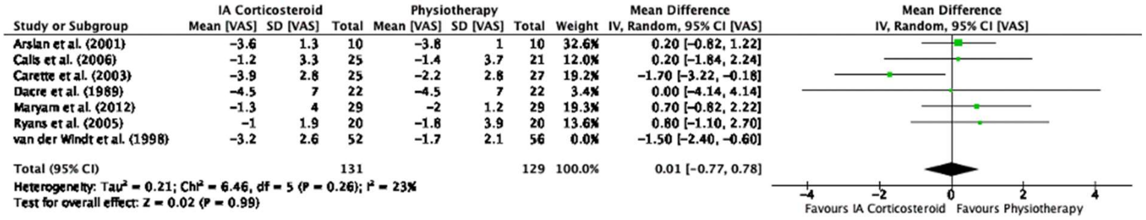

© 2020 Challoumas D et al. *JAMA Network Open.*

ER ROM

Study or Subgroup	IA Corticosteroid Mean [degrees]	IA Corticosteroid SD [degrees]	IA Corticosteroid Total	Physiotherapy Mean [degrees]	Physiotherapy SD [degrees]	Physiotherapy Total	Weight	Mean Difference IV, Random, 95% CI [degrees]
Arslan et al. (2001)	−13	14.1	10	−13.2	11.8	10	11.3%	0.20 [−11.20, 11.60]
Bulgen et al. (1984)	−8	14.1	11	−15	11.8	11	11.8%	7.00 [−3.87, 17.87]
Calis et al. (2006)	−7.5	11.5	25	−13.8	11.2	21	16.1%	6.30 [−0.28, 12.88]
Carette et al. (2003)	−18.3	16.3	25	−9.6	15.3	27	14.0%	−8.70 [−17.31, −0.09]
Maryam et al. (2012)	−9.8	10.7	31	−1.6	16.9	27	15.3%	−8.20 [−15.60, −0.80]
Ryans et al. (2005)	−14.3	15.2	20	−16.7	13.2	20	13.8%	2.40 [−6.42, 11.22]
van der Windt et al. (1998)	−6	14	52	3	12	56	17.8%	−9.00 [−13.94, −4.06]
Total (95% CI)			174			172	100.0%	−1.88 [−7.58, 3.82]

Heterogeneity: $Tau^2 = 41.15$; $Chi^2 = 21.91$, df = 6 ($P = 0.001$); $I^2 = 73\%$
Test for overall effect: Z = 0.65 (P = 0.52)

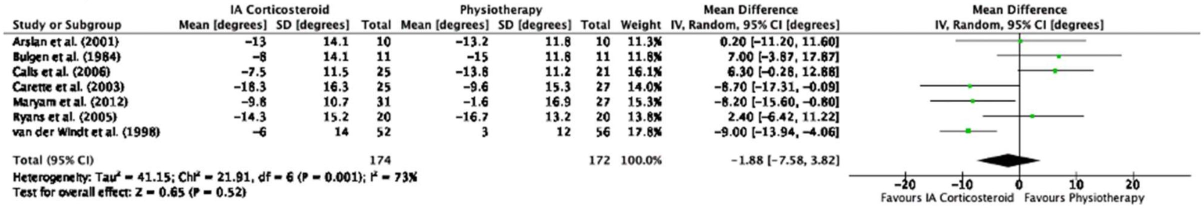

e) IA Corticosteroid + Physiotherapy vs IA Corticosteroid only

Pain

Study or Subgroup	IA Corticosteroid + Physiotherapy Mean [VAS]	IA Corticosteroid + Physiotherapy SD [VAS]	IA Corticosteroid + Physiotherapy Total	IA Corticosteroid Mean [VAS]	IA Corticosteroid SD [VAS]	IA Corticosteroid Total	Weight	Mean Difference IV, Fixed, 95% CI [VAS]
Carette et al. (2003)	−4.9	2.8	22	−3.9	2.8	25	13.2%	−1.00 [−2.60, 0.60]
Dacre et al. (1989)	−4.5	7	22	−4.5	7	22	2.0%	0.00 [−4.14, 4.14]
Kraal et al. (2018)	−6.8	3	11	−1.1	2.8	10	0.0%	−5.70 [−8.18, −3.22]
Maryam et al. (2012)	−1.6	1.4	30	−1.3	1.4	30	67.7%	−0.30 [−1.01, 0.41]
Ryans et al. (2005)	−1.6	2.6	20	−1	1.9	20	17.1%	−0.60 [−2.01, 0.81]
Total (95% CI)			94			97	100.0%	−0.44 [−1.02, 0.15]

Heterogeneity: $Chi^2 = 0.71$, df = 3 ($P = 0.87$); $I^2 = 0\%$
Test for overall effect: Z = 1.47 (P = 0.14)

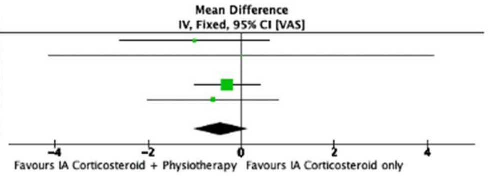

ER ROM

Study or Subgroup	IA Corticosteroid+Physiotherapy Mean [degrees]	IA Corticosteroid+Physiotherapy SD [degrees]	IA Corticosteroid+Physiotherapy Total	IA Corticosteroid only Mean [degrees]	IA Corticosteroid only SD [degrees]	IA Corticosteroid only Total	Weight	Mean Difference IV, Random, 95% CI [degrees]
Carette et al. (2003)	−26.5	16.9	22	−18.3	16.3	25	32.7%	−8.20 [−17.72, 1.32]
Kraal et al. (2018)	−32	13.7	11	−13	5.2	10	35.5%	−19.00 [−27.71, −10.29]
Maryam et al. (2012)	−3.8	12.3	29	−9.8	10.7	31	0.0%	6.00 [0.15, 11.85]
Ryans et al. (2005)	−21	16.5	20	−14.3	15.2	20	31.7%	−6.70 [−16.53, 3.13]
Total (95% CI)			53			55	100.0%	−11.56 [−19.39, −3.74]

Heterogeneity: $Tau^2 = 25.06$; $Chi^2 = 4.20$, df = 2 ($P = 0.12$); $I^2 = 52\%$
Test for overall effect: Z = 2.90 (P = 0.004)

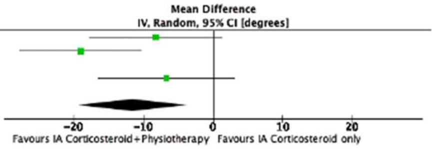

f) IA Corticosteroid + Physiotherapy vs Physiotherapy only

Pain

Study or Subgroup	IA Corticoteroid + Physiotherapy Mean [VAS]	IA Corticoteroid + Physiotherapy SD [VAS]	IA Corticoteroid + Physiotherapy Total	Physiotherapy only Mean [VAS]	Physiotherapy only SD [VAS]	Physiotherapy only Total	Weight	Mean Difference IV, Random, 95% CI [VAS]
Carette et al. (2003)	−4.9	2.8	22	−2.2	2.8	27	0.0%	−2.70 [−4.28, −1.12]
Dacre et al. (1989)	−4.5	7	22	−4.5	7	22	2.4%	0.00 [−4.14, 4.14]
Maryam et al. (2012)	−1.6	1.4	28	−2	1.2	28	87.9%	0.40 [−0.28, 1.08]
Ryans et al. (2005)	−1.6	2.6	20	−1.8	3.9	20	9.7%	0.20 [−1.85, 2.25]
Total (95% CI)			70			70	100.0%	0.37 [−0.27, 1.01]

Heterogeneity: $Tau^2 = 0.00$; $Chi^2 = 0.06$, df = 2 ($P = 0.97$); $I^2 = 0\%$
Test for overall effect: Z = 1.14 (P = 0.26)

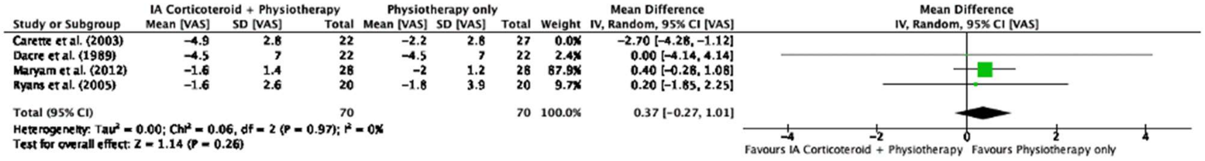

ER ROM

Study or Subgroup	Acupuncture + Physiotherapy Mean [degrees]	Acupuncture + Physiotherapy SD [degrees]	Acupuncture + Physiotherapy Total	Physiotherapy only Mean [degrees]	Physiotherapy only SD [degrees]	Physiotherapy only Total	Weight	Mean Difference IV, Random, 95% CI [degrees]
Koh et al. (2013)	−9.7	11	23	−8.8	13.6	23	59.9%	−0.90 [−8.05, 6.25]
Lo et al. (2020)	−15	21	11	−20	20	10	10.0%	5.00 [−12.54, 22.54]
Ma et al. (2006)	−10.4	16	15	−8.9	16.8	30	30.1%	−1.50 [−11.58, 8.58]
Total (95% CI)			49			63	100.0%	−0.49 [−6.03, 5.04]

Heterogeneity: $Tau^2 = 0.00$; $Chi^2 = 0.43$, df = 2 ($P = 0.81$); $I^2 = 0\%$
Test for overall effect: Z = 0.17 (P = 0.86)

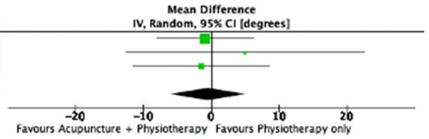

g) IA Corticosteroid vs SA Corticosteroid

Pain

Study or Subgroup	IA Corticosteroid Mean [VAS]	IA Corticosteroid SD [VAS]	IA Corticosteroid Total	SA Corticosteroid Mean [VAS]	SA Corticosteroid SD [VAS]	SA Corticosteroid Total	Weight	Mean Difference IV, Fixed, 95% CI [VAS]
Cho et al. (2016)	−8.1	1.4	42	−3.2	1.7	42	0.0%	−4.90 [−5.57, −4.23]
Oh et al. (2011)	−3.8	2.6	37	−3.6	2.5	34	19.8%	−0.20 [−1.39, 0.99]
Rizk et al. (1991)	0	2.3	16	0	2.5	16	10.1%	0.00 [−1.66, 1.66]
Shin & Lee (2013)	−5.5	2.3	48	−4.3	2.5	49	30.6%	−1.20 [−2.16, −0.24]
Sun et al. (2018)	−2.3	3.4	32	−0.2	3.4	32	10.1%	−2.10 [−3.77, −0.43]
Yoon et al. (2016)	−1	1.6	30	−1.2	2.2	30	29.5%	0.20 [−0.77, 1.17]
Total (95% CI)			163			161	100.0%	−0.56 [−1.09, −0.03]

Heterogeneity: $Chi^2 = 8.14$, df = 4 ($P = 0.09$); $I^2 = 51\%$
Test for overall effect: Z = 2.07 (P = 0.04)

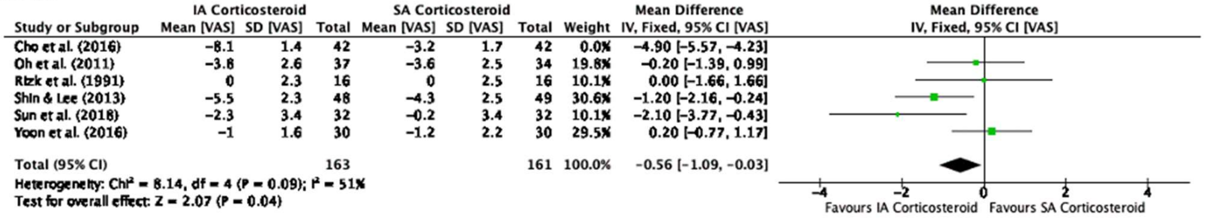

© 2020 Challoumas D et al. *JAMA Network Open.*

ER ROM

Study or Subgroup	IA Corticosteroid Mean [degrees]	SD [degrees]	Total	SA Corticosteroid Mean [degrees]	SD [degrees]	Total	Weight	Mean Difference IV, Random, 95% CI [degrees]
Cho et al. (2016)	−30.2	13.3	42	−21	13.4	42	25.3%	−9.20 [−14.91, −3.49]
Oh et al. (2011)	−20	23.7	37	−17	23.7	34	11.6%	−3.00 [−14.04, 8.04]
Rizk et al. (1991)	−0.5	21.8	16	−1	18.6	16	8.0%	0.50 [−13.54, 14.54]
Shin & Lee (2013)	−14	21.8	48	−10	18.6	49	17.7%	−4.00 [−12.07, 4.07]
Sun et al. (2018)	−15.9	41.9	32	−1.1	29.4	32	5.4%	−14.80 [−32.53, 2.93]
Yoon et al. (2016)	−10	8	30	−10	8	30	32.1%	0.00 [−4.05, 4.05]
Total (95% CI)			205			203	100.0%	−4.13 [−8.51, 0.24]

Heterogeneity: $Tau^2 = 11.23; Chi^2 = 8.58, df = 5 (P = 0.13); I^2 = 42\%$
Test for overall effect: $Z = 1.85 (P = 0.06)$

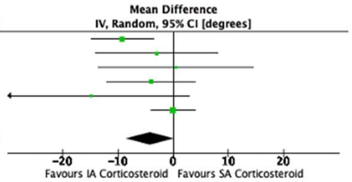

h) IA Corticosteroid + Arthrographic Distension vs IA Corticosteroid only

Pain

Study or Subgroup	IA Corticosteroid + Distension Mean [VAS]	SD [VAS]	Total	IA Corticosteroid Mean [VAS]	SD [VAS]	Total	Weight	Mean Difference IV, Random, 95% CI [VAS]
Gam et al. (1998)	0.5	2.8	12	0	3.6	8	1.8%	0.50 [−2.46, 3.46]
Lee et al. (2017a)	−4.7	1.6	32	−3.8	1.6	32	25.6%	−0.90 [−1.68, −0.12]
Park & Hwnag (2000)	−6.4	1.5	28	−5.6	1.7	27	21.9%	−0.80 [−1.65, 0.05]
Sharma et al. (2016)	−3.7	1.7	35	−3.1	1.8	35	23.4%	−0.60 [−1.42, 0.22]
Yoon et al. (2016)	−2.2	1.4	30	−1	1.6	30	27.2%	−1.20 [−1.96, −0.44]
Total (95% CI)			137			132	100.0%	−0.86 [−1.26, −0.47]

Heterogeneity: $Tau^2 = 0.00; Chi^2 = 2.00, df = 4 (P = 0.74); I^2 = 0\%$
Test for overall effect: $Z = 4.27 (P < 0.0001)$

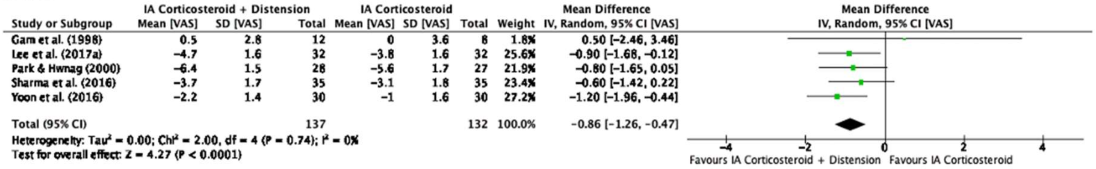

ER ROM

Study or Subgroup	IA Corticosteroid + Distension Mean [degrees]	SD [degrees]	Total	IA Corticosteroid only Mean [degrees]	SD [degrees]	Total	Weight	Mean Difference IV, Random, 95% CI [degrees]
Lee et al. (2017a)	−17	15.8	32	−15	13.1	32	22.1%	−2.00 [−9.11, 5.11]
Park & Hwnag (2000)	−25.7	19.9	28	−25.1	15.9	27	13.4%	−0.60 [−10.10, 8.90]
Sharma et al. (2016)	−10.4	16.8	35	−10.5	15.5	35	19.9%	0.10 [−7.47, 7.67]
Yoon et al. (2016)	−17	9.5	30	−10	8	30	44.7%	−7.00 [−11.44, −2.56]
Total (95% CI)			125			124	100.0%	−3.63 [−7.30, 0.04]

Heterogeneity: $Tau^2 = 2.70; Chi^2 = 3.67, df = 3 (P = 0.30); I^2 = 18\%$
Test for overall effect: $Z = 1.94 (P = 0.05)$

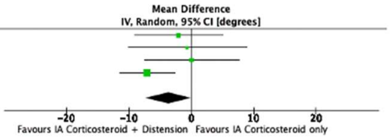

i) Acupuncture + Physiotherapy vs Physiotherapy only (+/- sham acupuncture)

Pain

Study or Subgroup	Acupuncture + Physiotherapy Mean [VAS]	SD [VAS]	Total	Physiotherapy only Mean [VAS]	SD [VAS]	Total	Weight	Mean Difference IV, Random, 95% CI [VAS]
Koh et al. (2013)	−2.1	1.7	23	−1.6	1.6	23	45.6%	−0.50 [−1.45, 0.45]
Lo et al. (2020)	−2.8	1.9	11	−3.5	1.6	10	18.6%	0.70 [−0.80, 2.20]
Ma et al. (2006)	−1.9	1.8	15	−1.4	1.6	30	35.9%	−0.50 [−1.58, 0.58]
Total (95% CI)			49			63	100.0%	−0.28 [−0.92, 0.37]

Heterogeneity: $Tau^2 = 0.00; Chi^2 = 2.01, df = 2 (P = 0.37); I^2 = 0\%$
Test for overall effect: $Z = 0.84 (P = 0.40)$

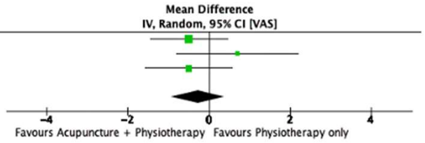

ER ROM

Study or Subgroup	Acupuncture + Physiotherapy Mean [VAS]	SD [VAS]	Total	Physiotherapy only Mean [VAS]	SD [VAS]	Total	Weight	Mean Difference IV, Random, 95% CI [VAS]
Koh et al. (2013)	−2.1	1.7	23	−1.6	1.6	23	45.6%	−0.50 [−1.45, 0.45]
Lo et al. (2020)	−2.8	1.9	11	−3.5	1.6	10	18.6%	0.70 [−0.80, 2.20]
Ma et al. (2006)	−1.9	1.8	15	−1.4	1.6	30	35.9%	−0.50 [−1.58, 0.58]
Total (95% CI)			49			63	100.0%	−0.28 [−0.92, 0.37]

Heterogeneity: $Tau^2 = 0.00; Chi^2 = 2.01, df = 2 (P = 0.37); I^2 = 0\%$
Test for overall effect: $Z = 0.84 (P = 0.40)$

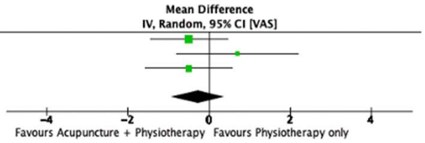

© 2020 Challoumas D et al. *JAMA Network Open.*

eFigure 2. Results of Pairwise Meta-analyses With Respective Mean Differences for Late Short-term Outcomes

Late short-term results (8-12 weeks)

a) IA Corticosteroid vs No Treatment/Placebo

Pain

Study or Subgroup	IA Corticosteroid Mean [VAS]	SD [VAS]	Total	No treatment/Placebo Mean [VAS]	SD [VAS]	Total	Weight	Mean Difference IV, Random, 95% CI [VAS]
Bal et al. (2008)	−7	1.4	40	−5.7	1.5	40	12.7%	−1.30 [−1.94, −0.66]
Calis et al. (2006)	−2.9	1.4	26	−1.9	1.5	22	11.0%	−1.00 [−1.83, −0.17]
Carette et al. (2003)	−4.8	1.5	25	−3	1.4	23	11.1%	−1.80 [−2.62, −0.98]
Dehghan et al. (2013)	−3.4	2.4	29	−2.9	2.4	28	7.8%	−0.50 [−1.75, 0.75]
Prestgaard et al. (2015)	−3.9	1.8	42	−2.7	1.8	40	11.4%	−1.20 [−1.98, −0.42]
Ranalletta et al. (2015)	−6.4	1.1	38	−6.5	1.1	36	13.8%	0.10 [−0.40, 0.60]
Rizk et al. (1991)	−0.5	1.4	16	−1.8	1.5	15	0.0%	1.30 [0.28, 2.32]
Roh et al. (2012)	−1.7	2	25	−1.2	2	25	8.8%	−0.50 [−1.61, 0.61]
Sharma et al. (2016)	−3.9	1.9	36	−1.9	2.1	36	10.2%	−2.00 [−2.93, −1.07]
Shin & Lee (2013)	−4.7	1.4	48	−3.6	1.5	49	13.2%	−1.10 [−1.68, −0.52]
Total (95% CI)			309			299	100.0%	−1.03 [−1.52, −0.54]

Heterogeneity: Tau² = 0.38; Chi² = 28.82, df = 8 (P = 0.0003); I² = 72%
Test for overall effect: Z = 4.15 (P < 0.0001)

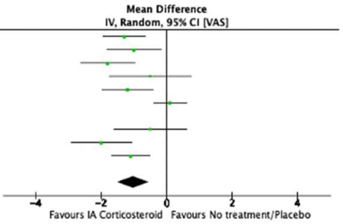

ER ROM

Study or Subgroup	IA Corticosteroid Mean [degrees]	SD [degrees]	Total	No Treatment/Placebo Mean [degrees]	SD [degrees]	Total	Weight	Mean Difference IV, Random, 95% CI [degrees]
Bal et al. (2008)	−40	37.1	40	−30	41.4	40	3.0%	−10.00 [−27.23, 7.23]
Bulgen et al. (1984)	−7	13.1	11	−7	13.4	8	5.4%	0.00 [−12.09, 12.09]
Calis et al. (2006)	−15.7	12.9	26	−9.3	12.1	20	10.6%	−6.40 [−13.66, 0.86]
Carette et al. (2003)	−25.5	17	25	−13.4	17	23	7.5%	−12.10 [−21.73, −2.47]
Dehghan et al. (2013)	−14	12	29	−13	9.7	28	13.4%	−1.00 [−6.66, 4.66]
Prestgaard et al. (2015)	−20.6	13.6	42	−8.1	15.3	40	12.2%	−12.50 [−18.78, −6.22]
Ranalletta et al. (2015)	−21.9	10.3	36	−21.6	10.5	38	0.0%	−0.30 [−5.04, 4.44]
Rizk et al. (1991)	−1.3	13.1	16	−3	13.4	16	8.0%	1.70 [−7.48, 10.88]
Roh et al. (2012)	−5	8.5	25	−2.5	8.5	25	15.3%	−2.50 [−7.21, 2.21]
Sharma et al. (2016)	−18.6	16.2	36	−6.7	15.9	36	10.3%	−11.90 [−19.31, −4.49]
Shin & Lee (2013)	−27	13.1	48	−20	13.4	49	14.2%	−7.00 [−12.27, −1.73]
Total (95% CI)			298			285	100.0%	−6.02 [−9.25, −2.80]

Heterogeneity: Tau² = 11.88; Chi² = 17.17, df = 9 (P = 0.05); I² = 48%
Test for overall effect: Z = 3.66 (P = 0.0003)

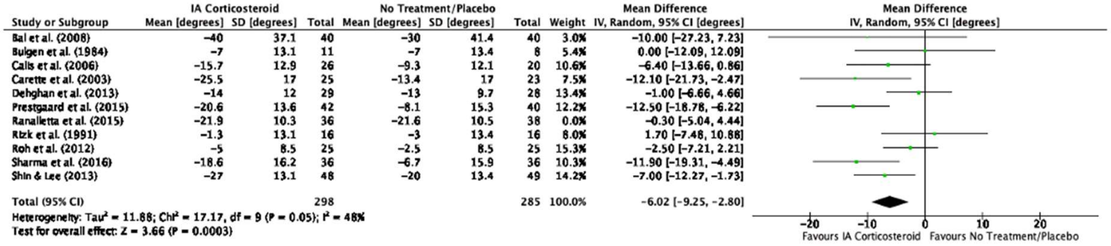

b) IA Corticosteroid vs Physiotherapy

Pain

Study or Subgroup	IA Corticosteroid Mean [VAS]	SD [VAS]	Total	Physiotherapy Mean [VAS]	SD [VAS]	Total	Weight	Mean Difference IV, Random, 95% CI [VAS]
Arslan et al. (2001)	−6.1	1.1	10	−5.9	1.8	10	18.4%	−0.20 [−1.51, 1.11]
Calis et al. (2006)	−2.9	1.8	26	−1.6	2.2	22	22.7%	−1.30 [−2.45, −0.15]
Carette et al. (2003)	−4.8	1.5	25	−3.8	1.6	27	36.0%	−1.00 [−1.84, −0.16]
van der Windt et al. (1998)	−6.6	2.8	53	−4.7	3.3	56	22.8%	−1.90 [−3.05, −0.75]
Total (95% CI)			114			115	100.0%	−1.13 [−1.74, −0.51]

Heterogeneity: Tau² = 0.09; Chi² = 3.85, df = 3 (P = 0.28); I² = 22%
Test for overall effect: Z = 3.59 (P = 0.0003)

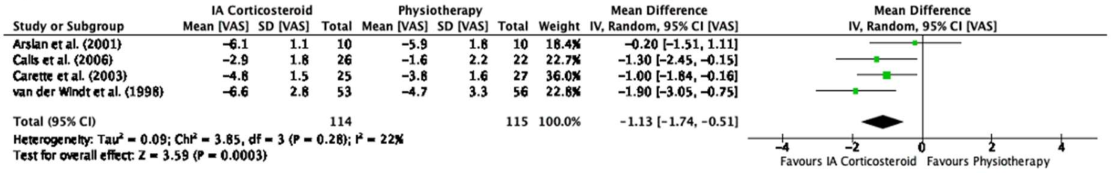

ER ROM

Study or Subgroup	IA Corticosteroid Mean [degrees]	SD [degrees]	Total	Physiotherapy Mean [degrees]	SD [degrees]	Total	Weight	Mean Difference IV, Random, 95% CI [degrees]
Arslan et al. (2001)	−29.1	14.4	10	−28.9	12.3	10	14.6%	−0.20 [−11.94, 11.54]
Bulgen et al. (1984)	−7	14.4	11	−17	12.3	11	0.0%	10.00 [−1.19, 21.19]
Calis et al. (2006)	−15.7	11.7	26	−9.3	8.6	22	60.6%	−6.40 [−12.16, −0.64]
Carette et al. (2003)	−25.5	17	25	−18	16	27	24.8%	−7.50 [−16.49, 1.49]
Total (95% CI)			61			59	100.0%	−5.77 [−10.25, −1.29]

Heterogeneity: Tau² = 0.00; Chi² = 1.05, df = 2 (P = 0.59); I² = 0%
Test for overall effect: Z = 2.52 (P = 0.01)

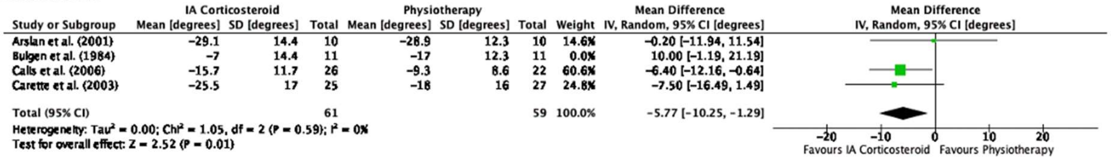

c) IA Corticosteroid vs SA Corticosteroid

Pain

Study or Subgroup	IA Corticosteroid Mean [VAS]	SD [VAS]	Total	SA Corticosteroid Mean [VAS]	SD [VAS]	Total	Weight	Mean Difference IV, Random, 95% CI [VAS]
Cho et al. (2016)	−5.7	1.7	42	−4.6	1.5	42	0.0%	−1.10 [−1.79, −0.41]
Khallaf et al. (2018)	−8.6	0.6	20	−8.3	0.7	20	43.1%	−0.30 [−0.70, 0.10]
Oh et al. (2011)	−4.5	2.5	37	−3.8	2.3	34	11.6%	−0.70 [−1.82, 0.42]
Rizk et al. (1991)	−0.5	2	16	−0.7	2.1	16	7.7%	0.20 [−1.22, 1.62]
Shin & Lee (2013)	−4.7	2	48	−5.3	2.1	49	19.1%	0.60 [−0.22, 1.42]
Sun et al. (2018)	−2.9	3.4	30	−1.8	4	34	4.9%	−1.10 [−2.91, 0.71]
Yoon et al. (2016)	−1.2	1.8	30	−1.4	2.2	30	13.6%	0.20 [−0.82, 1.22]
Total (95% CI)			181			183	100.0%	−0.11 [−0.52, 0.31]

Heterogeneity: Tau² = 0.06; Chi² = 6.45, df = 5 (P = 0.26); I² = 22%
Test for overall effect: Z = 0.51 (P = 0.61)

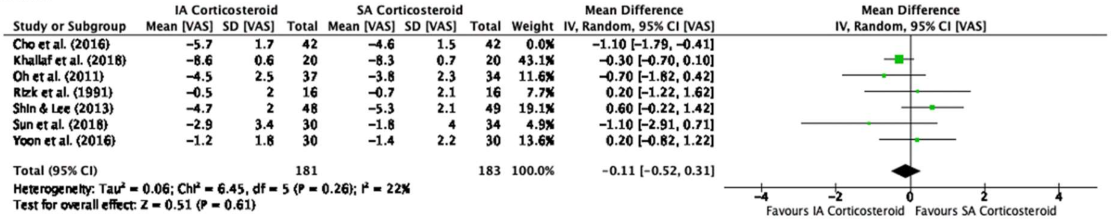

© 2020 Challoumas D et al. *JAMA Network Open.*

ER ROM

Study or Subgroup	IA Corticosteroid Mean [degrees]	IA Corticosteroid SD [degrees]	IA Corticosteroid Total	SA Corticosteroid Mean [degrees]	SA Corticosteroid SD [degrees]	SA Corticosteroid Total	Weight	Mean Difference IV, Random, 95% CI [degrees]
Cho et al. (2016)	−30	13.7	42	−22.1	14	42	28.3%	−7.90 [−13.82, −1.98]
Oh et al. (2011)	−20	30.5	37	−18	30.5	34	9.1%	−2.00 [−16.20, 12.20]
Rizk et al. (1991)	−1.3	23.5	16	−6.6	21.9	16	7.7%	5.30 [−10.44, 21.04]
Shin & Lee (2013)	−27	23.5	48	−19	21.9	49	17.8%	−8.00 [−17.04, 1.04]
Sun et al. (2018)	−25.1	43	32	5	31.1	32	0.0%	−30.10 [−48.49, −11.71]
Yoon et al. (2016)	−20	8	30	−20	8	30	37.1%	0.00 [−4.05, 4.05]
Total (95% CI)			173			171	100.0%	-3.44 [-8.16, 1.29]

Heterogeneity: $Tau^2 = 11.41$; $Chi^2 = 6.98$, df = 4 ($P = 0.14$); $I^2 = 43\%$

Test for overall effect: $Z = 1.42$ ($P = 0.15$)

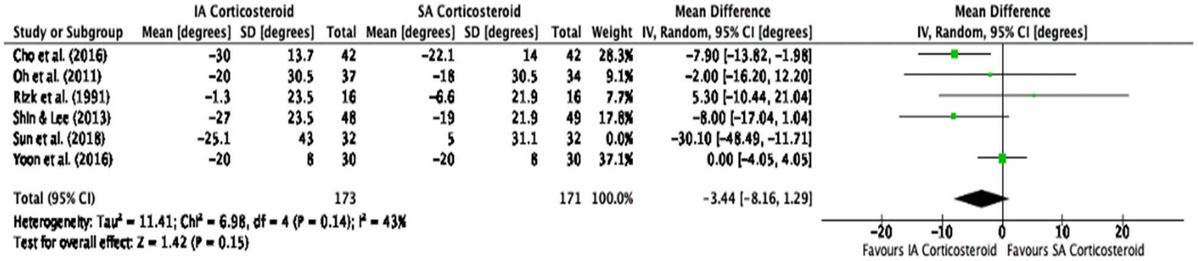

d) IA Corticosteroid + Arthrographic Distension vs IA Corticosteroid only

Pain

Study or Subgroup	Arthrographic Distension + IA Corticosteroid Mean [VAS]	Arthrographic Distension + IA Corticosteroid SD [VAS]	Arthrographic Distension + IA Corticosteroid Total	IA Corticosteroid only Mean [VAS]	IA Corticosteroid only SD [VAS]	IA Corticosteroid only Total	Weight	Mean Difference IV, Random, 95% CI [VAS]
Gam et al. (1998)	−2	1.6	11	−3	1.6	11	0.0%	1.00 [−0.34, 2.34]
Lee et al. (2017a)	−5	1.7	32	−4.3	1.6	32	14.1%	−0.70 [−1.51, 0.11]
Reza et al. (2013)	−4.7	0.7	50	−3.9	1.2	50	62.1%	−0.80 [−1.19, −0.41]
Sharma et al. (2016)	−4.3	2.3	35	−3.9	1.6	35	10.7%	−0.40 [−1.33, 0.53]
Yoon et al. (2016)	−2.4	1.5	30	−1.2	1.8	30	13.1%	−1.20 [−2.04, −0.36]
Total (95% CI)			147			147	100.0%	-0.80 [-1.10, -0.49]

Heterogeneity: $Tau^2 = 0.00$; $Chi^2 = 1.65$, df = 3 ($P = 0.65$); $I^2 = 0\%$

Test for overall effect: $Z = 5.14$ ($P < 0.00001$)

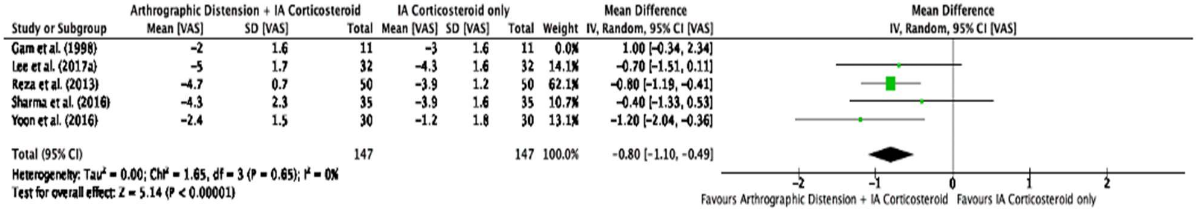

ER ROM

Study or Subgroup	IA Corticosteroid+Distension Mean [degrees]	IA Corticosteroid+Distension SD [degrees]	IA Corticosteroid+Distension Total	IA Corticosteroid only Mean [degrees]	IA Corticosteroid only SD [degrees]	IA Corticosteroid only Total	Weight	Mean Difference IV, Random, 95% CI [degrees]
Lee et al. (2017a)	−18.3	16	32	−16.6	12.4	32	16.1%	−1.70 [−8.71, 5.31]
Reza et al. (2013)	−30.4	15	50	−13.6	11.7	50	0.0%	−16.80 [−22.07, −11.53]
Sharma et al. (2016)	−17.5	16.2	35	−18.6	16.2	35	13.7%	1.10 [−6.49, 8.69]
Tveita et al. (2008)	−11	14	39	−10	11	37	24.8%	−1.00 [−6.65, 4.65]
Yoon et al. (2016)	−22.5	8.5	30	−20	8	30	45.4%	−2.50 [−6.68, 1.68]
Total (95% CI)			136			134	100.0%	-1.50 [-4.32, 1.31]

Heterogeneity: $Tau^2 = 0.00$; $Chi^2 = 0.70$, df = 3 ($P = 0.87$); $I^2 = 0\%$

Test for overall effect: $Z = 1.05$ ($P = 0.29$)

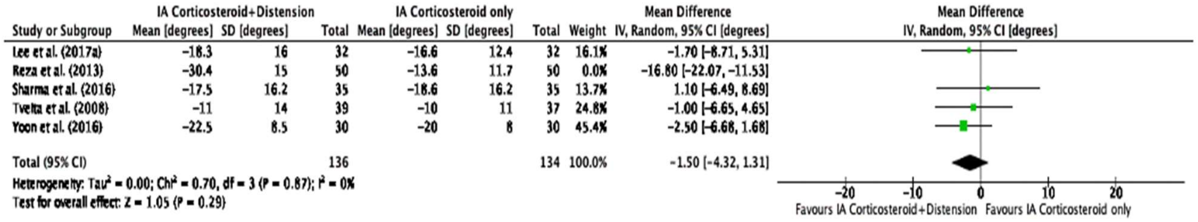

© 2020 Challoumas D et al. *JAMA Network Open.*

eFigure 3. Results of Pairwise Meta-analyses With Respective Mean Differences for Mid-term Outcomes

Mid-term results (4-6 months)

a) IA Corticosteroid vs No Treatment/Placebo

Pain

Study or Subgroup	IA Corticosteroid Mean [VAS]	SD [VAS]	Total	No Treatment/Placebo Mean [VAS]	SD [VAS]	Total	Weight	Mean Difference IV, Random, 95% CI [VAS]
Carette et al. (2003)	−5.1	2.7	25	−3.6	2.7	23	13.3%	−1.50 [−3.03, 0.03]
Dehghan et al. (2013)	−4	2.1	29	−3.6	2.2	28	18.8%	−0.40 [−1.52, 0.72]
Prestgaard et al. (2015)	−4.3	1.6	42	−2.9	1.3	42	0.0%	−1.40 [−2.02, −0.78]
Rizk et al. (1991)	−1	2.1	16	−2.1	2	16	14.6%	1.10 [−0.32, 2.52]
Roh et al. (2012)	−2.3	2.1	25	−1.9	2	25	18.5%	−0.40 [−1.54, 0.74]
Ryans et al. (2005)	−1	2.4	20	−2.5	3.4	20	10.5%	1.50 [−0.32, 3.32]
Shin & Lee (2013)	−5.1	2.1	48	−5.2	2	49	24.2%	0.10 [−0.72, 0.92]
Total (95% CI)			163			161	100.0%	−0.01 [−0.71, 0.70]

Heterogeneity: $Tau^2 = 0.36$; $Chi^2 = 9.61$, $df = 5$ ($P = 0.09$); $I^2 = 48\%$
Test for overall effect: $Z = 0.02$ ($P = 0.98$)

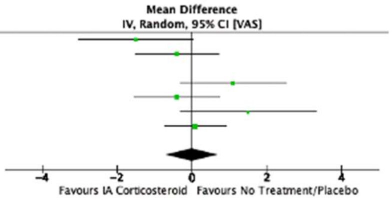

ER ROM

Study or Subgroup	IA Corticosteroid Mean [degrees]	SD [degrees]	Total	No Treatment/Placebo Mean [degrees]	SD [degrees]	Total	Weight	Mean Difference IV, Random, 95% CI [degrees]
Carette et al. (2003)	−29.5	17	25	−20.8	16.3	23	8.7%	−8.70 [−18.12, 0.72]
Dehghan et al. (2013)	−16.6	11.6	29	−17.5	9.7	28	18.8%	0.90 [−4.64, 6.44]
Prestgaard et al. (2015)	−20.9	17	42	−14.3	14.7	40	14.1%	−6.60 [−13.47, 0.27]
Rizk et al. (1991)	−6.6	14	16	−10.4	13	16	8.8%	3.80 [−5.56, 13.16]
Roh et al. (2012)	−9	8	25	−6	8	25	24.1%	−3.00 [−7.43, 1.43]
Ryans et al. (2005)	−19.1	19.2	20	−22.2	18.2	20	6.1%	3.10 [−8.49, 14.69]
Shin & Lee (2013)	−33	14	48	−27	13	49	19.5%	−6.00 [−11.38, −0.62]
Total (95% CI)			205			201	100.0%	−2.89 [−5.95, 0.18]

Heterogeneity: $Tau^2 = 5.06$; $Chi^2 = 8.65$, $df = 6$ ($P = 0.19$); $I^2 = 31\%$
Test for overall effect: $Z = 1.84$ ($P = 0.07$)

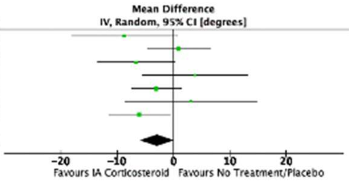

b) IA Corticosteroid vs Physiotherapy

Pain

Study or Subgroup	IA Corticosteroid Mean [VAS]	SD [VAS]	Total	Physiotherapy Mean [VAS]	SD [VAS]	Total	Weight	Mean Difference IV, Random, 95% CI [VAS]
Carette et al. (2003)	−5.1	2.7	25	−4.4	2.8	27	17.5%	−0.70 [−2.20, 0.80]
Dacre et al. (1989)	−4.2	2.4	22	−3.7	2.3	22	18.6%	−0.50 [−1.89, 0.89]
Maryam et al. (2012)	−1.5	1.4	31	−2.2	0.7	27	29.0%	0.70 [0.14, 1.26]
Ryans et al. (2005)	−1	2.5	20	−2.9	3.4	20	14.0%	1.90 [0.05, 3.75]
van der Windt et al. (1998)	−6.3	3.1	53	−5.4	3.3	56	20.9%	−0.90 [−2.10, 0.30]
Total (95% CI)			151			152	100.0%	0.07 [−0.85, 0.98]

Heterogeneity: $Tau^2 = 0.67$; $Chi^2 = 11.66$, $df = 4$ ($P = 0.02$); $I^2 = 66\%$
Test for overall effect: $Z = 0.14$ ($P = 0.89$)

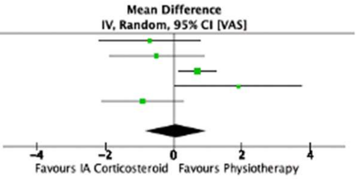

ER ROM

Study or Subgroup	IA Corticosteroid Mean [degrees]	SD [degrees]	Total	Physiotherapy Mean [degrees]	SD [degrees]	Total	Weight	Mean Difference IV, Random, 95% CI [degrees]
Carette et al. (2003)	−29.5	17	25	−23	16.6	27	18.9%	−6.50 [−15.64, 2.64]
Maryam et al. (2012)	−9.6	17.6	31	−5.6	5.6	27	37.0%	−4.00 [−10.55, 2.55]
Ryans et al. (2005)	−19.1	19.2	20	−18	14	20	14.6%	−1.10 [−11.51, 9.31]
van der Windt et al. (1998)	−13	18	53	−7	21	56	29.5%	−6.00 [−13.33, 1.33]
Total (95% CI)			129			130	100.0%	−4.64 [−8.62, −0.66]

Heterogeneity: $Tau^2 = 0.00$; $Chi^2 = 0.77$, $df = 3$ ($P = 0.86$); $I^2 = 0\%$
Test for overall effect: $Z = 2.28$ ($P = 0.02$)

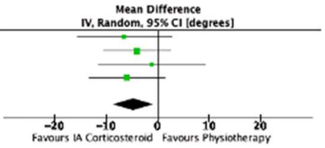

c) IA Corticosteroid + Physiotherapy vs IA Corticosteroid only

Pain

Study or Subgroup	IA Corticosteroid+Physiotherapy Mean [VAS]	SD [VAS]	Total	IA Corticosteroid only Mean [VAS]	SD [VAS]	Total	Weight	Mean Difference IV, Random, 95% CI [VAS]
Carette et al. (2003)	−5.3	2.8	25	−5.1	2.7	25	11.3%	−0.20 [−1.72, 1.32]
Dacre et al. (1989)	−3.3	2.1	22	−4.2	2	22	17.8%	0.90 [−0.31, 2.11]
Kraal et al. (2018)	−6	2.2	11	−5	1.9	10	8.5%	−1.00 [−2.75, 0.75]
Maryam et al. (2012)	−1.6	1.4	30	−1.5	1.4	30	51.0%	−0.10 [−0.81, 0.61]
Ryans et al. (2005)	−1.6	2.4	20	−1	2.5	20	11.4%	−0.60 [−2.12, 0.92]
Total (95% CI)			108			107	100.0%	−0.07 [−0.58, 0.45]

Heterogeneity: $Tau^2 = 0.00$; $Chi^2 = 4.04$, $df = 4$ ($P = 0.40$); $I^2 = 1\%$
Test for overall effect: $Z = 0.26$ ($P = 0.80$)

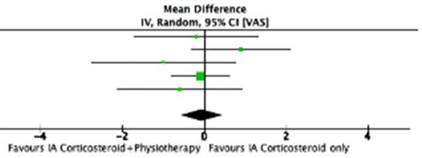

ER ROM

Study or Subgroup	IA Corticosteroid+Physiotherapy Mean [degrees]	SD [degrees]	Total	IA Corticosteroid only Mean [degrees]	SD [degrees]	Total	Weight	Mean Difference IV, Random, 95% CI [degrees]
Carette et al. (2003)	−34.1	16.9	22	−29.5	17	25	26.5%	−4.60 [−14.31, 5.11]
Kraal et al. (2018)	−42	12.9	10	−30	16.5	10	18.8%	−12.00 [−24.98, 0.98]
Maryam et al. (2012)	−5	11	29	−9.6	17.6	31	34.0%	4.60 [−2.78, 11.98]
Ryans et al. (2005)	−19.7	19.7	20	−19.1	19.2	20	20.7%	−0.60 [−12.66, 11.46]
Total (95% CI)			81			86	100.0%	−2.03 [−8.95, 4.88]

Heterogeneity: $Tau^2 = 22.44$; $Chi^2 = 5.50$, $df = 3$ ($P = 0.14$); $I^2 = 45\%$
Test for overall effect: $Z = 0.58$ ($P = 0.56$)

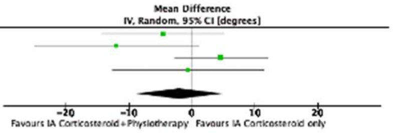

© 2020 Challoumas D et al. *JAMA Network Open.*

d) IA Corticosteroid + Physiotherapy vs Physiotherapy only

Pain

Study or Subgroup	IA Corticosteroid + Physiotherapy Mean [VAS]	SD [VAS]	Total	Physiotherapy only Mean [VAS]	SD [VAS]	Total	Weight	Mean Difference IV, Random, 95% CI [VAS]
Carette et al. (2003)	−5.3	2.8	22	−4.4	2.8	27	13.6%	−0.90 [−2.48, 0.68]
Dacre et al. (1989)	−3.3	2.1	22	−3.7	1.8	22	22.5%	0.40 [−0.76, 1.56]
Maryam et al. (2012)	−1.6	1.4	29	−2.2	0.7	27	53.4%	0.60 [0.03, 1.17]
Ryans et al. (2005)	−1.6	2.4	20	−2.9	3.4	20	10.5%	1.30 [−0.52, 3.12]
Total (95% CI)			93			96	100.0%	0.43 [−0.20, 1.05]

Heterogeneity: $Tau^2 = 0.10$; $Chi^2 = 3.91$, df = 3 (P = 0.27); $I^2 = 23\%$
Test for overall effect: Z = 1.33 (P = 0.18)

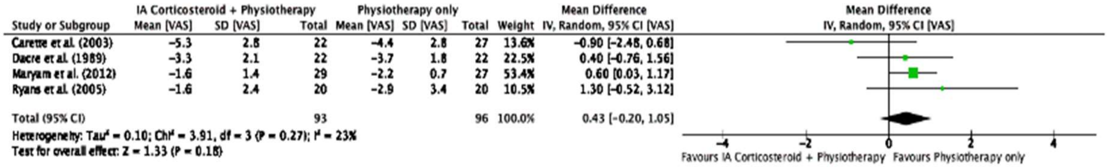

ER ROM

Study or Subgroup	IA Corticosteroid + Physiotherapy Mean [degrees]	SD [degrees]	Total	Physiotherapy only Mean [degrees]	SD [degrees]	Total	Weight	Mean Difference IV, Random, 95% CI [degrees]
Carette et al. (2003)	−34.1	3.6	22	−23	3.2	27	38.4%	−11.10 [−13.03, −9.17]
Maryam et al. (2012)	−5	11	29	−5.6	5.8	27	35.7%	0.60 [−3.93, 5.13]
Ryans et al. (2005)	−19.7	19.7	20	−18	14	20	25.9%	−1.70 [−12.29, 8.89]
Total (95% CI)			71			74	100.0%	−4.49 [−13.78, 4.81]

Heterogeneity: $Tau^2 = 57.68$; $Chi^2 = 23.65$, df = 2 (P < 0.00001); $I^2 = 92\%$
Test for overall effect: Z = 0.95 (P = 0.34)

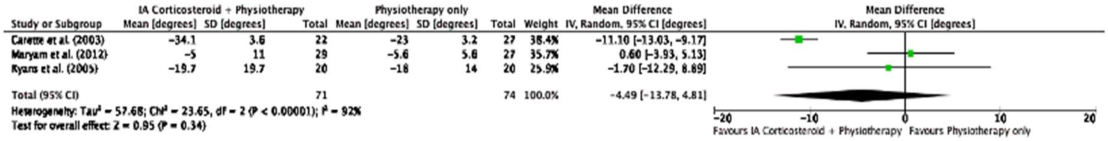

e) IA Corticosteroid vs SA Corticosteroid

Pain

Study or Subgroup	IA Corticosteroid Mean [VAS]	SD [VAS]	Total	SA Corticosteroid Mean [VAS]	SD [VAS]	Total	Weight	Mean Difference IV, Random, 95% CI [VAS]
Rizk et al. (1991)	−1	1.6	16	−1.4	2.1	16	28.7%	0.40 [−0.89, 1.69]
Shin & Lee (2013)	−5.1	1.6	48	−5.8	2.1	49	37.2%	0.70 [−0.04, 1.44]
Yoon et al. (2016)	−3.7	1.6	30	−2.6	2.1	30	34.1%	−1.10 [−2.04, −0.16]
Total (95% CI)			94			95	100.0%	−0.00 [−1.18, 1.18]

Heterogeneity: $Tau^2 = 0.84$; $Chi^2 = 8.91$, df = 2 (P = 0.01); $I^2 = 78\%$
Test for overall effect: Z = 0.00 (P = 1.00)

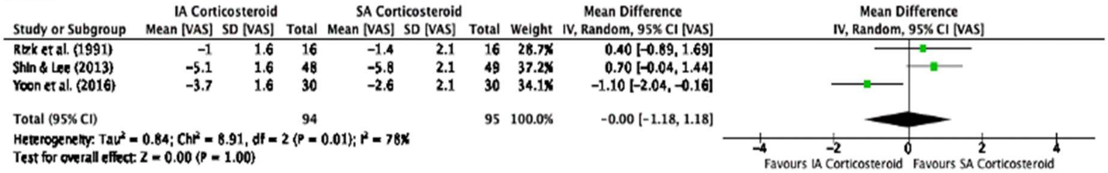

ER ROM

Study or Subgroup	IA Corticosteroid Mean [degrees]	SD [degrees]	Total	SA Corticosteroid Mean [degrees]	SD [degrees]	Total	Weight	Mean Difference IV, Random, 95% CI [degrees]
Rizk et al. (1991)	−6.6	23.5	16	−10.9	21.9	16	10.8%	4.30 [−11.44, 20.04]
Shin & Lee (2013)	−33	23.5	48	−26	21.9	49	26.9%	−7.00 [−16.04, 2.04]
Yoon et al. (2016)	−25	9	30	−26	8	30	62.2%	1.00 [−3.31, 5.31]
Total (95% CI)			94			95	100.0%	−0.80 [−6.28, 4.68]

Heterogeneity: $Tau^2 = 7.73$; $Chi^2 = 2.78$, df = 2 (P = 0.25); $I^2 = 28\%$
Test for overall effect: Z = 0.29 (P = 0.78)

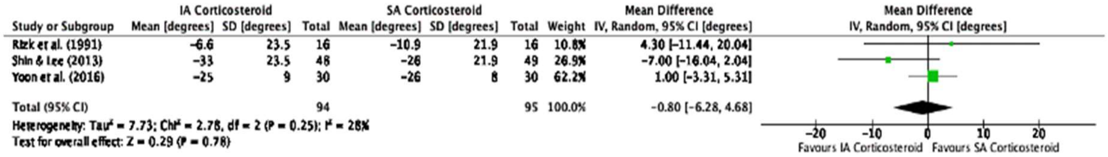

© 2020 Challoumas D et al. *JAMA Network Open.*

eFigure 4. Results of Pairwise Meta-analyses With Respective Mean Differences for Function

Function Early short-term results (2-6 weeks)

a) IA Corticosteroid vs No Treatment/Placebo

Study or Subgroup	IA Corticosteroid Mean	IA Corticosteroid SD	IA Corticosteroid Total	No Treatment/Placebo Mean	No Treatment/Placebo SD	No Treatment/Placebo Total	Weight	Std. Mean Difference IV, Random, 95% CI
Bal et al. (2008)	−29.5	36.8	40	−28	31.8	40	13.8%	−0.04 [−0.48, 0.40]
Calis et al. (2006)	−11.9	10.7	26	−6.7	11.8	20	11.0%	−0.46 [−1.05, 0.13]
Carette et al. (2003)	−36.7	25.5	25	−18.9	25.5	23	11.2%	−0.69 [−1.27, −0.10]
Prestgaard et al. (2015)	−35.2	20.3	42	−20.3	21.2	40	13.6%	−0.71 [−1.16, −0.26]
Ranalletta et al. (2015)	−39.3	4.7	36	−15	3.3	38	0.0%	−5.95 [−7.04, −4.86]
Roh et al. (2012)	−6	33	35	−2	33	35	13.2%	−0.12 [−0.59, 0.35]
Ryans et al. (2005)	−27.5	28.8	20	−14	15.3	20	10.3%	−0.57 [−1.21, 0.06]
Sharma et al. (2016)	−29.7	18.7	35	−10	20.6	37	12.8%	−0.99 [−1.48, −0.50]
Shin & Lee (2013)	−42.5	21.5	48	−18.2	20.8	49	14.0%	−1.14 [−1.57, −0.71]
Total (95% CI)			271			264	100.0%	−0.59 [−0.89, −0.30]

Heterogeneity: $Tau^2 = 0.11$; $Chi^2 = 19.23$, df = 7 (P = 0.007); $I^2 = 64\%$
Test for overall effect: Z = 3.94 (P < 0.0001)

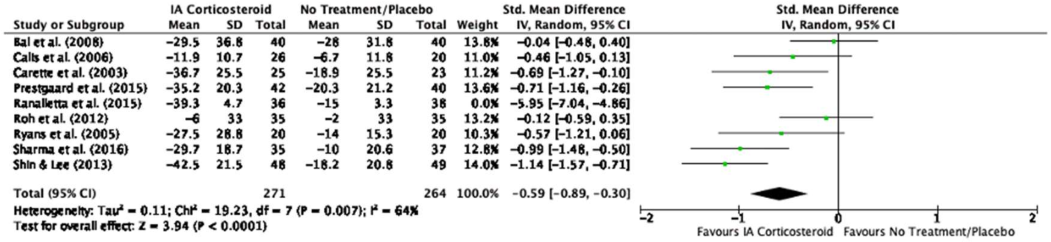

b) IA Corticosteroid vs Physiotherapy

Study or Subgroup	IA Corticosteroid Mean	IA Corticosteroid SD	IA Corticosteroid Total	Physiotherapy Mean	Physiotherapy SD	Physiotherapy Total	Weight	Std. Mean Difference IV, Random, 95% CI
Calis et al. (2006)	−11.9	10.7	20	−14.9	10.5	26	0.0%	3.00 [−3.19, 9.19]
Carette et al. (2003)	−36.7	25.5	25	−22.2	24.9	27	19.8%	−0.57 [−1.12, −0.01]
Maryam et al. (2012)	−19.7	44.1	31	−9.8	43.8	27	22.8%	−0.22 [−0.74, 0.30]
Ryans et al. (2005)	−27.5	28.8	20	−15.8	22.1	20	15.5%	−0.45 [−1.08, 0.18]
van der Windt et al. (1998)	−19	27	53	−6	22	56	41.9%	−0.53 [−0.91, −0.14]
Total (95% CI)			129			130	100.0%	−0.45 [−0.70, −0.20]

Heterogeneity: $Tau^2 = 0.00$; $Chi^2 = 1.06$, df = 3 (P = 0.79); $I^2 = 0\%$
Test for overall effect: Z = 3.58 (P = 0.0003)

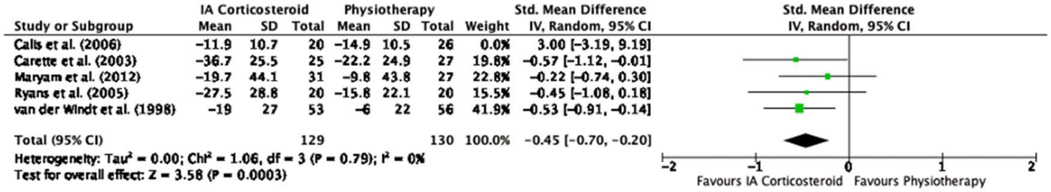

c) IA Corticosteroid + Physiotherapy vs IA Corticosteroid only

Study or Subgroup	IA Coticosteroid+Physiotherapy Mean	IA Coticosteroid+Physiotherapy SD	IA Coticosteroid+Physiotherapy Total	IA Corticosteroid only Mean	IA Corticosteroid only SD	IA Corticosteroid only Total	Weight	Std. Mean Difference IV, Random, 95% CI
Carette et al. (2003)	−46.5	24.9	22	−36.7	25.5	25	31.6%	−0.38 [−0.96, 0.20]
Kraal et al. (2018)	−68	29.6	11	−11	27.8	10	0.0%	−57.00 [−81.55, −32.45]
Maryam et al. (2012)	−26.9	43	29	−19.7	44.1	31	41.1%	−0.16 [−0.67, 0.34]
Ryans et al. (2005)	−35.1	25.7	20	−27.5	28.8	20	27.3%	−0.27 [−0.90, 0.35]
Total (95% CI)			71			76	100.0%	−0.26 [−0.59, 0.06]

Heterogeneity: $Tau^2 = 0.00$; $Chi^2 = 0.31$, df = 2 (P = 0.86); $I^2 = 0\%$
Test for overall effect: Z = 1.58 (P = 0.11)

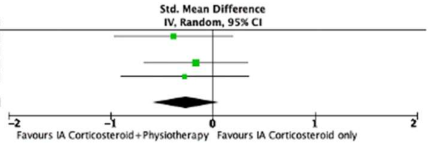

d) IA Corticosteroid + Physiotherapy vs Physiotherapy only

Study or Subgroup	IA Corticosteroid+Physiotherapy Mean	IA Corticosteroid+Physiotherapy SD	IA Corticosteroid+Physiotherapy Total	Physiotherapy only Mean	Physiotherapy only SD	Physiotherapy only Total	Weight	Std. Mean Difference IV, Random, 95% CI
Carette et al. (2003)	−46.6	24.9	22	−22.2	24.9	27	32.1%	−0.96 [−1.56, −0.37]
Maryam et al. (2012)	−26.9	43	29	−9.8	43.8	27	40.2%	−0.39 [−0.92, 0.14]
Ryans et al. (2005)	−35.1	25.7	20	−15.8	22.1	20	27.7%	−0.79 [−1.44, −0.14]
Total (95% CI)			71			74	100.0%	−0.68 [−1.03, −0.33]

Heterogeneity: $Tau^2 = 0.01$; $Chi^2 = 2.14$, df = 2 (P = 0.34); $I^2 = 7\%$
Test for overall effect: Z = 3.83 (P = 0.0001)

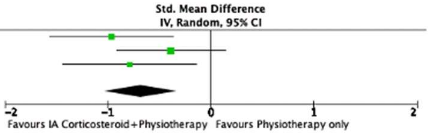

e) IA Corticosteroid vs SA Corticosteroid

Study or Subgroup	IA Corticosteroid Mean	IA Corticosteroid SD	IA Corticosteroid Total	SA Corticosteroid Mean	SA Corticosteroid SD	SA Corticosteroid Total	Weight	Std. Mean Difference IV, Random, 95% CI
Cho et al. (2016)	−52.4	13.2	42	−36.5	16.2	42	0.0%	−15.90 [−22.22, −9.58]
Oh et al. (2011)	−20	18.2	37	−16	18.2	34	24.2%	−0.22 [−0.68, 0.25]
Shin & Lee (2013)	−42.5	21.5	48	−37.6	24.5	49	33.2%	−0.21 [−0.61, 0.19]
Sun et al. (2018)	−10.6	27.7	32	−13.8	20.4	32	22.0%	0.13 [−0.36, 0.62]
Yoon et al. (2016)	−8.8	18.5	30	−9.4	20.2	30	20.6%	0.03 [−0.48, 0.54]
Total (95% CI)			147			145	100.0%	−0.09 [−0.32, 0.14]

Heterogeneity: $Tau^2 = 0.00$; $Chi^2 = 1.63$, df = 3 (P = 0.65); $I^2 = 0\%$
Test for overall effect: Z = 0.75 (P = 0.45)

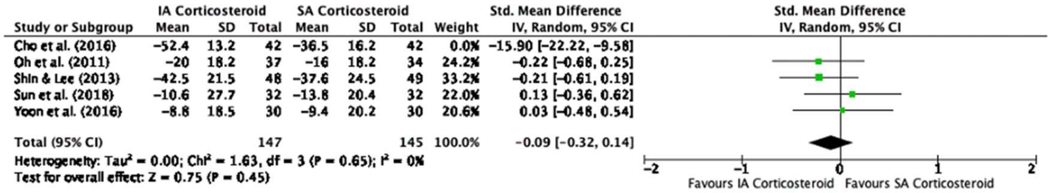

f) IA Corticosteroid + Arthrographic Distension vs IA Corticosteroid only

Study or Subgroup	IA Corticosteroid + Distension Mean	IA Corticosteroid + Distension SD	IA Corticosteroid + Distension Total	IA Corticosteroid only Mean	IA Corticosteroid only SD	IA Corticosteroid only Total	Weight	Std. Mean Difference IV, Random, 95% CI
Lee et al. (2017a)	−32.5	11.1	32	−24.2	11.8	32	32.9%	−0.72 [−1.22, −0.21]
Sharma et al. (2016)	−29.6	18.9	35	−29.7	18.7	35	34.9%	0.01 [−0.46, 0.47]
Yoon et al. (2016)	−20.7	18.4	30	−8.8	18.5	30	32.2%	−0.64 [−1.16, −0.12]
Total (95% CI)			97			97	100.0%	−0.44 [−0.90, 0.02]

Heterogeneity: $Tau^2 = 0.10$; $Chi^2 = 5.14$, df = 2 (P = 0.08); $I^2 = 61\%$
Test for overall effect: Z = 1.87 (P = 0.06)

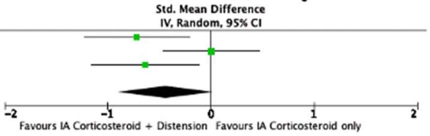

© 2020 Challoumas D et al. *JAMA Network Open.*

Function Late short-term results (8-12 weeks)

a) IA Corticosteroid vs No Treatment/Placebo

Study or Subgroup	IA Corticosteroid Mean	SD	Total	No Treatment/Placebo Mean	SD	Total	Weight	Std. Mean Difference IV, Random, 95% CI
Bal et al. (2008)	−58.5	31.9	40	−56	26.4	40	15.7%	−0.08 [−0.52, 0.35]
Calis et al. (2006)	−15.7	9.8	26	−10	11.5	20	11.6%	−0.53 [−1.12, 0.06]
Carette et al. (2003)	−45.5	25.5	25	−29.4	24.5	23	11.9%	−0.63 [−1.21, −0.05]
Prestgaard et al. (2015)	−46.2	19.9	42	−27.7	20.7	40	15.2%	−0.90 [−1.36, −0.45]
Ranalletta et al. (2015)	−38.2	3.6	36	−36	3.6	38	0.0%	−2.20 [−3.84, −0.56]
Roh et al. (2012)	−15	33	35	−5	33	35	14.8%	−0.30 [−0.77, 0.17]
Sharma et al. (2016)	−40	19	35	−17.5	21.3	37	14.0%	−1.10 [−1.60, −0.60]
Shin & Lee (2013)	−43.8	18	48	−33.1	23.6	49	16.8%	−0.51 [−0.91, −0.10]
Total (95% CI)			251			244	100.0%	−0.57 [−0.84, −0.31]

Heterogeneity: $Tau^2 = 0.07$; $Chi^2 = 12.54$, df = 6 ($P = 0.05$); $I^2 = 52\%$
Test for overall effect: $Z = 4.22$ ($P < 0.0001$)

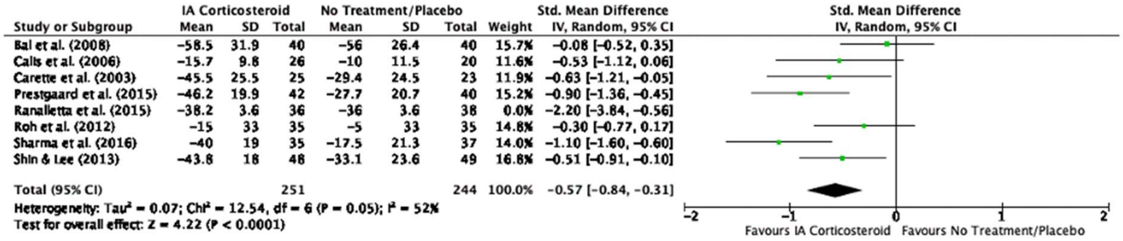

b) IA Corticosteroid vs Physiotherapy

Study or Subgroup	IA Corticosteroid Mean	SD	Total	Physiotherapy Mean	SD	Total	Weight	Std. Mean Difference IV, Random, 95% CI
Calis et al. (2006)	−15.7	9.8	20	−20.8	10.1	26	29.3%	0.50 [−0.09, 1.10]
Carette et al. (2003)	−45.5	25.5	25	−37.9	24.9	27	31.3%	−0.30 [−0.84, 0.25]
van der Windt et al. (1998)	−38	31	53	−28	32	56	39.5%	−0.32 [−0.69, 0.06]
Total (95% CI)			98			109	100.0%	−0.07 [−0.56, 0.42]

Heterogeneity: $Tau^2 = 0.12$; $Chi^2 = 5.65$, df = 2 ($P = 0.06$); $I^2 = 65\%$
Test for overall effect: $Z = 0.28$ ($P = 0.78$)

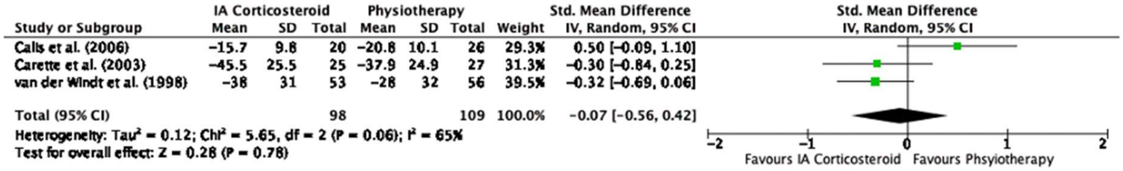

c) IA Corticosteroid vs SA Corticosteroid

Study or Subgroup	IA Corticosteroid Mean	SD	Total	SA Corticosteroid Mean	SD	Total	Weight	Std. Mean Difference IV, Random, 95% CI
Cho et al. (2016)	−51.8	14.2	42	−39.2	15.7	42	0.0%	−12.60 [−19.00, −6.20]
Khallaf et al. (2018)	−78.9	6	20	−75.2	5.3	20	14.5%	−0.64 [−1.28, −0.00]
Oh et al. (2011)	−18	18.2	37	−17	18.2	34	21.5%	−0.05 [−0.52, 0.41]
Shin & Lee (2013)	−43.8	18	48	−43.1	25.9	49	25.2%	−0.03 [−0.43, 0.37]
Sun et al. (2018)	−25.5	27.2	32	−6.2	23.2	32	19.4%	−0.75 [−1.26, −0.25]
Yoon et al. (2016)	−15.1	17.6	30	−10	16.8	30	19.4%	−0.29 [−0.80, 0.22]
Total (95% CI)			167			165	100.0%	−0.32 [−0.61, −0.02]

Heterogeneity: $Tau^2 = 0.05$; $Chi^2 = 6.98$, df = 4 ($P = 0.14$); $I^2 = 43\%$
Test for overall effect: $Z = 2.12$ ($P = 0.03$)

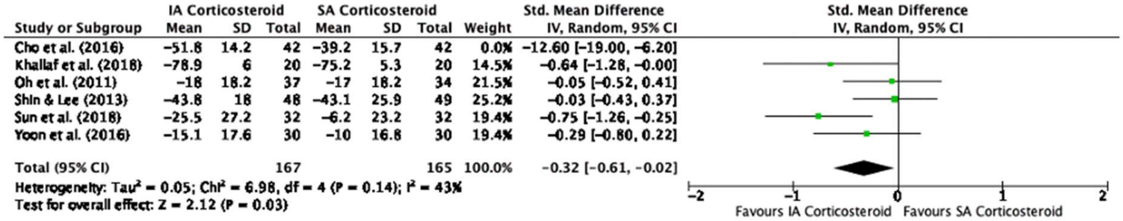

d) IA Corticosteroid + Arthrographic Distension vs IA Corticosteroid only

Study or Subgroup	IA Corticosteroid + Distension Mean	SD	Total	IA Corticosteroid only Mean	SD	Total	Weight	Std. Mean Difference IV, Random, 95% CI
Lee et al. (2017a)	−35.4	10.4	32	−36.2	10.6	32	23.7%	0.08 [−0.41, 0.57]
Sharma et al. (2016)	−40.4	17.6	35	−40	19	35	26.0%	−0.02 [−0.49, 0.45]
Tveita et al. (2008)	−39	21	39	−38	22	37	28.2%	−0.05 [−0.50, 0.40]
Yoon et al. (2016)	−19.9	19.1	30	−15.1	17.6	30	22.1%	−0.26 [−0.77, 0.25]
Total (95% CI)			136			134	100.0%	−0.06 [−0.30, 0.18]

Heterogeneity: $Tau^2 = 0.00$; $Chi^2 = 0.90$, df = 3 ($P = 0.82$); $I^2 = 0\%$
Test for overall effect: $Z = 0.47$ ($P = 0.64$)

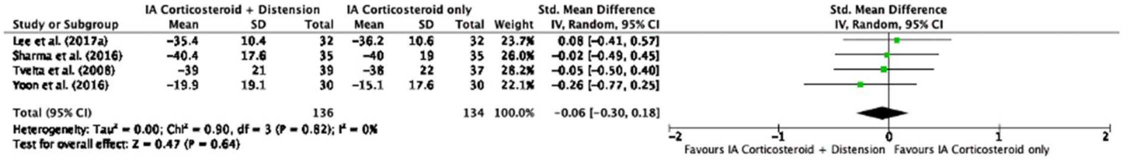

© 2020 Challoumas D et al. *JAMA Network Open.*

Function mid-term results (4-6 months)

a) IA Corticosteroid vs No Treatment/Placebo

Study or Subgroup	IA Corticosteroid Mean	IA Corticosteroid SD	IA Corticosteroid Total	No Treatment/Placebo Mean	No Treatment/Placebo SD	No Treatment/Placebo Total	Weight	Std. Mean Difference IV, Random, 95% CI
Carette et al. (2003)	−51.3	25.5	25	−38.4	24.5	23	13.9%	−0.51 [−1.08, 0.07]
Prestgaard et al. (2015)	−51.9	20.4	42	−43.2	21.2	40	24.1%	−0.41 [−0.85, 0.02]
Roh et al. (2012)	−22	33	35	−14	33	35	20.9%	−0.24 [−0.71, 0.23]
Ryans et al. (2005)	−35.1	26.6	20	−29.7	24.3	20	12.0%	−0.21 [−0.83, 0.41]
Shin & Lee (2013)	−48.5	15.2	48	−46.4	18.2	49	29.1%	−0.12 [−0.52, 0.27]
Total (95% CI)			170			167	100.0%	−0.28 [−0.50, −0.07]

Heterogeneity: $Tau^2 = 0.00$; $Chi^2 = 1.63$, df = 4 (P = 0.80); $I^2 = 0\%$
Test for overall effect: Z = 2.57 (P = 0.01)

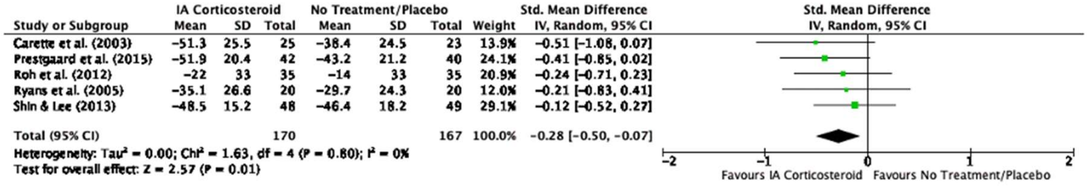

a) IA Corticosteroid vs Physiotherapy

Study or Subgroup	IA Corticosteroid Mean	IA Corticosteroid SD	IA Corticosteroid Total	Physiotherapy Mean	Physiotherapy SD	Physiotherapy Total	Weight	Std. Mean Difference IV, Random, 95% CI
Carette et al. (2003)	−51.3	25.5	25	−43.1	24.9	27	21.4%	−0.32 [−0.87, 0.23]
Maryam et al. (2012)	−22.3	42.5	31	−28.5	40.6	27	23.6%	0.15 [−0.37, 0.66]
Ryans et al. (2005)	−35.1	26.6	20	−25.2	26.1	20	17.0%	−0.37 [−0.99, 0.26]
van der Windt et al. (1998)	−45	30	53	−30	34	56	38.1%	−0.46 [−0.84, −0.08]
Total (95% CI)			129			130	100.0%	−0.27 [−0.55, 0.00]

Heterogeneity: $Tau^2 = 0.01$; $Chi^2 = 3.61$, df = 3 (P = 0.31); $I^2 = 17\%$
Test for overall effect: Z = 1.95 (P = 0.05)

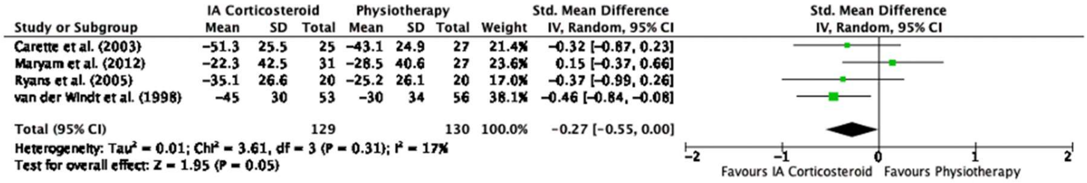

a) IA Corticosteroid + Physiotherapy vs IA Corticosteroid only

Study or Subgroup	IA Corticosteroid+Physiotherapy Mean	IA Corticosteroid+Physiotherapy SD	IA Corticosteroid+Physiotherapy Total	IA Corticosteroid only Mean	IA Corticosteroid only SD	IA Corticosteroid only Total	Weight	Std. Mean Difference IV, Random, 95% CI
Carette et al. (2003)	−52.5	24.9	22	−51.3	25.5	25	27.9%	−0.05 [−0.62, 0.53]
Kraal et al. (2018)	−73	27.4	11	−68	15.6	10	12.4%	−0.21 [−1.07, 0.65]
Maryam et al. (2012)	−22.6	46.9	29	−22.3	42.5	31	35.8%	−0.01 [−0.51, 0.50]
Ryans et al. (2005)	−34.2	26.1	20	−35.1	26.6	20	23.9%	0.03 [−0.59, 0.65]
Total (95% CI)			82			86	100.0%	−0.03 [−0.34, 0.27]

Heterogeneity: $Tau^2 = 0.00$; $Chi^2 = 0.22$, df = 3 (P = 0.97); $I^2 = 0\%$
Test for overall effect: Z = 0.22 (P = 0.83)

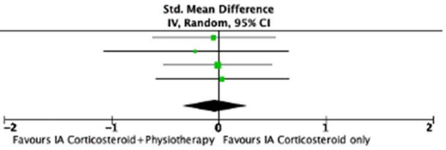

a) IA Corticosteroid + Physiotherapy vs Physiotherapy only

Study or Subgroup	IA Corticosteroid+Physiotherapy Mean	IA Corticosteroid+Physiotherapy SD	IA Corticosteroid+Physiotherapy Total	Physiotherapy only Mean	Physiotherapy only SD	Physiotherapy only Total	Weight	Std. Mean Difference IV, Random, 95% CI
Carette et al. (2003)	−52.5	24.9	22	−43.1	24.9	27	33.4%	−0.37 [−0.94, 0.20]
Maryam et al. (2012)	−22.6	46.9	29	−28.5	40.6	27	39.0%	0.13 [−0.39, 0.66]
Ryans et al. (2005)	−34.2	26.1	20	−25.2	26.1	20	27.7%	−0.34 [−0.96, 0.29]
Total (95% CI)			71			74	100.0%	−0.17 [−0.50, 0.17]

Heterogeneity: $Tau^2 = 0.00$; $Chi^2 = 2.03$, df = 2 (P = 0.36); $I^2 = 2\%$
Test for overall effect: Z = 0.98 (P = 0.33)

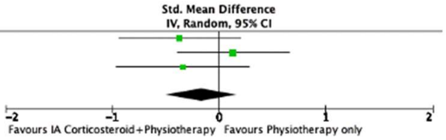

© 2020 Challoumas D et al. *JAMA Network Open.*

eFigure 5. TSA Results for IA Corticosteroid vs No Treatment or Placebo for Early Short-term Pain

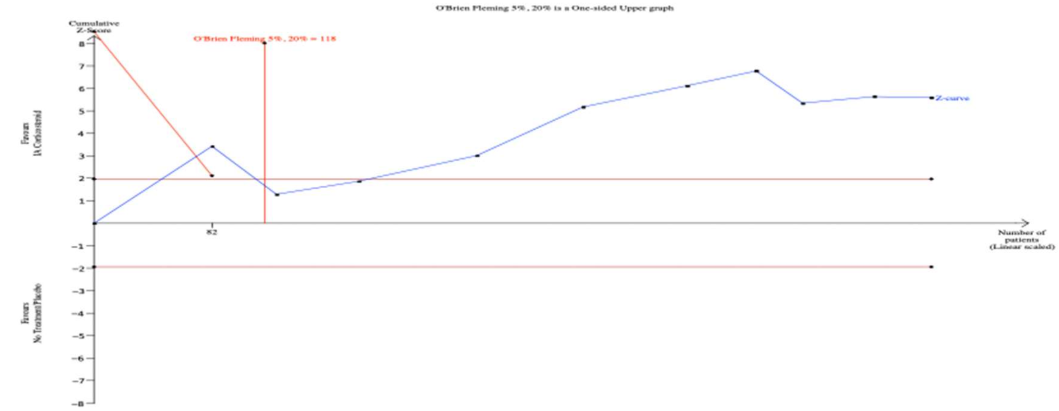

Supplementary Figure 5 **Trial sequential analysis results for intra-articular corticosteroid vs no treatment/placebo early short-term pain.** The two horizontal red lines represent the conventional thresholds for statistical significance (Z=1.96, P<0.05), the vertical red the required information size, the diagonal red line the TSA boundaries (thresholds for statistical significance) and the blue the cumulative amount of information as trials are added. A significant result is denoted by an inter-crossing of the blue and diagonal red lines.

Supplementary Figure 6

eFigure 6. TSA Results for IA Corticosteroid vs No Treatment or Placebo for Late Short-term Pain

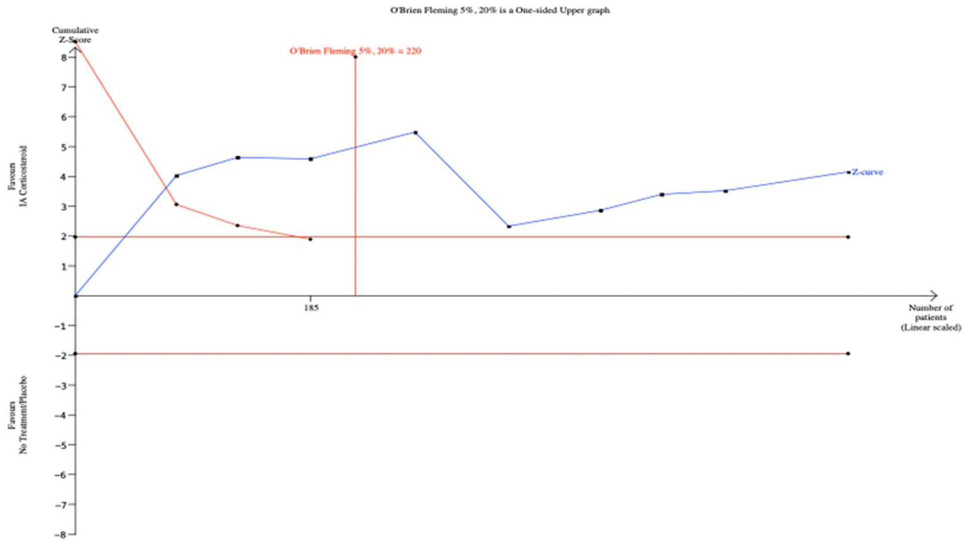

Supplementary Figure 6b. Trial sequential analysis results for intra-articular corticosteroid vs no treatment/placebo late short-term pain. The two horizontal red lines represent the conventional thresholds for statistical significance (*Z*=1.96, *P*<0.05), the vertical red ine the required information size, the diagonal red line the TSA boundaries (thresholds for statistical significance) and the blue the cumulative amount of information as trials are added. A significant result is denoted by an inter-crossing of the blue and diagonal red lines.

© 2020 Challoumas D et al. *JAMA Network Open.*

eFigure 7. Network Forest Plots With Consistency Test for Late Short-term Pain

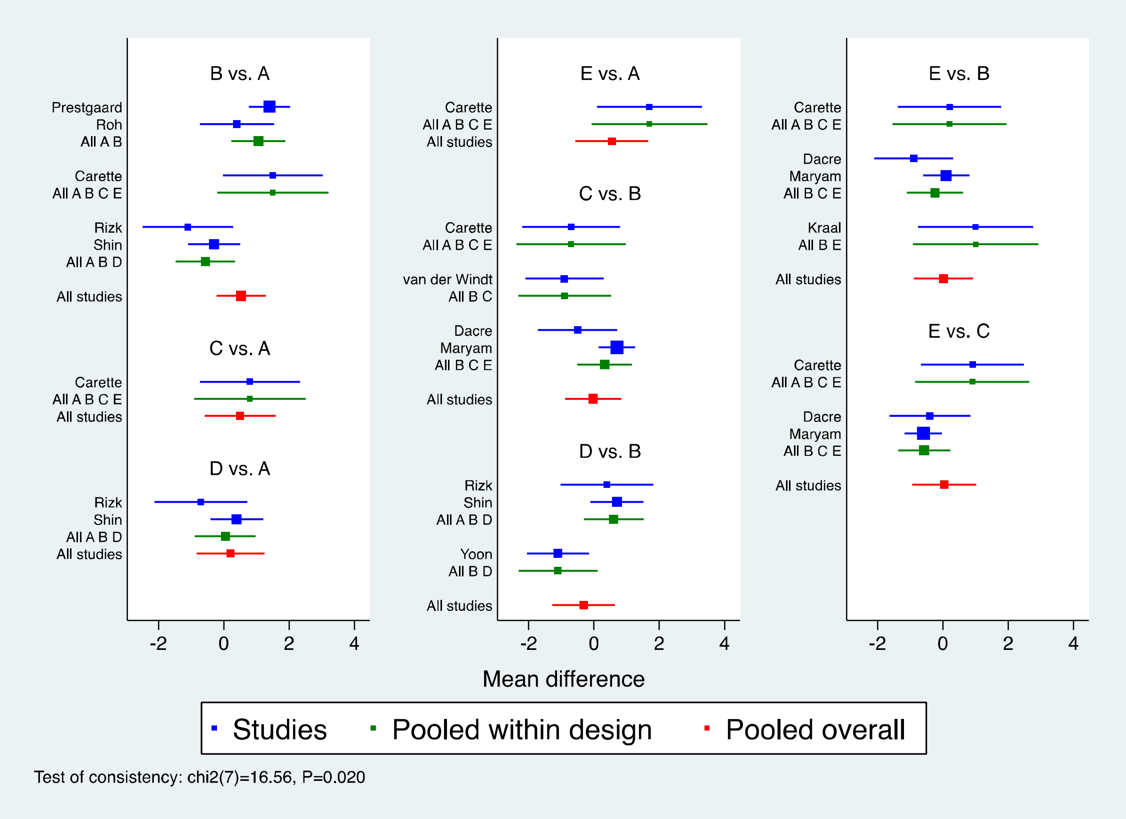

A, no treatment/placebo; B, intra-articular corticosteroid; C, physiotherapy; D, subacromial corticosteroid; E, arthrographic distension plus intra-articular corticosteroid; F, oral corticosteroid

© 2020 Challoumas D et al. *JAMA Network Open.*

eFigure 8. Network Forest Plots With Consistency Test for Mid-term Pain

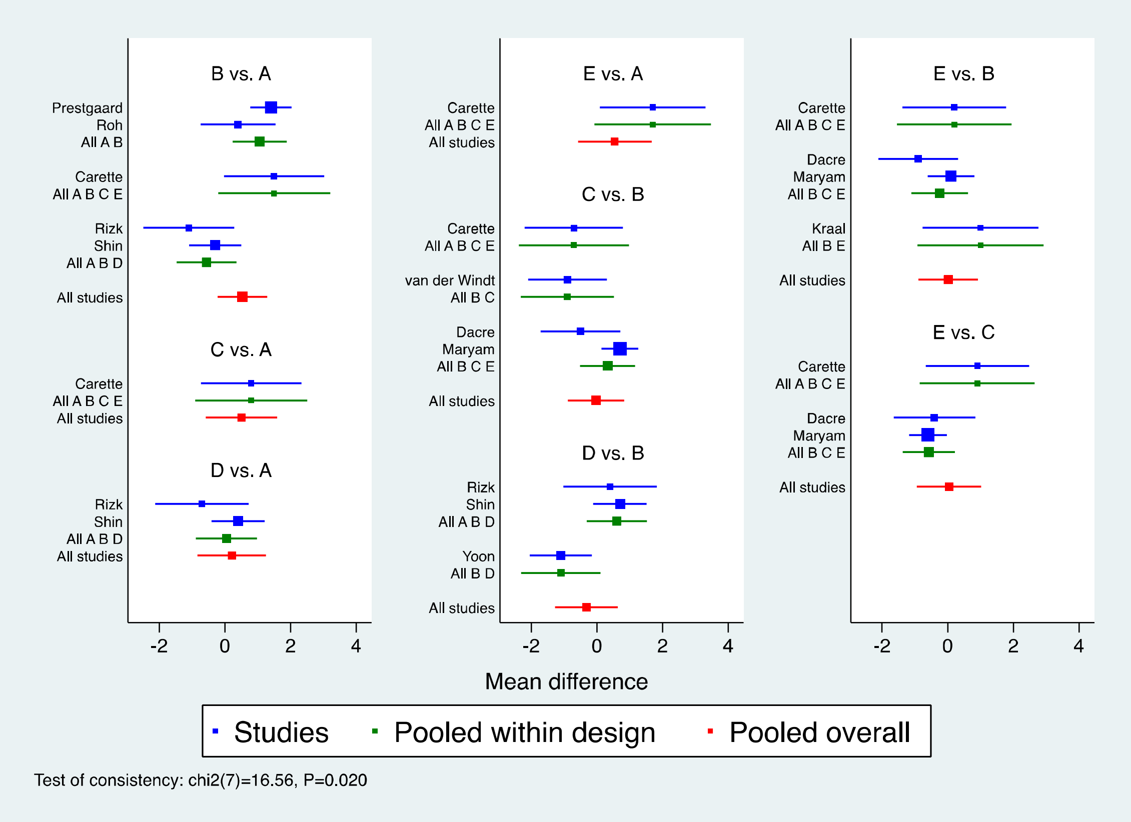

A, no treatment/placebo; B, intra-articular corticosteroid; C, physiotherapy; D, subacromial corticosteroid; E, intra-articular corticosteroid plus physiotherapy.

© 2020 Challoumas D et al. *JAMA Network Open.*
